# Six New Compounds from the Herbaceous Stems of *Ephedra intermedia* Schrenket C. A. Meyer and Their Lung-Protective Activity

**DOI:** 10.3390/molecules29020432

**Published:** 2024-01-16

**Authors:** Xiling Fan, Yangang Cao, Mengnan Zeng, Yingjie Ren, Xiaoke Zheng, Weisheng Feng

**Affiliations:** 1 School of Pharmacy, Henan University of Chinese Medicine, Zhengzhou 450046, China; fxl2020002061@163.com (X.F.); caoyangang1987@126.com (Y.C.); 17320138484@163.com (M.Z.); renyingjie6666@163.com (Y.R.); 2 The Engineering and Technology Center for Chinese Medicine Development of Henan Province, Zhengzhou 450046, China; 3 Co-Construction Collaborative Innovation Center for Chinese Medicine and Respiratory Diseases by Henan & Education Ministry of P.R. China, Zhengzhou 450046, China

**Keywords:** *Ephedra intermedia* Schrenket C. A. Meyer, chemical components, lung-protective activity, MTT assay

## Abstract

Six new compounds, (7*R*,8*S*,8′*R*)-balanophorone (**1**), (7′*S*,8′*R*,8*R*)-yunnanensin A (**2**), (3*S*)-thunberginol C (**3**), (8*R*,8′*R*)-maninsigin B (**4**), (7*S*,8*R*)-4,7,8-dihydroxy-9,9-dimethyl-chroman (**5**), and 4-hydroxy-1-(4-hydroxy-3-methoxyphenyl)butan-1-one (**6**), along with eight known compounds (**7**–**14**), were isolated from the herbaceous stems of *Ephedra intermedia* Schrenket C. A. Meyer. Their structures were elucidated based on their spectroscopic (MS, NMR, IR, and UV) data, and their absolute configurations were determined by comparing their calculated and experimental electronic circular dichroic (ECD) spectra. Moreover, compounds **1** and **3**–**6** were evaluated for their ability to protect human pulmonary epithelial cells (BEAS-2B) from injury induced by lipopolysaccharide (LPS) in vitro. The results showed that compound **6** exhibited a significant protective effect against LPS-induced injury in BEAS-2B, and compound **5** exhibited a slightly protective effect at the concentration of 10 μM.

## 1. Introduction

*Ephedra* has a long history of medicinal use in China, first recorded in the Shennong Materia Medica [1,2], with the efficacy of Fahan Sanhan and Xuanfei Pingchaun. According to the 2020 edition of the Chinese Pharmacopoeia [3,4], the herbaceous stems of *Ephedra intermedia* Schrenket C. A. Meyer, *Ephedra sinica* Stapf, and *Ephedra equisetina* Bunge are considered to be the original plants of Ephedrae Herba (Mahuang). These plants are extensively distributed in Northeast and Northwest China [5,6,7], growing in arid deserts, sandy beach areas, arid mountain slopes, or grassland at elevations ranging from several hundred meters to more than 2000 m above sea level. *Ephedra intermedia* Schrenket C. A. Meyer, also known as “the treasure of the desert,” plays an important role in maintaining the balance of the ecological environment [8].

A previous study on the phytochemical composition of *Ephedra intermedia* Schrenket C. A. Meyer revealed the presence of lignans, flavonoids, terpenes, and organic acids [5]. Furthermore, Zhu et al. found that lignans obtained from *Ephedra equisetina* Bunge had potent anti-asthmatic activity [3]. Jia et al. discovered an amide alkaloid with the ability to attenuate OVA-induced allergic asthma from *Ephedra equisetina* Bunge [9]. Hyperbranched acidic polysaccharide, obtained from *Ephedra sinica* Stapf by Kuang et al. [10], possessed significant immunosuppressive activity. As a well-known traditional Chinese medicine, it has been used to treat colds, bronchial asthma, cough, fever, flu, headache, edema, and allergies for over 5000 years [11,12,13,14,15,16,17,18]. Pharmacological studies have shown that it exhibits many biological activities, including alleviating coughs and asthma, reducing inflammation, regulating blood pressure, and protecting the lungs [11]. 

As part of our continuous work to discover bioactive natural products [19,20,21], six new compounds, together with eight known compounds, were isolated from the herbaceous stems of *Ephedra intermedia* Schrenket C. A. Meyer. These compounds were extracted with a 50% aqueous acetone solution. In this paper, their isolation, structure elucidation, and lung-protective activity against LPS-induced BEAS-2B cell injury are described based on work by our research foundation. Further investigation of the in vivo evidence of the active components and related mechanisms might be required.

## 2. Results and Discussion

### Structure Elucidation of the Isolated Compounds

Compound **1** was obtained as a pale-yellow powder. Its molecular formula of C_22_H_24_O_8_ was determined by the HR-ESI-MS molecular ion peak at *m*/*z* 417.1568 [M + H]^+^ (calc. for 417.1544). The ^1^H NMR data (Table 1) revealed the presence of two 1,3,4-trisubstituted benzene rings [*δ*_H_ 7.66 (1H, dd, *J* = 8.4, 2.0 Hz, H-6′), 7.56 (1H, d, *J* = 2.0 Hz, H-2′), 6.98 (1H, d, *J* = 1.8 Hz, H-2), 6.87 (1H, d, *J* = 8.4 Hz, H-5′), 6.83 (1H, dd, *J* = 8.0, 1.8 Hz, H-6), 6.79 (1H, d, *J* = 8.0 Hz, H-5)], an oxygenated methine [*δ*_H_ 4.71 (1H, d, *J* = 7.9 Hz, H-7)], two oxygenated methylenes [*δ*_H_ 4.33 (1H, dd, *J* = 8.6, 7.3 Hz, H-9′a), 4.21 (1H, dd, *J* = 8.6, 6.7 Hz, H-9′b), 4.01 (1H, dd, *J* = 11.3, 8.1 Hz, H-9a), 3.97 (1H, dd, *J* = 11.3, 6.1 Hz, H-9b)], and two methoxy groups [*δ*_H_ 3.90 (3H, s, 3′-OCH_3_), 3.87 (3H, s, 3-OCH_3_)]. Its ^13^C NMR and DEPT data (Table 1) showed signals for 22 carbons, including one ester carbonyl carbon [*δ*_C_ 172.7 (C-10)], one keto carbonyl group [*δ*_C_ 200.0 (C-7′)], twelve aromatic carbons [*δ*_C_ 153.7 (C-4′), 149.3 (C-3), 149.2 (C-3′), 147.7 (C-4), 133.4 (C-1), 131.1 (C-1′), 125.0 (C-6′), 120.5 (C-6), 116.1 (C-5), 115.9 (C-5′), 111.9 (C-2′), 111.0 (C-2)], one oxygenated methine carbon [*δ*_C_ 85.2 (C-7)], two oxygenated methylene carbons [*δ*_C_ 71.6 (C-9′), 63.3 (C-9)], and two methoxy groups [*δ*_C_ 56.4 (3′, 3-OCH_3_)]. These data corresponded closely to those of 9′-acetoxylariciresinol [22], and the only obvious difference was the presence of a keto carbonyl group (*δ*_C_ 200.0) at C-7′ instead of an oxygenated methine in compound **1**. The comprehensive analysis of the HSQC, HMBC, and ^1^H–^1^H COSY spectra unambiguously confirmed the planar structure of compound **1**. 

**Figure 1:**
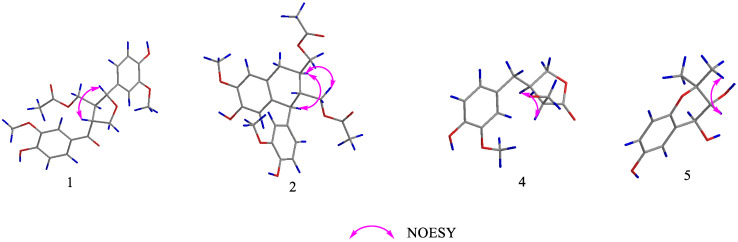
Key NOESY correlations of compounds **1**, **2**, **4**, **5**.

**Figure 2:**
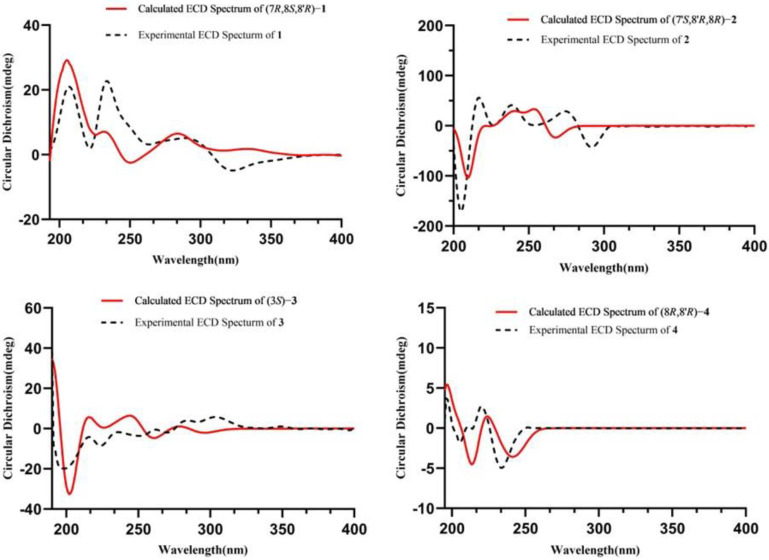
Experimental and calculated ECD spectra of compounds **1**–**4.**

The NOESY experiment (Figure 1) showed that H-7 was correlated with H-8′, which suggested that H-7 and H-8′ were co-facial. The absolute configuration of **1** was established as 7*R*,8*S*,8′*R* by comparing its experimental and calculated ECD spectra (Figure 2). Finally, the structure of **1** was determined and it was termed (7*R*,8*S*,8′*R*)-balanophorone.

Compound **2** was isolated as a white amorphous powder and assigned to have the molecular formula of C_24_H_28_O_8_ based on the HR-ESI-MS ion at *m*/*z* 467.1711 [M + Na]^+^ (Calc. for 467.1676). The analysis of its ^1^H NMR data (Table 1) revealed the presence of a 1,3,4-trisubstituted benzene ring [*δ*_H_ 6.74 (1H, d, *J* = 8.0 Hz, H-5′), 6.64 (1H, d, *J* = 2.0 Hz, H-2′), 6.57 (1H, dd, *J* = 8.0, 2.0 Hz, H-6′)], a 1,3,4,6-tetrasubsituted aromatic ring [*δ*_H_ 6.65 (1H, s, H-2), 6.16 (1H, s, H-5)], two oxygenated methylenes [*δ*_H_ 4.20 (1H, dd, *J* = 11.2, 4.7 Hz, H-9a), 4.07 (1H, dd, *J* = 11.2, 6.2 Hz, H-9b), 4.08 (1H, dd, *J* = 11.5, 3.2 Hz, H-9′a), 3.90 (1H, dd, *J* = 11.5, 3.8 Hz, H-9′b)], and two methoxy groups [*δ*_H_ 3.80 (3H, s, 3-OCH_3_), 3.76 (3H, s, 3′-OCH_3_)].

The ^13^C NMR data (Table 1) together with the HSQC spectrum resolved 24 carbons classified as two ester carbonyl carbons at *δ*_C_ 173.0 (C-10), 172.9 (C-10′); two benzene ring carbons at *δ*_C_ 149.2 (C-3′), 147.5 (C-3), 146.3 (C-4′), 145.5 (C-4), 137.4 (C-1′), 133.4 (C-6), 128.1 (C-1), 123.1 (C-6′), 117.2 (C-5), 116.2 (C-5′), 113.7 (C-2′), 112.3 (C-2); two oxygenated methylene carbons at *δ*_C_ 67.9 (C-9), 64.9 (C-9′); and two methoxy groups at 56.4 (3, 3′-OCH_3_). These data were similar to those of the known compound 9′-acetoxyisolariciresinol [22], except that the hydroxyl group at C-9 was replaced by an acetyl group in compound **2**. 

The relative configuration of compound **2** was determined by NOESY correlations (Figure 1) of H-7′ with H-8 and of H-8 with H-9′. Its absolute configuration was determined to be 7′*S*,8′*R*,8*R* upon comparing its experimental ECD curve (Figure 2) with the calculated one. Finally, the structure of **2** was elucidated and it was named (7′*S*,8′*R*,8*R*)-yunnanensin A.

Compound **3** was obtained as colorless needles, and its molecular formula was assigned as C_15_H_12_O_5_ according to HR-ESI-MS at *m*/*z* 295.0579 [M + Na]^+^ (calc. for 295.0577). The ^1^H NMR data (Table 2) of **3** displayed a 1,3,5,6-tetrasubsituted aromatic ring [*δ*_H_ 6.26 (1H, s, H-5), 6.22 (1H, d, *J* = 2.0 Hz, H-7)] and a 1,4-bisubsituted aromatic ring [*δ*_H_ 7.31 (2H, d, *J* = 8.5 Hz, H-2′, 6′), 6.81 (2H, d, *J* = 8.5 Hz, H-3′, 5′)]. Meanwhile, the signals of *δ*_H_ 5.48 (1H, dd, *J* = 12.2, 3.1 Hz, H-3), 3.25 (1H, dd, *J* = 16.5, 12.2 Hz, H-4a) and 3.01 (1H, dd, *J* = 16.5, 3.1 Hz, H-4b) revealed the presence of an oxygenated methine and a methine. The ^13^C NMR and DEPT 135 spectra (Table 2) revealed the signals of 15 carbons, including an ester carbonyl group at *δ*_C_ 171.8 (C-1); two benzene ring carbons at *δ*_C_ 166.4 (C-6), 165.7 (C-8), 159.1 (C-4′), 143.7 (C-4a), 130.7 (C-1′), 129.0 (C-2′, 6′), 116.3 (C-3′, 5′), 107.9 (C-5), 102.3 (C-7), 101.7 (C-8a); and an oxygenated methine carbon at *δ*_C_ 82.1 (C-3). The interpretation of the NMR data of **3** suggested that compound **3** had the same planar structure as thunberginol C but different stereochemistry [23]. 

SeonJu Park reported the CD data and showed that the characteristic curve for dihydroisocoumarins had a 3*R* configuration, i.e., a negative Cotton effect at 247 nm, a positive Cotton effect at 253 nm, and a negative Cotton effect at 305 nm, which is in contrast to the CD data of compound **3** (a positive Cotton effect at 247 nm, a negative Cotton effect at 253 nm, and a positive Cotton effect at 305 nm). Meanwhile, we determined its absolute configuration to be *S* by comparing its predicted and experimental ECD spectra (Figure 2). Therefore, compound **3** was determined and named as (3*S*)-thunberginol C.

Compound **4** was yielded as a yellow oil with a molecular formula of C_13_H_16_O_5_ as determined by the molecular ion peak at *m*/*z*: 253.1079 [M + H]^+^ (calc. for 253.1071) in its HR-ESI-MS. The ^1^H NMR data (Table 2) of **4** revealed the presence of a 1,3,4-trisubstituted benzene ring [*δ*_H_ 6.81 (1H, d, *J* = 1.8 Hz, H-2), 6.72 (1H, d, *J* = 8.0 Hz, H-5), 6.66 (1H, dd, *J* = 8.0, 1.8 Hz, H-6)], two oxygenated methylenes [*δ*_H_ 4.14 (1H, dd, *J* = 8.6, 7.3 Hz, H-9a), 4.07 (1H, dd, *J* = 8.6, 6.0 Hz, H-9b), 3.96 (1H, dd, *J* = 11.1, 3.9 Hz, H-7′a), 3.89 (1H, dd, *J* = 11.1, 6.2 Hz, H-7′b)], two methines [*δ*_H_ 3.0 (1H, m), 2.97 (1H, m)], a methylene [*δ*_H_ 2.83 (1H, dd, *J* = 14.9, 8.6 Hz), 2.55 (1H, dd, *J* = 14.9, 6.4 Hz)], and a methoxy group [*δ*_H_ 3.84 (3H, s, 3-OCH_3_)]. The ^13^C NMR data (Table 2) of **4** in conjunction with the DEPT 135 spectrum showed the presence of an ester carbonyl group at *δ*_C_ 180.7 (C-9′); one benzene ring carbon at 149.2 (C-3), 146.2 (C-4), 131.9 (C-1), 122.2 (C-6), 116.3 (C-5), 113.3 (C-2); two oxygenated methylene carbons at *δ*_C_ 73.2 (C-9), 59.6 (C-7′); and a methoxy group at 56.4 (3-OCH_3_).

**Figure 3:**
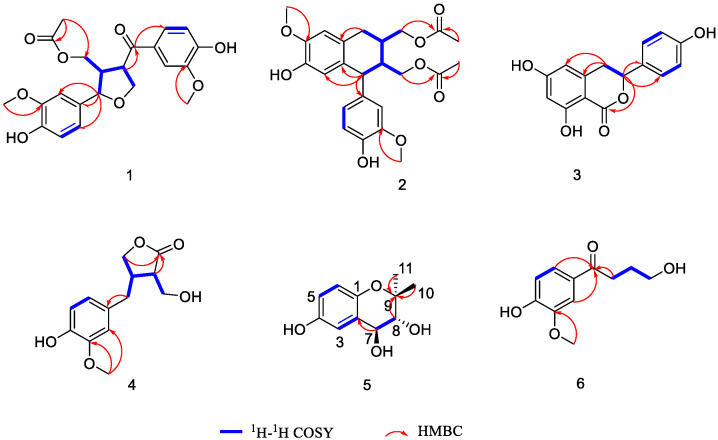
Key ^1^H-^1^H COSY and HMBC correlations of compounds **1**–**6**.

The ^1^H-^1^H COSY correlations (Figure 3) between H-8 and H-9 and between H-7, H-8′, and H-8 and H-7′, as well as the HMBC correlations (Figure 3) from H-7 to C-1 and H-8′/9 to C-9′, indicated the determination of one spin system [CH_2_(9)-CH(8)-CH_2_(7), CH(8)-CH(8′)-CH_2_(7′)] and the presence of a five-membered oxygen ring, and the methylene (C-7) was attached to C-1. In the NOESY spectrum (Figure 1), cross-peaks between H-8 and H-8′ indicated that H-8 and H-8′ were co-facial. The absolute configuration of **4** was established as 8*R*,8′*R* by comparing its experimental and calculated ECD spectra (Figure 2). Thus, compound **4** was elucidated and named (8*R*,8′*R*)-maninsigin B.

Compound **5** was isolated as a colorless oil. The molecular formula was determined to be C_11_H_14_O_4_ according to the HR-ESI-MS at *m*/*z* [M + Na]^+^ 233.0798 (calc. for 233.0784). The ^1^H NMR data (Table 2) of **5** showed the presence of a 1,2,4-trisubsituted benzene ring [*δ*_H_ 6.88 (1H, d, *J* = 2.7 Hz, H-3), 6.59 (1H, dd, *J* = 8.7, 2.7 Hz, H-5), 6.55 (1H, d, *J* = 8.7 Hz, H-6)] and two oxygenated methines [*δ*_H_ 4.39 (1H, d, *J* = 8.4 Hz, H-7), 3.48 (1H, d, *J* = 8.4 Hz, H-8)]. The ^13^C NMR and DEPT 135 spectra (Table 2) revealed the signals of 11 carbons, including four quaternary carbons [*δ*_C_ 151.2 (C-4), 146.8 (C-1), 126.4 (C-2), 79.2 (C-9)], five methines [*δ*_C_ 118.1 (C-6), 117.2 (C-5), 114.8 (C-3)], and two methyl carbons [*δ*_C_ 27.2 (C-11), 19.0 (C-10)].

The positions of two methyl groups were verified by the HMBC correlations (Figure 2) between H-10/11 and C-9. Its NMR data were similar to those of the known compound 3,4-trans-dihydroxy-6-methoxy-2,2-dimethyl-chroman [24], except for the hydroxyl group instead of a methoxy group in compound **5**. The relative configurations of C-7, C-8 were elucidated by its coupling constant (*J* = 8.4 Hz) and the NOESY cross-peak (Figure 1) of H-8 with H-10. The absolute configurations of the asymmetric carbons C-7/8 were determined as 7*S*,8*R* by interpretation of the ECD spectrum (Figure S51) of compound **5**, which displayed a negative Cotton effect at 229 nm [25]. Finally, the structure of **5** was elucidated and it was named (7*S*,8*R*)-4,7,8-dihydroxy-9,9-dimethyl-chroman.

Compound **6** was obtained as a colorless oil and possessed a molecular formula of C_11_H_14_O_4_ as shown in HR-ESI-MS at *m*/*z* [M + Na]^+^ 233.0796 (calc. for 233.0784). There were three aromatic protons at *δ*_H_ 7.56 (1H, dd, *J* = 8.3, 1.6 Hz, H-6′), 7.51 (1H, d, *J* = 1.6 Hz, H-2′), 6.81 (1H, d, *J* = 8.3 Hz, H-5′) in the ^1^H NMR spectrum of **6** (Table 2), indicating the presence of a 1,3,4-trisubstituted benzene ring in **6**. The ^1^H NMR data also included one oxygenated methylene at *δ*_H_ 3.92 (2H, t, *J* = 6.2 Hz, H-4) and a methoxy group at *δ*_H_ 3.88 (3H, s, 3′-OCH_3_). The ^13^C NMR data (Table 2) displayed signals for 11 carbons, of which one was a keto carbonyl group at *δ*_C_ 199.6 (C-1); six were benzene ring carbons at *δ*_C_ 155.4 (C-3′), 149.6 (C-4′), 129.5 (C-1′), 125.1 (C-6′), 116.2 (C-5′), 111.7 (C-2′); and one was an oxygenated methylene carbon at *δ*_C_ 59.0 (C-4).

One partial segment, CH_2_(2)-CH_2_(3)-CH_2_(4), was disclosed by the ^1^H–^1^H COSY spectrum (Figure 3). The HMBC correlations (Figure 3) from H-2 to C-1, from H-2′/6′ to C-1, and from *δ*_H_ 3.88 (3H, s, 3′-OCH_3_) to C-3′ indicated that the methylene (C-2), one keto carbonyl, and a methoxy group were attached to C-1, C-1′, and C-3′, respectively. On the basis of the above data, compound **6** was identified as 4-hydroxy-1-(4-hydroxy-3-methoxyphenyl)butan-1-one, as shown in Figure 1.

**Figure 4:**
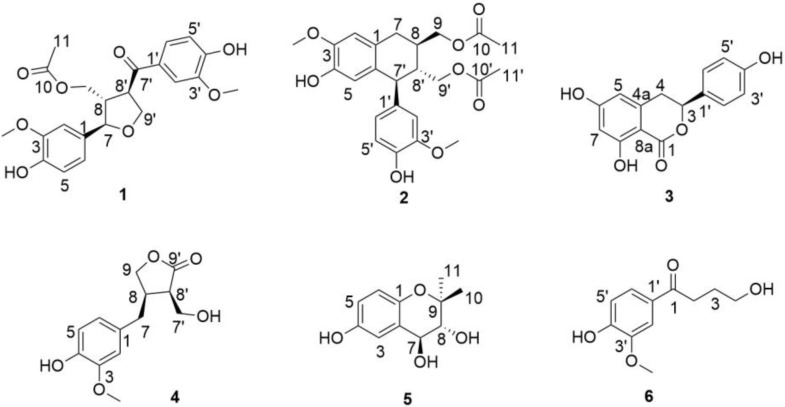
The structures of new compounds **1**–**6**.

Additionally, eight known compounds (Figure 4), namely *N*-(2-hydroxyphenyl)-acetamide [26] (**7**), 4-methoxy-3-hydroxyphenylethanamine [27] (**8**), 3-acetyl-5-methyl-2′-deoxyuridine [28] (**9**), kaempferol [29] (**10**), 3,4′,5,7-tetrahydroxy-3′-methoxyflavanone [30] (**11**), undecenedioic acid [31] (**12**), 2-(4-hydroxy-3-methoxy)-3-(2-hydroxy-5-methoxy)-3-oxo-1-propanol [32] (**13**), and 1,7-bis(4-bis(4-hydroxy-phenyl)-3,5-heptanediol [33] (**14**), were identified via the comparison of their spectroscopic data with those described in the literature.

We have previously evaluated the protective effects against LPS-induced BEAS-2B cell injury of compounds isolated from *C*. *tinctorius* [34]. Meanwhile, some studies have reported that MaHuang possesses the efficacy of Xuanfei Pingchaun and may have potential lung-protective effects. Therefore, to further investigate the lung-protective activity of the new compounds, they were evaluated for their protective effects against LPS-induced BEAS-2B cell injury. As shown in Table 3, the results indicated that compound **6** exhibited a significant protective effect (* *p* < 0.05) against LPS-induced BEAS-2B cell damage at a concentration of 10 μM, and compound **5** exhibited slightly protective activity.

## 3. Materials and Methods

### 3.1. General Experimental Procedures

Optical rotations of new compounds were recorded on a Rudolph AP-IV Polarimeter (Rudolph, MA, USA). Infrared (IR) spectra of the compounds were obtained using a Thermo Nicolet IS 10 spectrometer (Thermo, Waltham, MA, USA). A Bruker Avance Ⅲ 500 MHz spectrometer (Bruker, Ettlingen, Germany) was used for NMR spectra. HR-ESI-MS spectra were obtained on a Bruker maXis HD mass spectrometer (Bruker, Germany). CD spectra were measured on an Applied Photophysics Chirascan qCD spectropolarimeter (AppliedPhotophysics, Leatherhead, Surrey, UK). Semi-preparative high-performance liquid chromatography (HPLC) was conducted on a Saipuruisi LC 52 HPLC system (Saipuruisi, Beijing, China) equipped with two P50 pumps, a UV/VIS50 detector, and a C_18_ column (10 × 250 mm, 5 μm; YMC, Kyoto, Japan). Column chromatography (CC) was performed using silica gel (100–200 mesh, 200–300 mesh, Marine Chemical Industry; Qingdao, China), Toyopearl HW-40F, MCI gel CHP-20 (TOSOH Corp; Tokyo, Japan), ODS gel (50 μm) (YMC Corp; Tokyo, Japan), and Sephadex LH-20 (40–70 μm) (Amersham Pharmacia Biotech AB; Uppsala, Sweden). The chemical reagents (analytical grade) were purchased from HengXing Chemical Reagent Co., Ltd. (Tianjin, China). BEAS-2B cells were purchased from the Shanghai Institutes for Biological Sciences.

### 3.2. Plant Material

The herbaceous stems of *Ephedra intermedia* Schrenket C. A. Meyer were the focal plant material for this study. They were purchased from Xinjiang Xiyu Mocao Chinese Medicinal Materials Development Limited Company, Xinjiang, China, in October 2020, and authenticated by Prof. Cheng-Ming Dong of Henan University of Chinese Medicine. For archival purposes, a specimen (No. 20201111) was deposited at the Department of Natural Medicinal Chemistry, Henan University of Chinese Medicine, Zhengzhou, China.

### 3.3. Extraction and Isolation

The dried herbaceous stems of *Ephedra intermedia* Schrenket C. A. Meyer (45.0 kg) were cut into small segments and extracted with 50% aqueous acetone at room temperature. Then, the evaporation of the solvent under reduced pressure yielded a crude residue (11.1 kg), which was dissolved in water and partitioned by CH_2_Cl_2_, EtOAc, and *n*-BuOH fifteen times, respectively.

The CH_2_Cl_2_ fraction (185.0 g) was subjected to silica gel column chromatography (CC) eluted with a gradient system of petroleum ether–EtOAc (*v*/*v* 50:1–1:1) to yield ten fractions (D1–D10). Fraction D6 (11.8 g) was separated by ODS gel CC elution with a CH_3_OH-H_2_O (*v*/*v* 10:90–100:0) gradient system to give five fractions (D6-1–D6-5). Fraction D6-1 (837.0 mg) was subjected to the Sephadex LH-20 CC with CH_3_OH-H_2_O (*v*/*v* 70:30) as a mobile phase to afford six fractions (D6-1-1–D6-1-6), and D6-1-4 (180.6 mg) was rechromatographed with silica gel CC eluted with a CH_2_Cl_2_-CH_3_OH (*v*/*v* 300:1–20:1) gradient system to give three fractions (D6-1-4-1–D6-1-4-3). Then, D6-1-4-1 was purified by semi-preparative HPLC (CH_3_CN-H_2_O *v*/*v* 17:83) to produce compound **7** (9.0 mg, *t*_R_ = 23.5 min). Fraction D6-2 (2.0 g) was separated via Toyopearl HW-40F CC using a gradient elution of CH_3_OH to produce five fractions (D6-2-1–D6-2-5), and D6-2-4 was purified by preparative thin layer chromatograph with EtOAc-CH_3_OH (*v*/*v* 25:1) to yield compound **8** (5.1 mg). Fraction D7 (17.2 g) was subjected to ODS gel CC eluted with a CH_3_OH-H_2_O (*v*/*v* 10:90–100:0) gradient system to give ten fractions (D7-1–D7-10). Five fractions (D7-6-1–D7-6-5) were obtained by D7-6 (1.2 g) via Toyopearl HW-40F CC using a gradient elution of CH_3_OH-H_2_O (*v*/*v* 70:30), and compound **12** (2.4 mg, *t*_R_ = 46.5 min) was obtained from D7-6-2 (365.6 mg) by semi-preparative HPLC (CH_3_OH-H_2_O *v*/*v* 60:40). Fraction D8 (12.0 g) was applied to MCI gel CHP-20 CC eluted with a gradient system of CH_3_OH-H_2_O (*v*/*v* 10:90–100:0) to give six fractions (D8-1–D8-6). D8-5 (1.4 g) was passed through silica gel CC eluted with a CH_2_Cl_2_-CH_3_OH (*v*/*v* 250:1–10:1) gradient system to obtain seven fractions (D8-5-1–D8-5-7). Compounds **2** (9.7 mg, *t*_R_ = 25.2 min) and **13** (3.0 mg, *t*_R_ = 14.6 min) were produced from D8-5-1 (237.3 mg) using semi-preparative HPLC (CH_3_CN-H_2_O *v*/*v* 41:59). D8-5-2 (50.6 mg) was separated by semi-preparative HPLC (CH_3_OH-H_2_O *v*/*v* 38:62) to give compound **1** (5.5 mg, *t*_R_ = 35.1 min).

The EtOAc fraction (1.4 kg) was subjected to silica gel CC eluted with a gradient system of CH_2_Cl_2_-CH_3_OH (*v*/*v* 25:1 12:1) and EtOAc-CH_3_OH (*v*/*v* 35:1 15:1 5:1) to yield eleven fractions (E1–E11). E3 (12.4 g) was applied to Toyopearl HW-40F CC using CH_3_OH to afford six fractions (E3-1–E3-6). E3-4 (400.6 mg) was subjected to silica gel CC eluting with CH_2_Cl_2_-CH_3_OH (*v*/*v* 250:1–10:1) to obtain five fractions (E3-4-1–E3-4-5), and then E3-4-3 (101.5 mg) was purified by semi-preparative HPLC with CH_3_OH-H_2_O (*v*/*v* 40:60) as the mobile phase to obtain compound **3** (2.5 mg, *t*_R_ = 31.2 min) and compound **11** (5.53 mg, *t*_R_ = 36.7 min). E3-5 (166.0 mg) was separated by silica gel CC, which was eluted with a gradient system of CH_2_Cl_2_-CH_3_OH (*v*/*v* 280:1 240:1 220:1 180:1 150:1) and purified by semi-preparative HPLC (CH_3_OH-H_2_O *v*/*v* 55:45) to yield compound **5** (6.5 mg, *t*_R_ = 26.6 min) and compound **10** (29.4 mg, *t*_R_ = 35.5 min). E4 (24.3 g) was applied to MCI gel CHP-20 CC and eluted with a gradient system of CH_3_OH-H_2_O (*v*/*v* 10:90–100:0) to give five fractions (E4-1–E4-5). E4-3 (6.45 mg) was separated by silica gel CC with CH_2_Cl_2_-CH_3_OH (*v*/*v* 450:1–50:1) to yield four fractions (E4-3-1–E4-3-4). E4-3-1 (3.4 g) was subjected to ODS gel CC eluted with a CH_3_OH-H_2_O (*v*/*v* 10:90–100:0) gradient system to give four fractions (E4-3-1-1–E4-3-1-4). E4-3-1-3 was purified by preparative thin layer chromatography with CH_2_Cl_2_-CH_3_OH (*v*/*v* 35:1) to yield compound **6** (13.0 mg). Compound **14** (4.0 mg, *t*_R_ = 22.2 min) was obtained from E4-3-1-4 using semi-preparative HPLC (CH_3_CN-H_2_O *v*/*v* 14:86). E8 (89.3 g) was eluted by CH_3_OH-H_2_O (*v*/*v* 10:90–100:0) on MCI gel CHP-20 CC to give six fractions (E8-1–E8-6). Then, E8-2 (5.3 g) was applied to Toyopearl HW-40F CC using CH_3_OH-H_2_O (*v*/*v* 10:90–100:0) to afford five fractions (E8-2-1–E8-2-5). Compounds **4** (1.8 mg, *t*_R_ = 29.8 min) and **9** (12.6 mg, *t*_R_ = 22.5 min) were obtained from E8-2-2 (1.14 g) by silica gel CC, eluted with EtOAc-CH_3_OH (*v*/*v* 400:1–20:1), and then purified by semi-preparative HPLC (CH_3_CN-H_2_O *v*/*v* 15:85).

Compound **1**: a pale-yellow powder, ${\left[\alpha \right]}_{D}^{20}$ −10.45 (*c* 0.01 CH_3_OH); UV (CH_3_OH) *λ*_max_(log*ε*): 203, 231, 280, 306 nm; IR (iTR): *ν*_max_: 3370, 1599, 1517 cm^−1^; ^1^H (CD_3_OD, 500 MHz); and ^13^C (CD_3_OD, 125 MHz); for NMR data, see Table 1; HR-ESI-MS *m*/*z* 417.1568 [M + H]^+^ (Calc. for 417.1544, StdDev 1.26 ppm).

Compound **2**: white amorphous powder, ${\left[\alpha \right]}_{D}^{20}$ +159.041 (c 0. 017 CH_3_OH); UV (CH_3_OH) *λ*_max_(log*ε*): 209, 283 nm; IR (iTR): *ν*_max_: 3393, 1734, 1513, 1262, 1034 cm^−1^; ^1^H (CD_3_OD, 500 MHz); and ^13^C (CD_3_OD, 125 MHz); for NMR data, see Table 1; HR-ESI-MS *m*/*z* 467.1711 [M + Na]^+^ (Calc. for 467.1676, StdDev 1.26 ppm).

Compound **3**: colorless needles, ${\left[\alpha \right]}_{D}^{20}$ +9.379 (*c* 0.01 CH_3_OH); UV (CH_3_OH) *λ*_max_(log*ε*): 202, 270, 302 nm; IR (iTR): *ν*_max_: 3390, 1648, 1017 cm^−1^; ^1^H (CD_3_OD, 500 MHz); and ^13^C (CD_3_OD, 125 MHz); for NMR data, see Table 2; HR-ESI-MS *m*/*z* 295.0579 [M + Na]^+^ (Calc. for 295.0577, StdDev 1.26 ppm).

Compound **4**: yellow oil, ${\left[\alpha \right]}_{D}^{20}$ +85.669 (*c* 0.09 CH_3_OH); UV (CH_3_OH) *λ*_max_(log*ε*): 203, 226, 281 nm; IR (iTR): *ν*_max_: 3272, 2946, 1450, 1030 cm^−1^; ^1^H (CD_3_OD, 500 MHz); and ^13^C (CD_3_OD, 125 MHz); for NMR data, see Table 2; HR-ESI-MS *m*/*z*: 253.1079 [M + H]^+^ (Calc. for 253.1071, StdDev 1.26 ppm).

Compound **5**: colorless oil, ${\left[\alpha \right]}_{D}^{20}$ +85.669 (c 0.007 CH_3_OH); UV (CH_3_OH) *λ*_max_(log*ε*): 209, 229, 299 nm; IR (iTR): *ν*_max_: 3374, 1493, 1030 cm^−1^; ^1^H (CD_3_OD, 500 MHz); and ^13^C (CD_3_OD, 125 MHz); for NMR data, see Table 2; HR-ESI-MS [M + Na]^+^ *m*/*z* 233.0798 (Calc. for 233.0784, StdDev 1.26 ppm).

Compound **6**: colorless oil, UV (CH_3_OH) *λ*_max_(log*ε*): 201, 225, 277 nm; IR (iTR): *ν*_max_: 3370, 1449, 1027 cm^−1^; ^1^H (CD_3_OD, 500 MHz); and ^13^C (CD_3_OD, 125 MHz); for NMR data, see Table 2; HR-ESI-MS [M + Na]^+^ *m*/*z* 233.0796 (Calc. for 233.0784, StdDev 1.26 ppm).

### 3.4. Biological Activity

#### 3.4.1. Cell Culture

The BEAS-2B cells, purchased from the Shanghai Institutes for Biological Sciences, were cultured in DMEM medium containing 10% FBS, 1% penicillin, 1% phytomycin, and 5% CO_2_ at 37 °C in a constant-temperature incubator.

#### 3.4.2. MTT Assay

The BEAS-2B cells cultured to the log phase were plated into 96-well flat-bottomed culture plates at a concentration of 4 × 10^3^ cells per well. Then, the cells were divided into a normal group (NC), a model group (M, 0.5 µg mL^−1^), and a group for each compound (compounds **1**, **3**–**6**, 10 μM, 10 μM + LPS 0.5 µg mL^−1^). After incubation for 24 h, 20 μL MTT (5 mg/mL) was added to each well and the plates were incubated for 4 h. Then, dimethyl sulfoxide (DMSO, 150 μL) was added, and the solution was removed. Finally, the optical density (OD) values were measured at 490 nm with a microplate reader (Thermo Scientific, Boston, MA, USA).

#### 3.4.3. Statistical Analysis

All data were analyzed using the SPSS software version 26.0 and presented as the mean ± standard deviation ( $\bar{x}$ ± sd). A one-way analysis of variance (one-way ANOVA) was used for comparisons between groups. The differences were considered significant when *p* < 0.05 and very significant when *p* < 0.01.

### 3.5. ECD Spectra Calculations

Conformational analyses of compounds **1**–**4** were performed using the GMMX software 6.0, which uses the MMFF94 force field. After they were optimized at the B3LYP/6-31G (d,p) level, the conformers with a Boltzmann distribution ≥ 1% were imported into the Gaussian 16 software. Then, calculations of the ECD curves were performed with the TDDFT method at the B3LYP/6-311G (d,p) level in CH_3_OH solution. Finally, based on the Boltzmann weighting of each conformer with a half-band width of 0.25 eV, the ECD spectra were simulated by the SpecDis 1.70.1 software.

## 4. Conclusions

In the present study, a comprehensive phytochemical investigation was conducted to isolate 14 compounds from the herbaceous stems of *Ephedra intermedia* Schrenket C. A. Meyer. Their structures were characterized through extensive spectroscopic analysis (NMR, MS, UV, IR, and ECD). They included six new compounds, (7*R*,8*S*,8′*R*)-balanophorone (**1**), (7′*S*,8′*R*,8*R*)-yunnanensin A (**2**), (3*S*)-thunberginol C (**3**), (8*R*,8′*R*)-maninsigin B (**4**), (7*S*,8*R*)-4,7,8-dihydroxy-9,9-dimethyl-chroman (**5**), and 4-hydroxy-1-(4-hydroxy-3-methoxyphenyl)butan-1-one (**6**), as well as eight known compounds (**7**–**14**). Based on the activity study of *Ephedra intermedia* reported, we evaluated, for the compounds obtained from the herbaceous stems of *Ephedra intermedia*, the protective effects against human pulmonary epithelial cell (BEAS-2B) injury induced by lipopolysaccharide (LPS) in vitro. The results showed that compound **6** exhibited a significant protective effect against LPS-induced BEAS-2B cell injury, and compound **5** exhibited a slightly protective effect at the concentration of 10 μM. Next, we will attempt to enrich compounds **5** and **6** for a more in-depth mechanistic exploration and to discover more bioactive compounds, and we will carry out further research on the mechanisms of potential compounds for the treatment of lung injury.

## Figures and Tables

**Table 1:** ^1^H NMR (500 MHz) and ^13^C NMR (125 MHz) data of compounds **1** and **2** in CD_3_OD.

No.	1		2	
	*δ*_H_	*δ*_C_	*δ*_H_	*δ*_C_
1		133.4		128.1
2	6.98 (1H, d, 1.8)	111.0	6.65 (1H, s)	112.3
3		149.3		147.5
4		147.7		145.5
5	6.79 (1H, d, 8.0)	116.1	6.16 (1H, s)	117.2
6	6.83 (1H, dd, 8.0, 1.8)	120.5		133.4
7	4.71 (1H, d, 7.9)	85.2	2.83 (1H, dd, 15.8, 5.2) 2.77 (1H, dd, 15.8, 10.4)	33.6
8	2.88 (1H, m,)	51.6	2.20 (1H, m)	37.1
9	4.01 (1H, dd, 11.3, 8.1) 3.97 (1H, dd, 11.3, 6.1)	63.3	4.20 (1H, dd, 11.2, 4.7) 4.07 (1H, dd, 11.2, 6.2)	67.9
10		172.7		173.0
11	1.56 (3H, s)	20.2	2.0 (3H, s)	20.8
3-OCH_3_	3.87 (3H, s)	56.4	3.80 (3H, s)	56.4
1′		131.1		137.4
2′	7.56 (1H, d, 2.0)	111.9	6.64 (1H, d, 2.0)	113.7
3′		149.2		149.2
4′		153.7		146.3
5′	6.87 (1H, d, 8.4)	115.9	6.74 (1H, d, 8.0)	116.2
6′	7.66 (1H, dd, 8.4, 2.0)	120.5	6.57 (1H, dd, 8.0, 2.0)	123.1
7′		200.0	3.78 (1H, m)	48.5
8′	4.48 (1H, m)	48.0	2.00 (1H, m)	45.0
9′	4.33 (1H, dd, 8.6, 7.3) 4.21 (1H, dd, 8.6, 6.7)	71.6	4.08 (1H, dd, 11.5, 3.2) 3.90 (1H, dd, 11.5, 3.8)	64.9
10′				172.9
11′			2.0 (3H, s)	20.6
3′-OCH_3_	3.90 (3H, s)	56.4	3.76 (3H, s)	56.4

**Table 2:** ^1^H NMR (500 MHz) and ^13^C NMR (125 MHz) data of compounds **3**–**6** in CD_3_OD.

No.	3		4		5		6	
	*δ*_H_	*δ*_C_	*δ*_H_	*δ*_C_	*δ*_H_	*δ*_C_	*δ*_H_	*δ*_C_
1		171.8		131.9		146.8		199.6
2			6.81 (1H, d, 1.8)	113.3		126.4	3.14 (2H, t, 6.2)	41.6
3	5.48 (1H, dd, 12.2, 3.1)	82.1		149.2	6.88 (1H, d, 2.7)	114.8	1.89 (2H, brs)	24.2
4	3.25 (1H, dd, 16.5, 12.2) 3.01(1H, dd, 16.5, 3.1)	35.9		146.2		151.2	3.92 (2H, t, 6.2)	59.0
4a		143.7						
5	6.26 (1H, s)	107.9	6.72 (1H, d, 8.0)	116.3	6.59 (1H, dd, 8.7, 2.7)	117.2		
6		166.4	6.66 (1H, dd, 8.0, 1.8)	122.2	6.55 (1H, d, 8.7)	118.1		
7	6.22 (1H, d, 2.0)	102.3	2.83 (1H, dd, 14.9, 8.6) 2.55 (1H, dd, 14.9, 6.4)	33.8	4.39 (1H, d, 8.4)	70.4		
8		165.7	3.00 (1H, m)	40.7	3.48 (1H, d, 8.4)	77.3		
8a		101.7						
9			4.14 (1H, dd, 8.6, 7.3) 4.07 (1H, dd, 8.6, 6.0)	73.2		79.2		
10					1.13 (3H, s)	19.0		
11					1.38 (3H, s)	27.2		
3-OCH_3_			3.84 (3H, s)	56.4				
1′		130.7						129.5
2′	7.31 (1H, d, 8.5)	129.0					7.51 (1H, d, 1.6)	111.7
3′	6.81 (1H, d, 8.5)	116.3						155.4
4′		159.1						149.6
5′	6.81 (1H, d, 8.5)	116.3					6.81 (1H, d, 8.3)	116.2
6′	7.31 (1H, d, 8.5)	129.0					7.56 (1H, dd, 8.3, 1.6)	125.1
7′			3.96 (1H, dd, 11.1, 3.9) 3.89 (1H, dd, 11.1, 6.2)	59.6				
8′			2.97 (1H, m)	47.0				
9′				180.7				
3′-OCH_3_							3.88 (3H, s)	56.3

**Table 3:** The effects of compounds **1**, **3**–**6** on BEAS-2B cells by LPS.

Group	Dose (µM)	Cell Viability (%)
NC	-	1.071 ± 0.051
M	0.5 µg mL^−1^	1.000 ± 0.048 ^##^
**1**	0.5 µg mL^−1^, 10 µM	0.964 ± 0.026
**3**	0.5 µg mL^−1^, 10 µM	0.959 ± 0.020
**4**	0.5 µg mL^−1^, 10 µM	0.931 ± 0.023
**5**	0.5 µg mL^−1^, 10 µM	1.023 ± 0.042
**6**	0.5 µg mL^−1^, 10 µM	1.047 ± 0.041 *

(NC: blank control group; M: model group. ^##^ *p* < 0.01 compared with the NC group; * *p* < 0.05 compared with the M group).

## Data Availability

Data are contained within the article or Supplementary Materials.

## Supplementary Materials

The following supporting information can be downloaded at: www.mdpi.com/xxx/s1.
