# Supplementary material for: Six New Compounds from the Herbaceous Stems of Ephedra intermedia Schrenket C. A. Meyer and Their Lung-Protective Activity
Source: Molecules. 2024 Jan 16;29(2):0. doi: 10.3390/molecules29020432 (PMC11154346; doi:10.3390/molecules29020432)
Supplement: Supplementary file 1 [file molecules-29-00432-s001.zip › molecules-2812761-supplementary.pdf]

Six New Compounds from the Herbaceous Stems of *Ephedra intermedia* Schrenket C. A. Meyer and Their Lung-Protective Activity

Xiling Fan [1,2], Yangang Cao [1,2], Mengnan Zeng [1,2], Yingjie Ren [1,2], Xiaoke Zheng [1,2,*] and Weisheng Feng [1,2,3,*]

1 School of Pharmacy, Henan University of Chinese Medicine, Zhengzhou 450046, China; fxl2020002061@163.com (X.F.); caoyangang1987@126.com (Y.C.); 17320138484@163.com (M.Z.); renyingjie6666@163.com (Y.R.)

2 The Engineering and Technology Center for Chinese Medicine Development of Henan Province, Zhengzhou 450046, China

3 Co-Construction Collaborative Innovation Center for Chinese Medicine and Respiratory Diseases by Henan & Education Ministry of P.R. China, Zhengzhou 450046, China

* Correspondence: zhengxk.2006@163.com (X.Z.); fwsh@hactcm.edu.cn (W.F.)

Contents

Figure S1. ^{1}H NMR spectrum (500MHz, CD_3OD) of **1**

Figure S2. ^{13}C NMR spectrum (125MHz, CD_3OD) of **1**

Figure S3. DEPT spectrum of **1**

Figure S4. ^{1}H-^{1}H COSY spectrum of **1**

Figure S5. HSQC spectrum of **1**

Figure S6. HMBC spectrum of **1**

Figure S7. NOESY spectrum of **1**

Figure S8. HR-ESI-MS spectrum of compound **1**

Figure S9. UV spectrum of **1**

Figure S10. IR spectrum of **1**

Figure S11. ^{1}H NMR spectrum (500MHz, CD_3OD) of **2**

Figure S12. ^{13}C NMR spectrum (125MHz, CD_3OD) of **2**

Figure S13. DEPT spectrum of **2**

Figure S14. ^{1}H-^{1}H COSY spectrum of **2**

Figure S15. HSQC spectrum of **2**

Figure S16. HMBC spectrum of **2**

Figure S17. NOESY spectrum of **2**

Figure S18. HR-ESI-MS spectrum of compound **2**

Figure S19. UV spectrum of **2**

Figure S20. IR spectrum of **2**

Figure S21. ^{1}H NMR spectrum (500MHz, CD_3OD) of **3**

Figure S22. ^{13}C NMR spectrum (125MHz, CD_3OD) of **3**

Figure S23. DEPT spectrum of **3**

Figure S24. ^{1}H-^{1}H COSY spectrum of **3**

Figure S25. HSQC spectrum of **3**

Figure S26. HMBC spectrum of **3**

Figure S27. NOESY spectrum of **3**

Figure S28. HR-ESI-MS spectrum of compound **3**

Figure S29. UV spectrum of **3**

Figure S30. IR spectrum of **3**

Figure S31. ^{1}H NMR spectrum (500MHz, CD_3OD) of **4**

Figure S32. ^{13}C NMR spectrum (125MHz, CD_3OD) of **4**

Figure S33. DEPT spectrum of **4**

Figure S34. ^{1}H-^{1}H COSY spectrum of **4**

Figure S35. HSQC spectrum of **4**

Figure S36. HMBC spectrum of **4**

Figure S37. NOESY spectrum of **4**

Figure S38. HR-ESI-MS spectrum of compound **4**

Figure S39. UV spectrum of **4**

Figure S40. IR spectrum of **4**

Figure S41. ^{1}H NMR spectrum (500MHz, CD_3OD) of **5**

Figure S42. ^{13}C NMR spectrum (125MHz, CD_3OD) of **5**

Figure S43. DEPT spectrum of **5**

Figure S44. ^{1}H-^{1}H COSY spectrum of **5**

Figure S45. HSQC spectrum of **5**

Figure S46. HMBC spectrum of **5**

Figure S47. NOESY spectrum of **5**

Figure S38. HR-ESI-MS spectrum of compound **5**

Figure S49. UV spectrum of **5**

Figure S50. IR spectrum of **5**

Figure S51. Experimental ECD spectrum of **5**

Figure S52. ^{1}H NMR spectrum (500MHz, CD_3OD) of **6**

Figure S53. ^{13}C NMR spectrum (125MHz, CD_3OD) of **6**

Figure S54. DEPT spectrum of **6**

Figure S55. ^{1}H-^{1}H COSY spectrum of **6**

Figure S56. HSQC spectrum of **6**

Figure S57. HMBC spectrum of **6**

Figure S58. HR-ESI-MS spectrum of compound **6**

Figure S59. UV spectrum of **6**

Figure S60. IR spectrum of **6**

Figure S61. ^{1}H NMR spectrum (500MHz, CD_3OD) of **7**

Figure S62. ^{13}C NMR spectrum (125MHz, CD_3OD) of **7**

Figure S63. ^{1}H NMR spectrum (500MHz, CD_3OD) of **8**

Figure S64. ^{13}C NMR spectrum (125MHz, CD_3OD) of **8**

Figure S65. ^{1}H NMR spectrum (500MHz, CD_3OD) of **9**

Figure S66. ^{13}C NMR spectrum (125MHz, CD_3OD) of **9**

Figure S67. ^{1}H NMR spectrum (500MHz, CD_3OD) of **10**

Figure S68. ^{13}C NMR spectrum (125MHz, CD_3OD) of **10**

Figure S69. ^{1}H NMR spectrum (500MHz, CD_3OD) of **11**

Figure S70. ^{13}C NMR spectrum (125MHz, CD_3OD) of **11**

Figure S71. ^{1}H NMR spectrum (500MHz, CD_3OD) of **12**

Figure S72. ^{13}C NMR spectrum (125MHz, CD_3OD) of **12**

Figure S73. ^{1}H NMR spectrum (500MHz, CD_3OD) of **13**

Figure S74. ^{13}C NMR spectrum (125MHz, CD_3OD) of **13**

Figure S75. ^{1}H NMR spectrum (500MHz, CD_3OD) of **14**

Figure S76. ^{13}C NMR spectrum (125MHz, CD_3OD) of **14**

Table S1. Energy for ECD calculation of compound 1-5

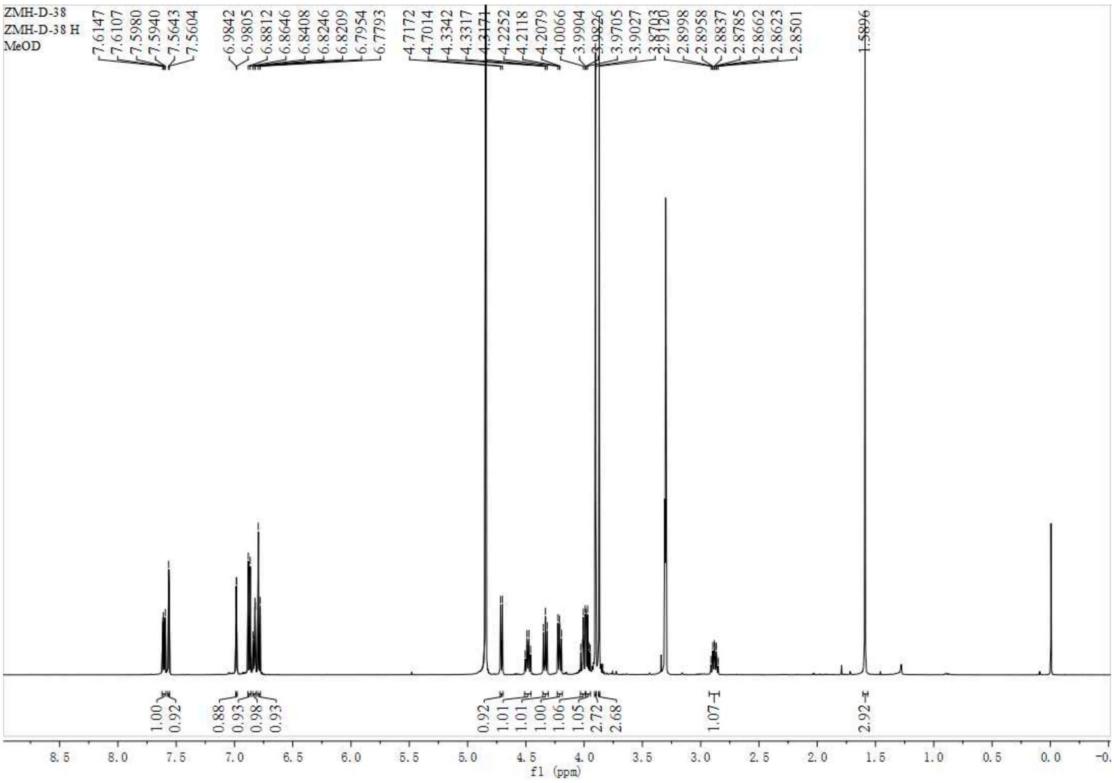

Figure S1. ^{1}H NMR spectrum (500MHz, CD_3OD) of **1**

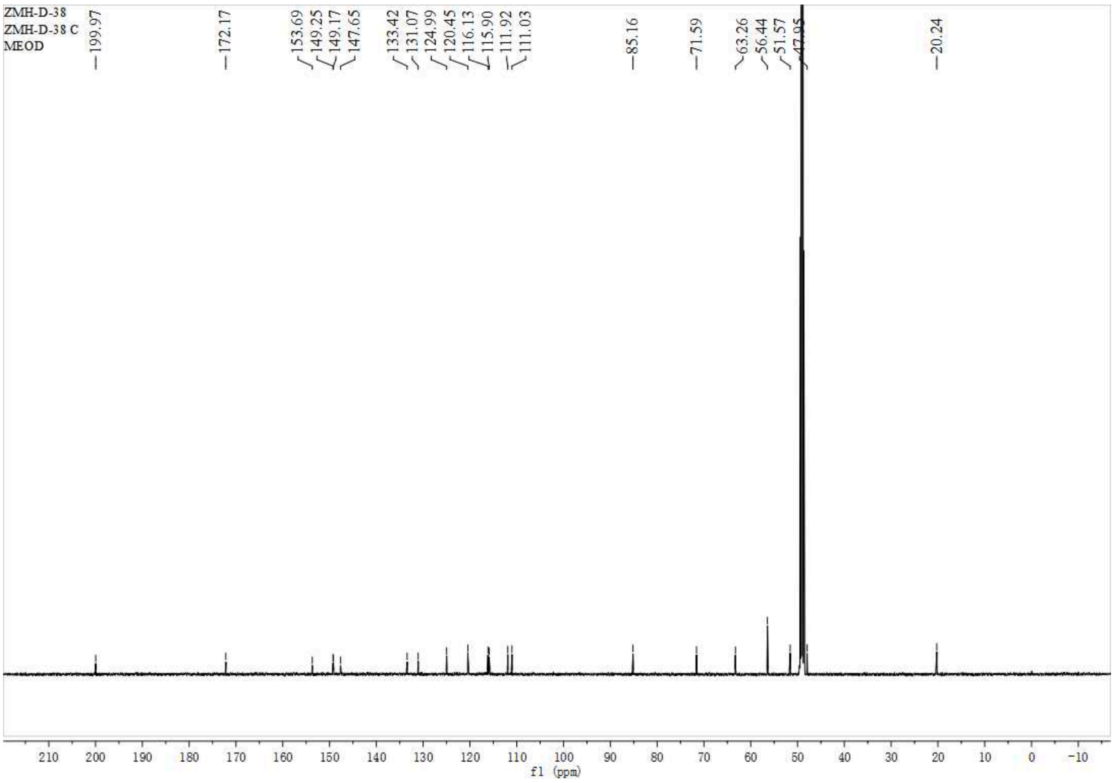

Figure S2. ^{13}C NMR spectrum (125MHz, CD_3OD) of **1**

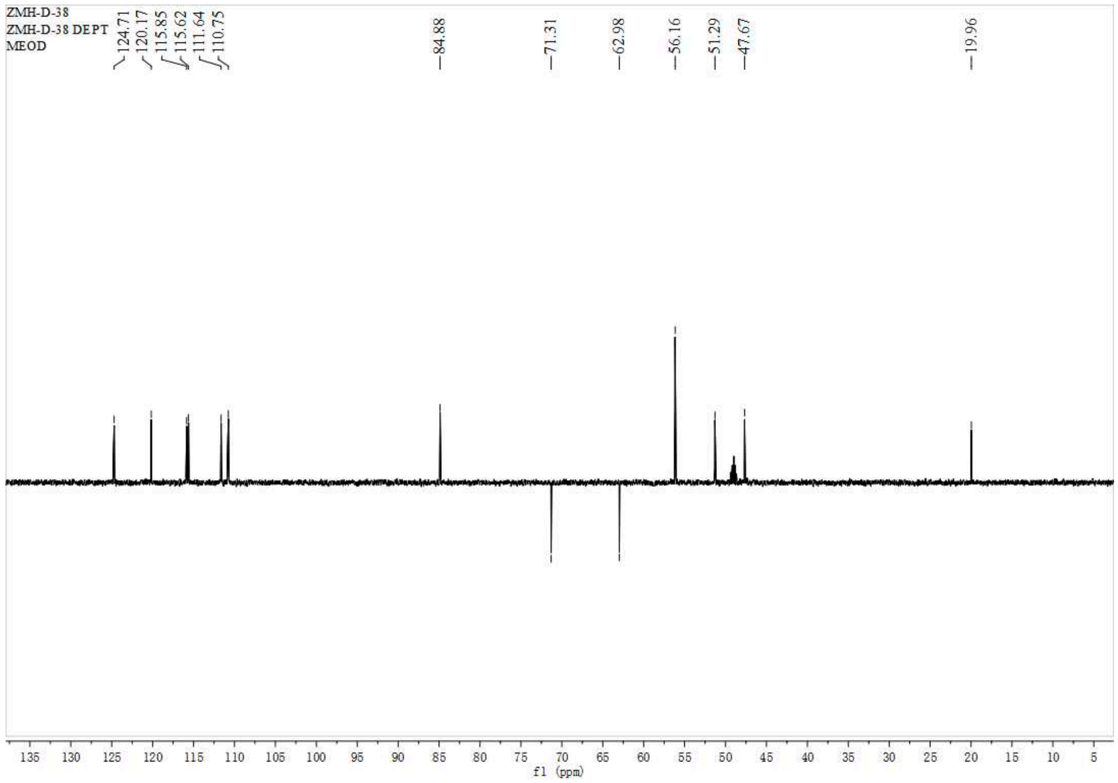

Figure S3. DEPT spectrum of **1**

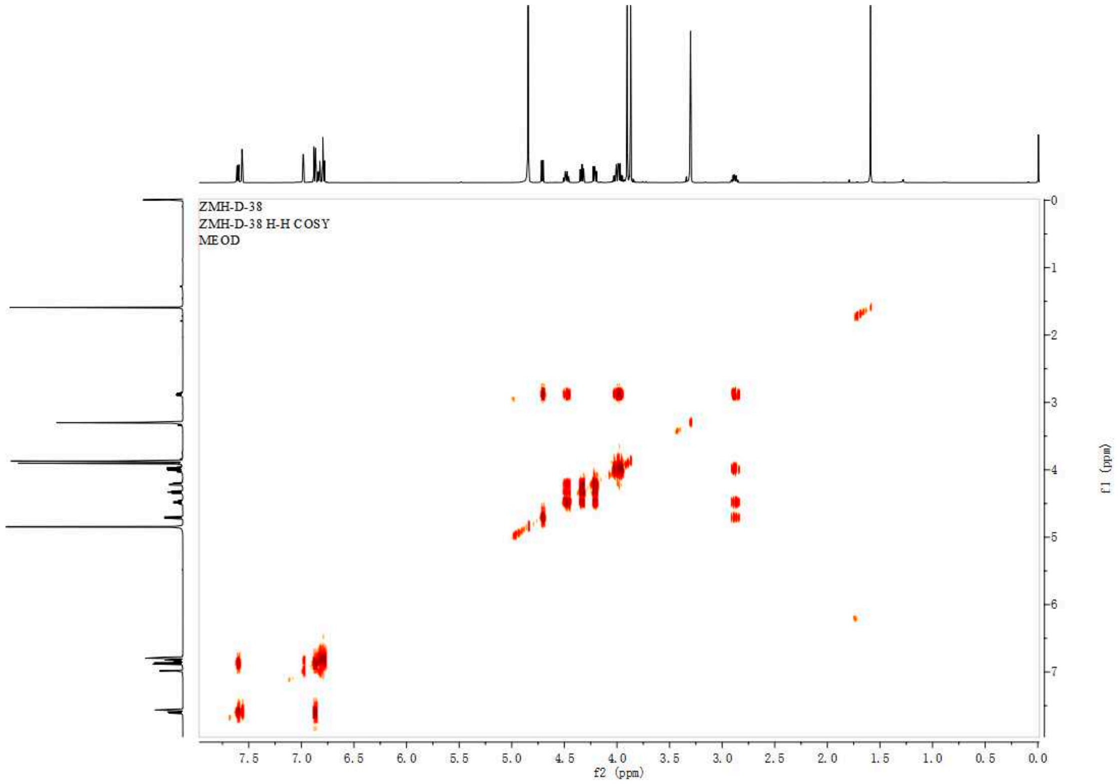

Figure S4. ^{1}H-^{1}H COSY spectrum of **1**

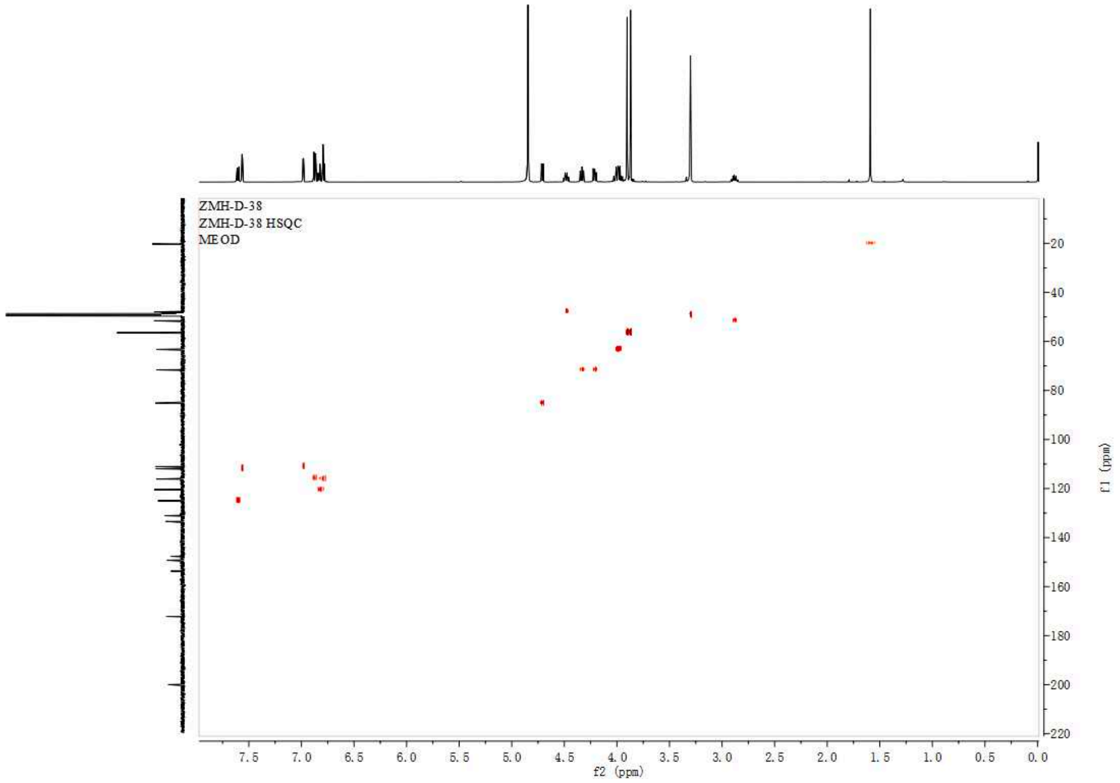

Figure S5. HSQC spectrum of **1**

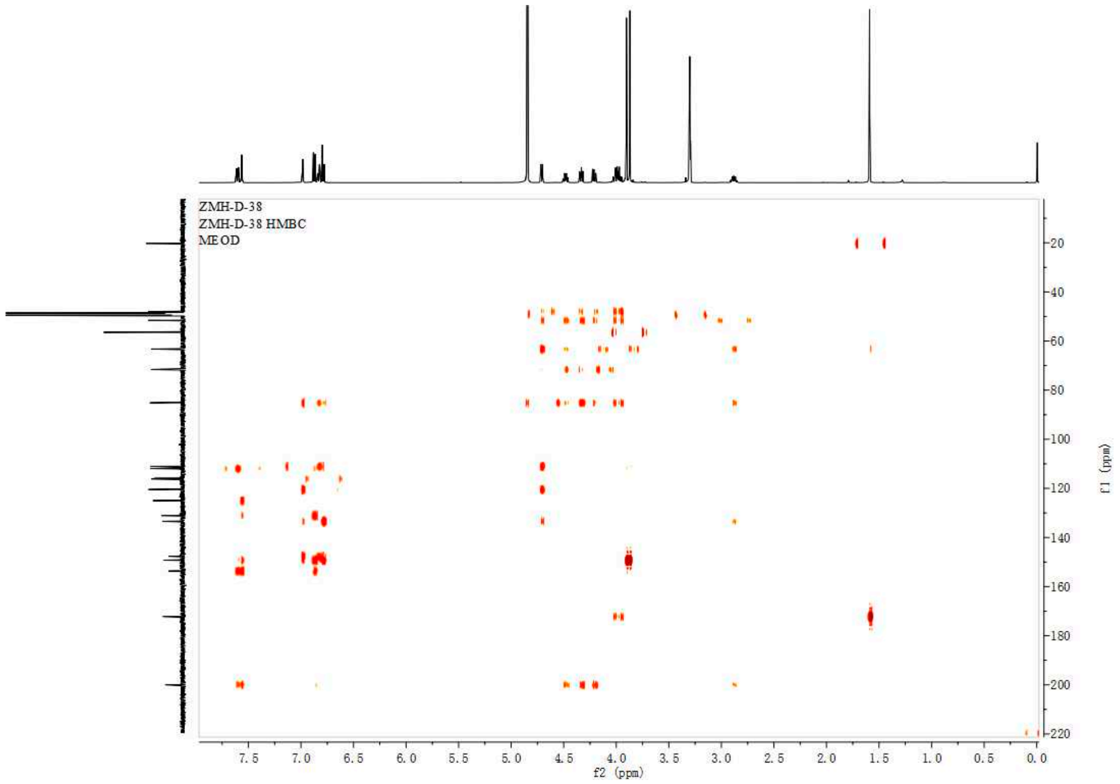

Figure S6. HMBC spectrum of **1**

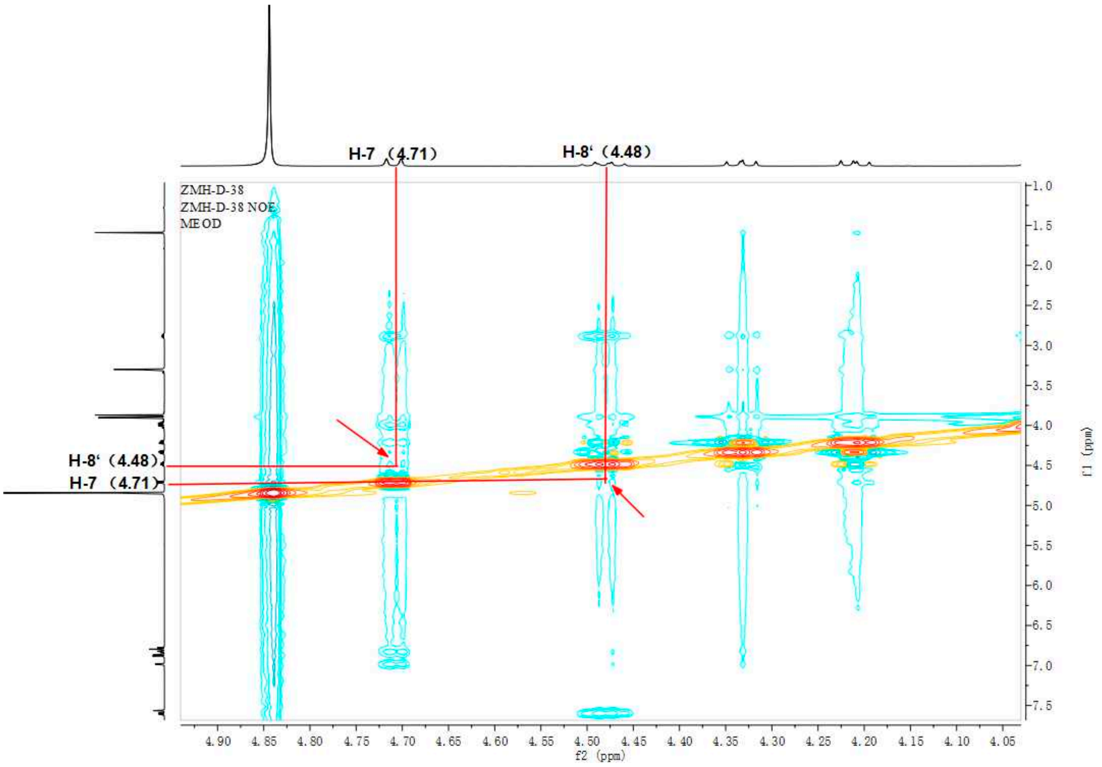

Figure S7. NOESY spectrum of **1**

Display Report

Analysis Info

Analysis Name	D:\Data\GJH\FXL\ZMH-D-38.d	Acquisition Date	2/10/2023 5:23:52 PM
Method	tune_pos_standard_20141031.m	Operator	Demo User
Sample Name	ZMH-D-38	Instrument	maXis HD 1820881.21303
Comment			

Acquisition Parameter

Source Type	ESI	Ion Polarity	Positive	Set Nebulizer	0.3 Bar
Focus	Active	Set Capillary	3500 V	Set Dry Heater	200 °C
Scan Begin	50 m/z	Set End Plate Offset	-500 V	Set Dry Gas	4.0 l/min
Scan End	3000 m/z	Set Charging Voltage	2000 V	Set Divert Valve	Waste
		Set Corona	0 nA	Set APCI Heater	0 °C

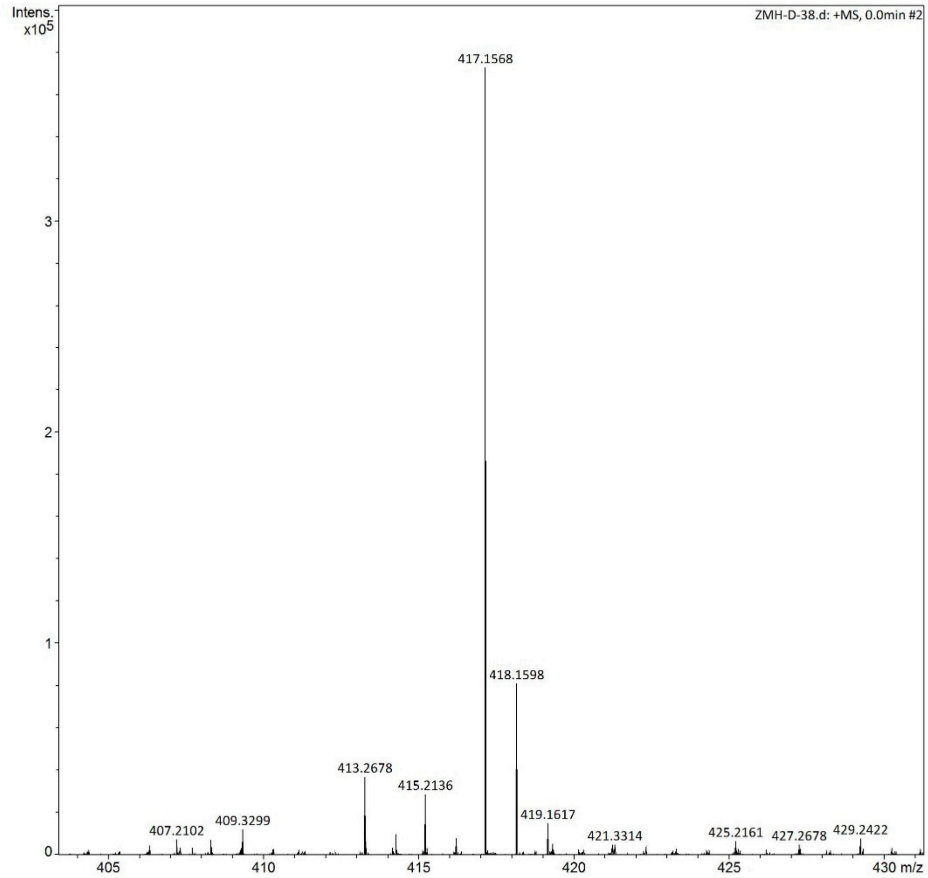

ZMH-D-38.d

Bruker Compass DataAnalysis 4.4 printed: 2/11/2023 4:39:42 PM by: demo Page 1 of 1

Figure S8. HR-ESI-MS spectrum of compound **1**

Thermo Scientific ~ VISIONpro SOFTWARE V4.41

Operator Name	(None Entered)	Date of Report	2023/2/25
Department	(None Entered)	Time of Report	18:12:37下午
Organization	(None Entered)		
Information	(None Entered)		

Scan Graph

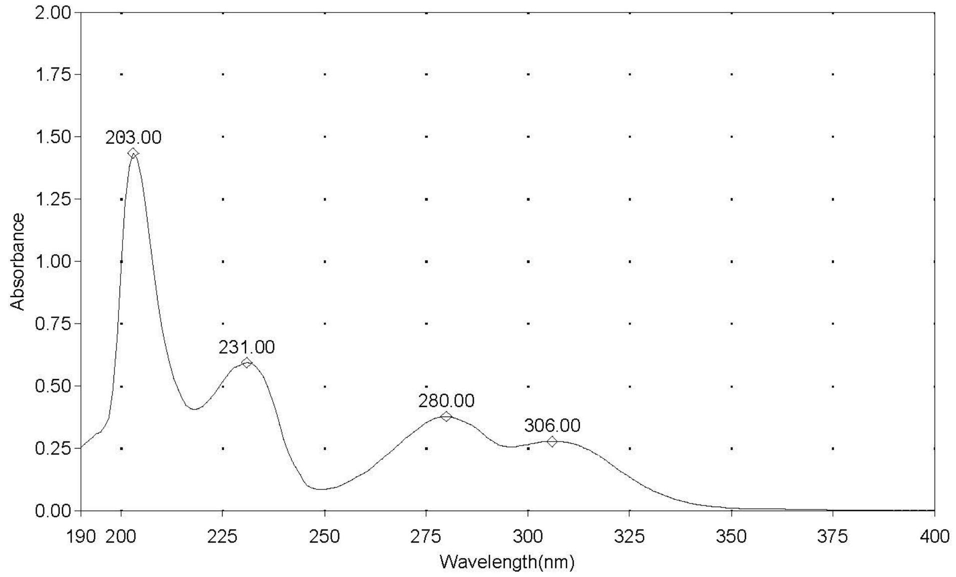

Results Table - scan007,ZMH-D-38,Cycle01

nm	A	**Peak Pick Method**
203.00	1.432	Find 8 Peaks Above -3.0000 A
231.00	.594	Start Wavelength 190.00 nm
280.00	.378	Stop Wavelength 400.00 nm
306.00	.278	Sort By Wavelength
Sensitivity	Medium	

Page 1, Scan Graph

Figure S9. UV spectrum of **1**

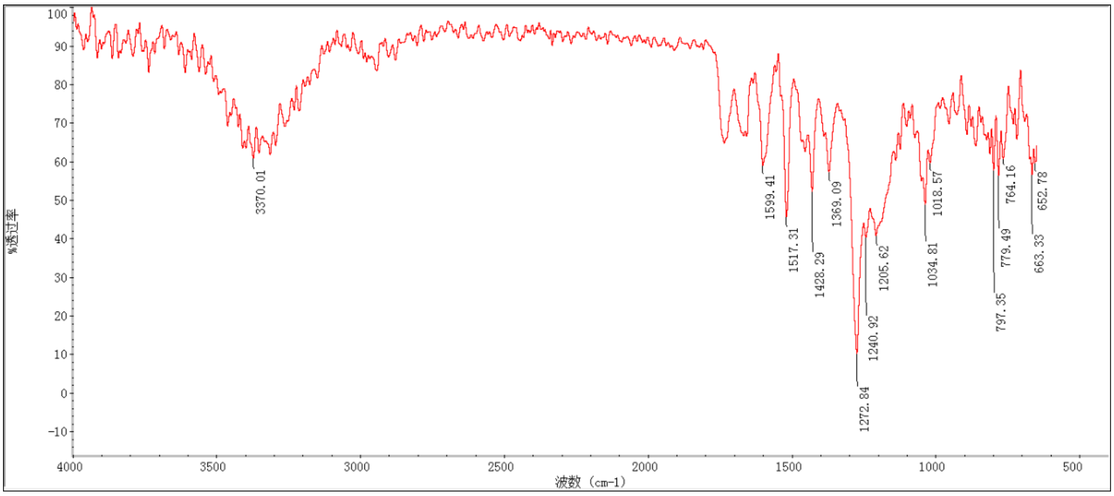

Figure S10. IR spectrum of **1**

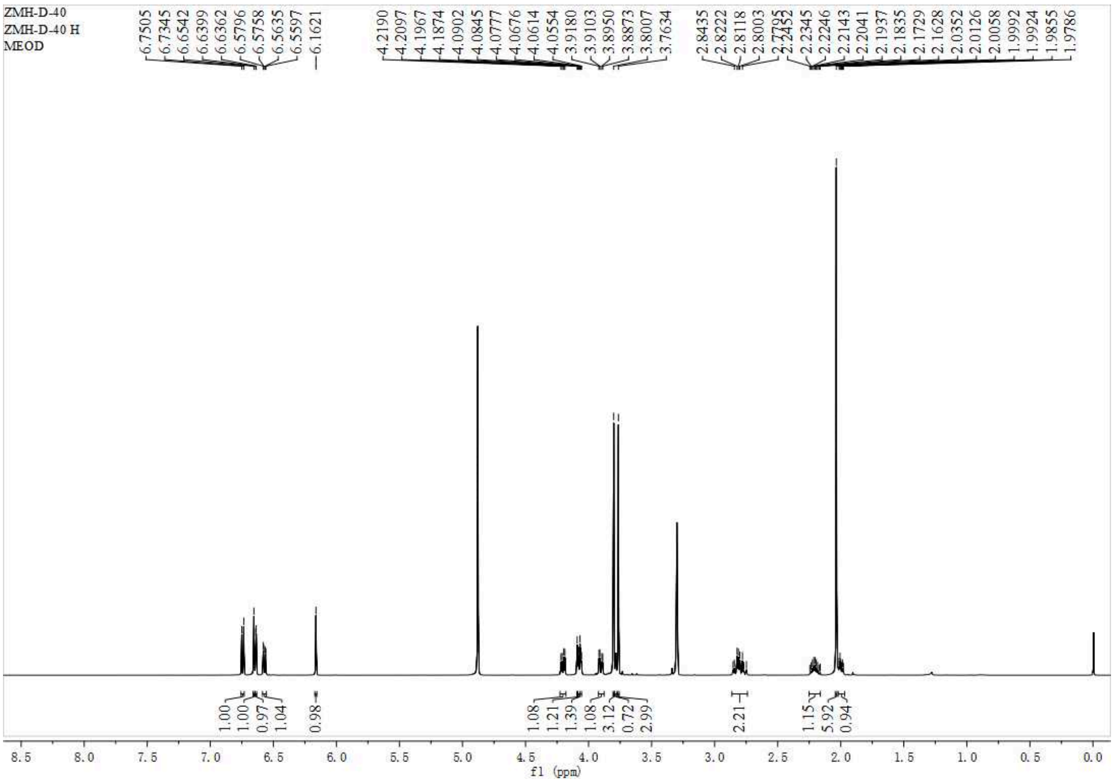

Figure S11. ^{1}H NMR spectrum (500MHz, CD_3OD) of **2**

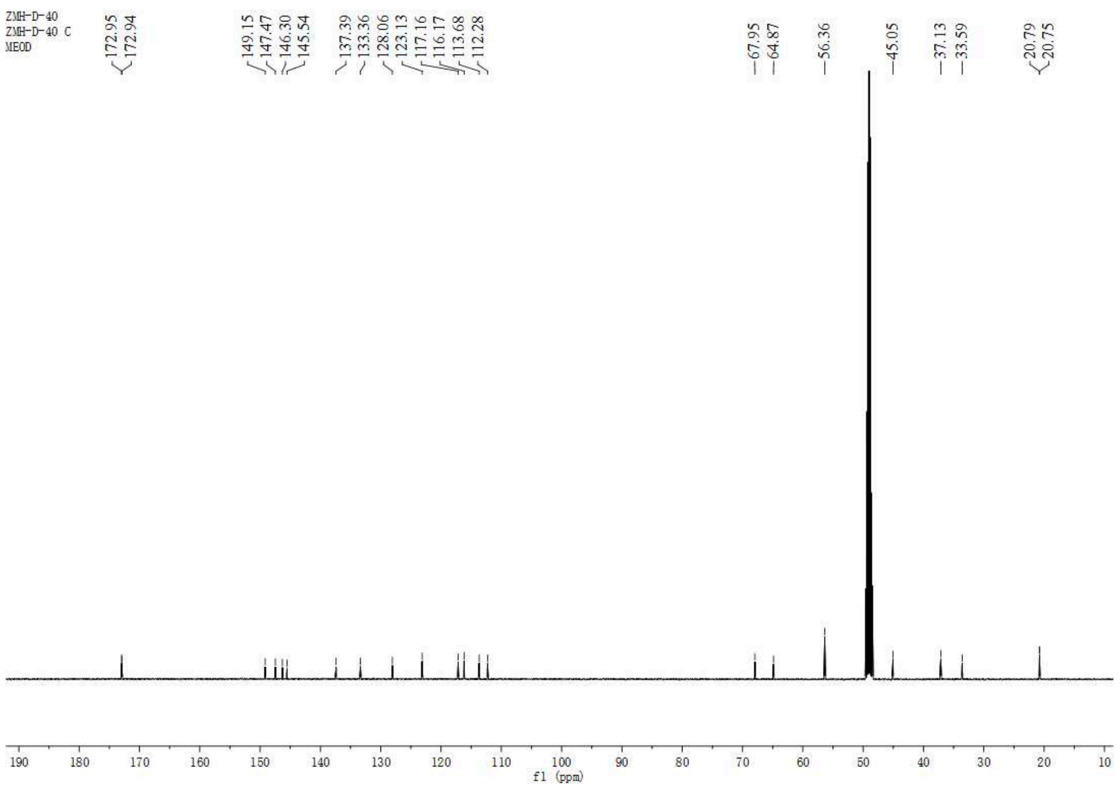

Figure S12. ^{13}C NMR spectrum (125MHz, CD_3OD) of **2**

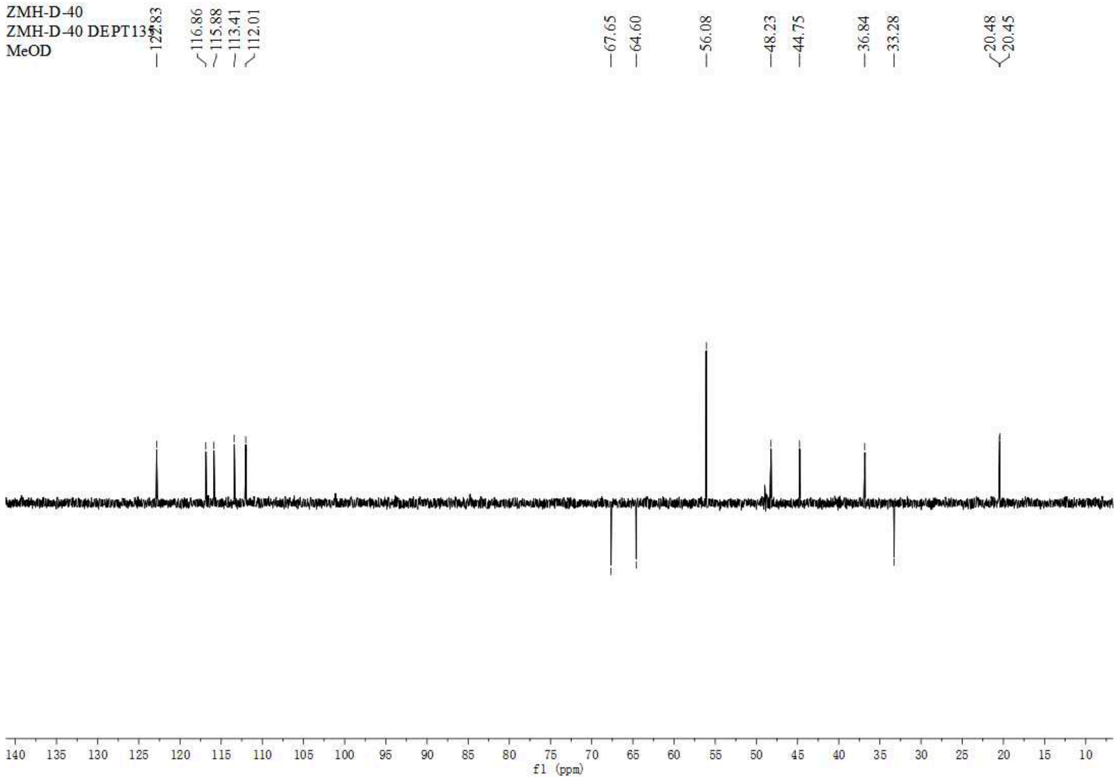

Figure S13. DEPT spectrum of **2**

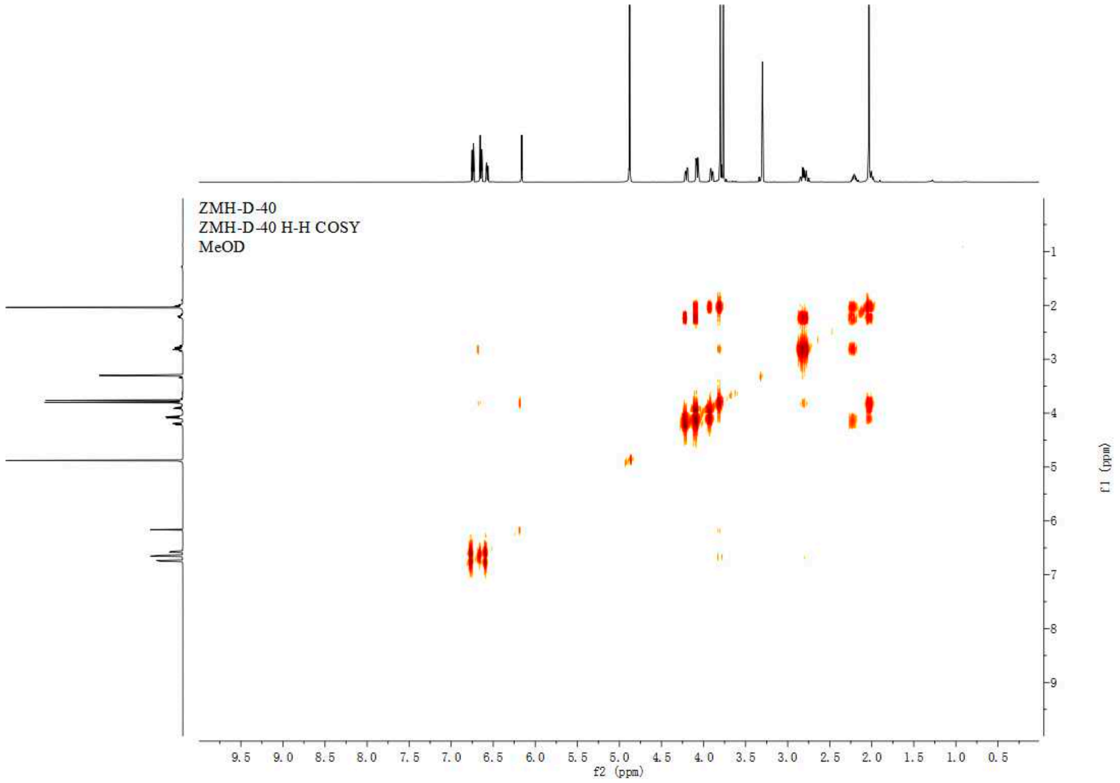

Figure S14. ^{1}H-^{1}H COSY spectrum of **2**

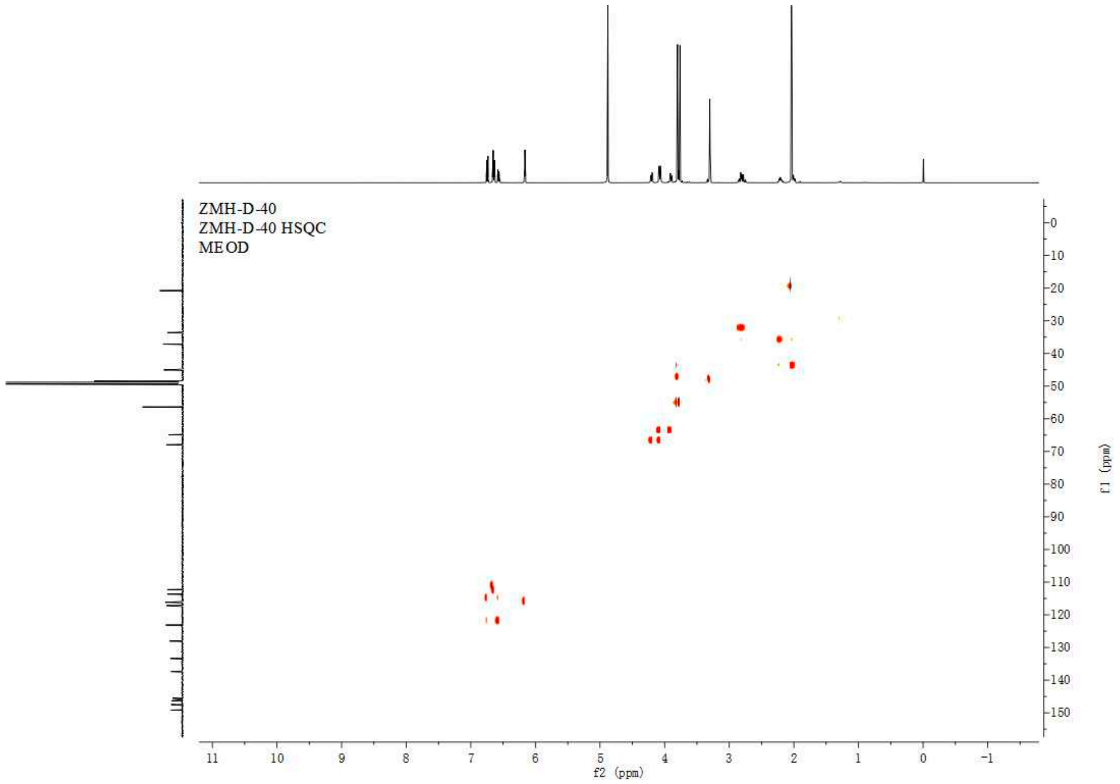

Figure S15. HSQC spectrum of **2**

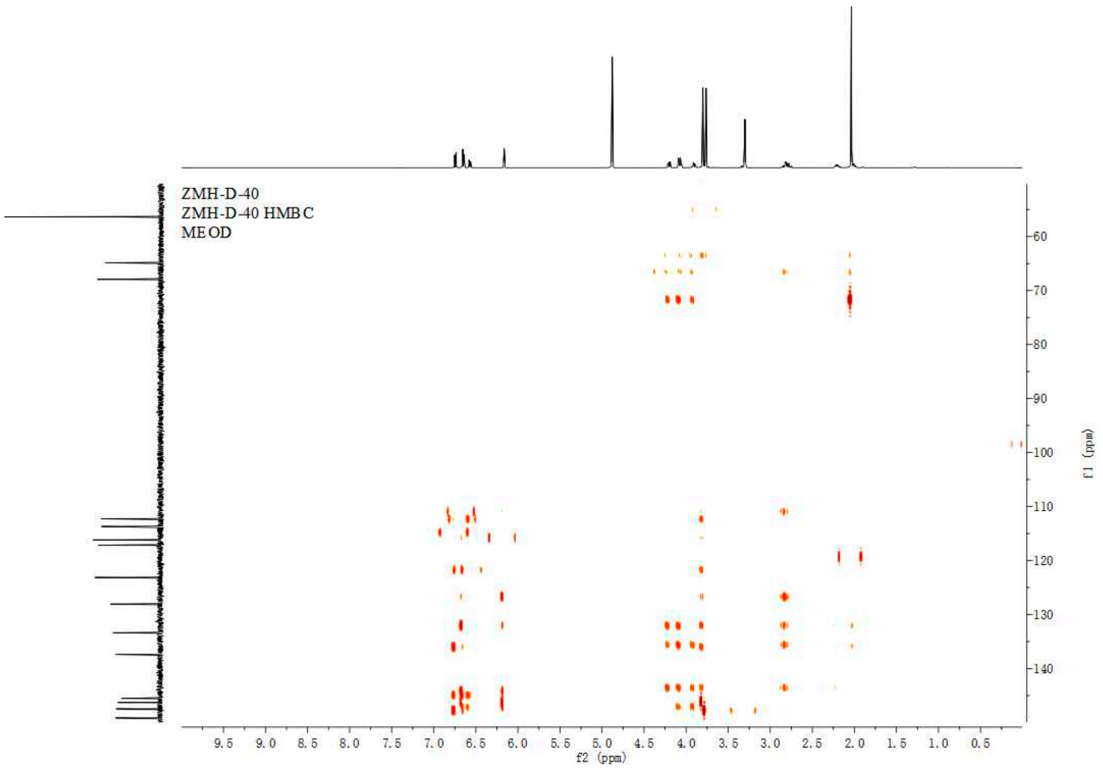

Figure S16. HMBC spectrum of **2**

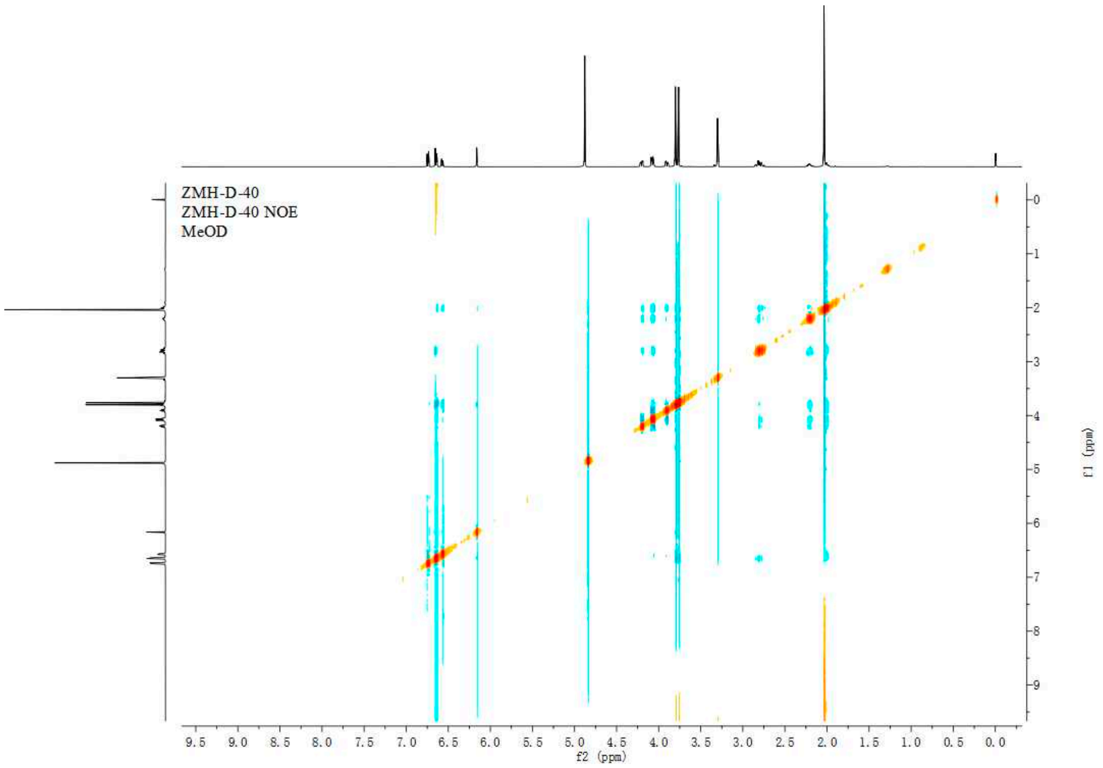

Figure S17. NOESY spectrum of **2**

Display Report

Analysis Info

Analysis Name	D:\Data\GJH\FXL\ZMH-D-40.d	Acquisition Date	2/10/2023 5:05:00 PM
Method	tune_pos_standard_20141031.m	Operator	Demo User
Sample Name	ZMH-D-40	Instrument	maXis HD 1820881.21303
Comment			

Acquisition Parameter

Source Type	ESI	Ion Polarity	Positive	Set Nebulizer	0.3 Bar
Focus	Active	Set Capillary	3500 V	Set Dry Heater	200 °C
Scan Begin	50 m/z	Set End Plate Offset	-500 V	Set Dry Gas	4.0 l/min
Scan End	3000 m/z	Set Charging Voltage	2000 V	Set Divert Valve	Waste
		Set Corona	0 nA	Set APCI Heater	0 °C

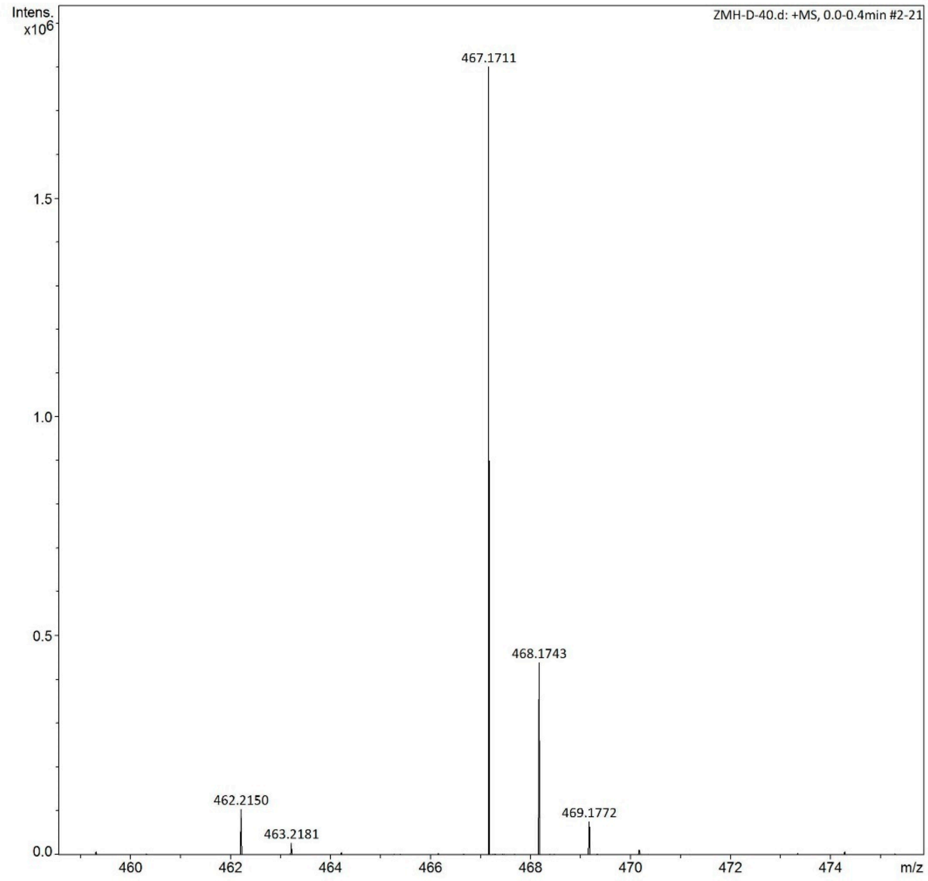

ZMH-D-40.d
Bruker Compass DataAnalysis 4.4 printed: 2/11/2023 4:57:44 PM by: demo Page 1 of 1

Figure S18. HR-ESI-MS spectrum of compound **2**

Thermo Scientific ~ VISIONpro SOFTWARE V4.41

Operator Name	(None Entered)	Date of Report	2023/2/25
Department	(None Entered)	Time of Report	17:58:50下午
Organization	(None Entered)		
Information	(None Entered)		

Scan Graph

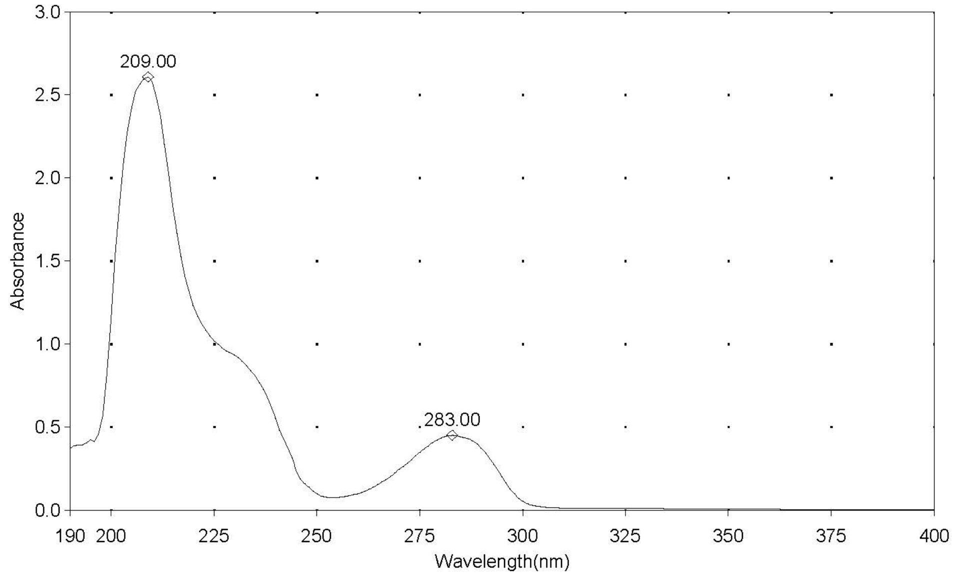

Results Table - scan005,ZMH-D-40,Cycle01

nm	A	**Peak Pick Method**
209.00	2.607	Find 8 Peaks Above -3.0000 A
283.00	.452	Start Wavelength 190.00 nm
		Stop Wavelength 400.00 nm
		Sort By Wavelength
Sensitivity	Medium	

Page 1, Scan Graph

Figure S19. UV spectrum of **2**

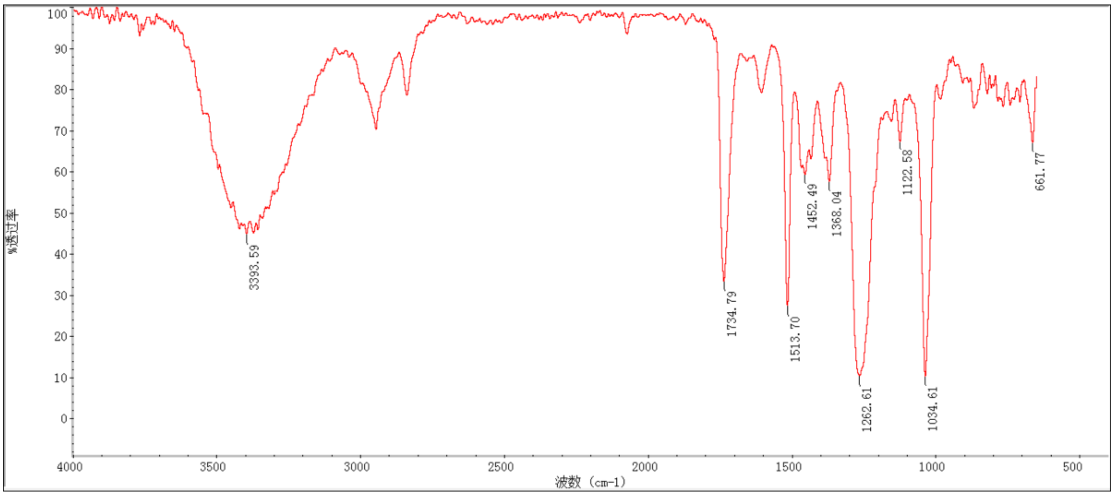

Figure S20. IR spectrum of **2**

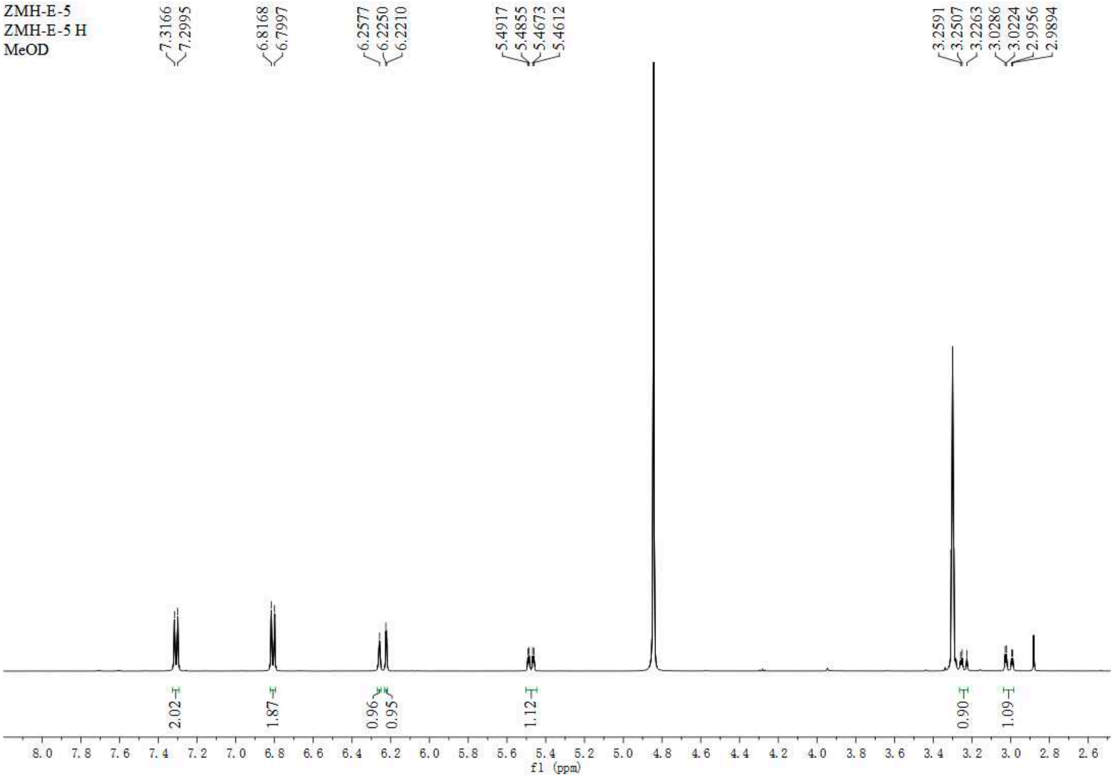

Figure S21. ^{1}H NMR spectrum (500MHz, CD_3OD) of **3**

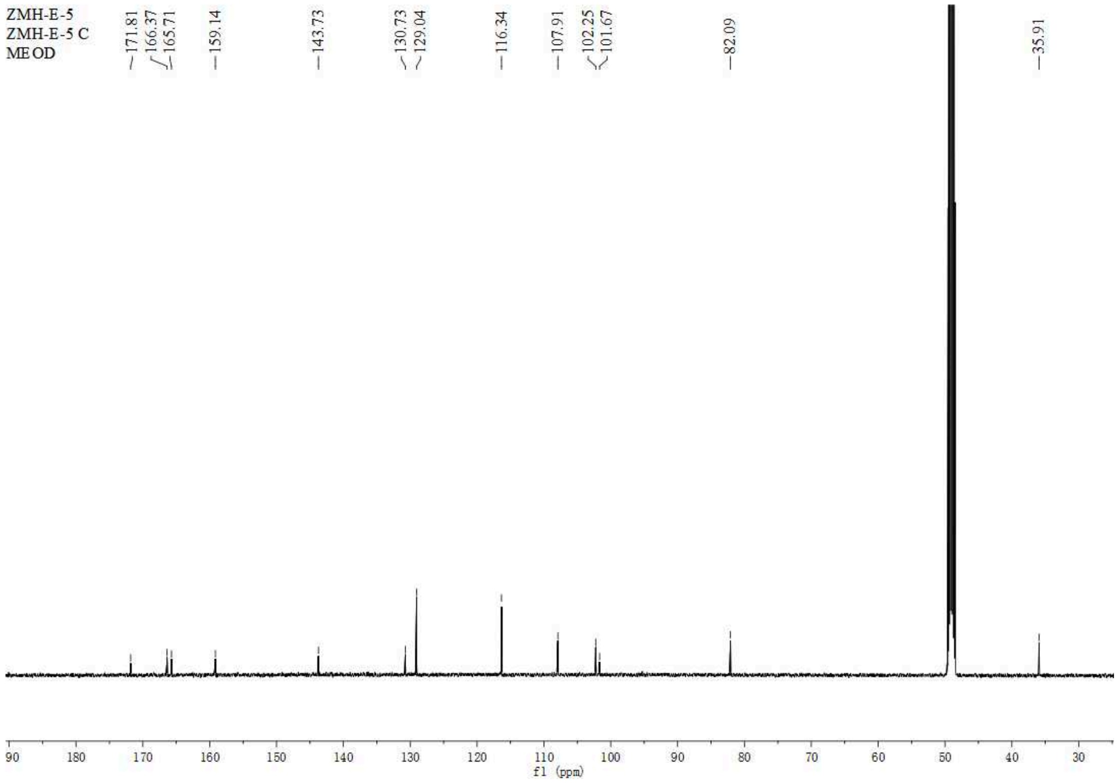

Figure S22. ^{13}C NMR spectrum (125MHz, CD_3OD) of **3**

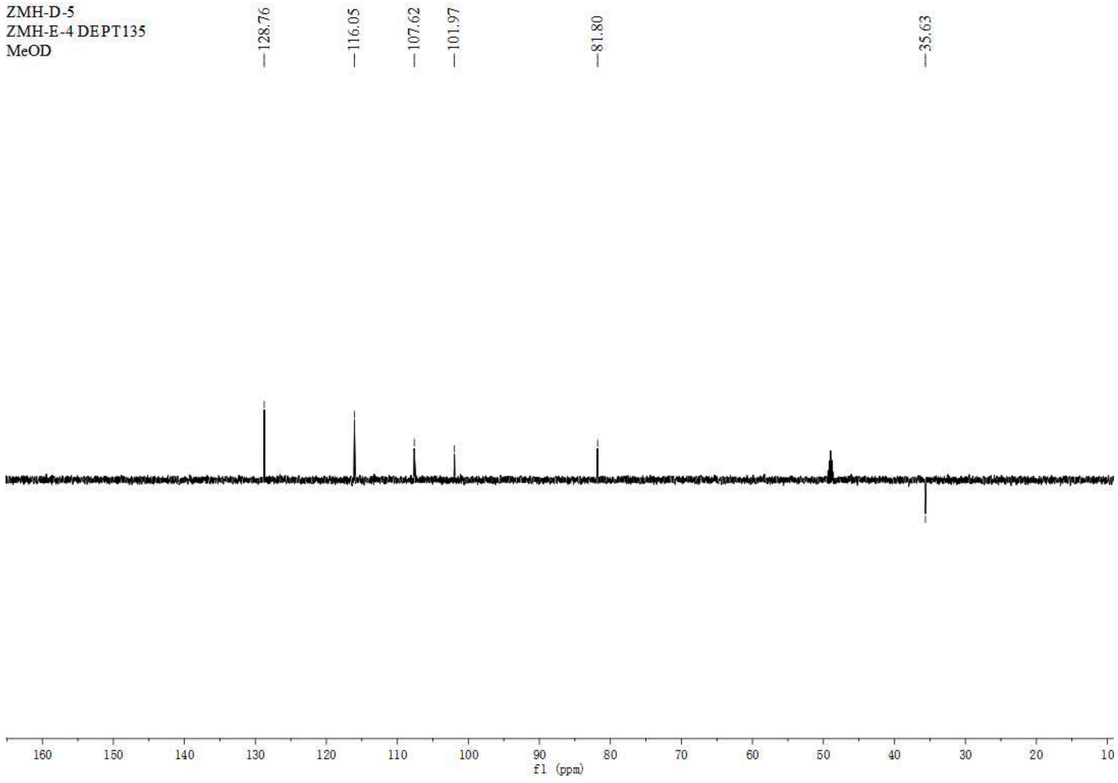

Figure S23. DEPT spectrum of **3**

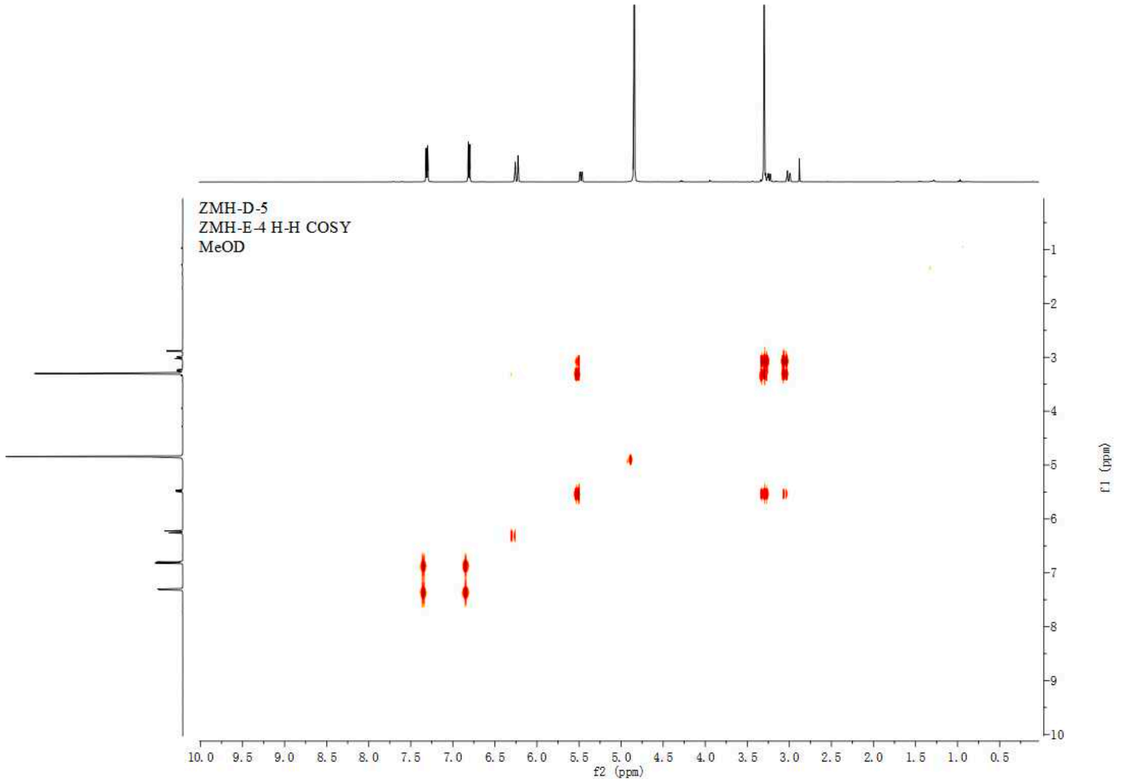

Figure S24. ^{1}H-^{1}H COSY spectrum of **4**

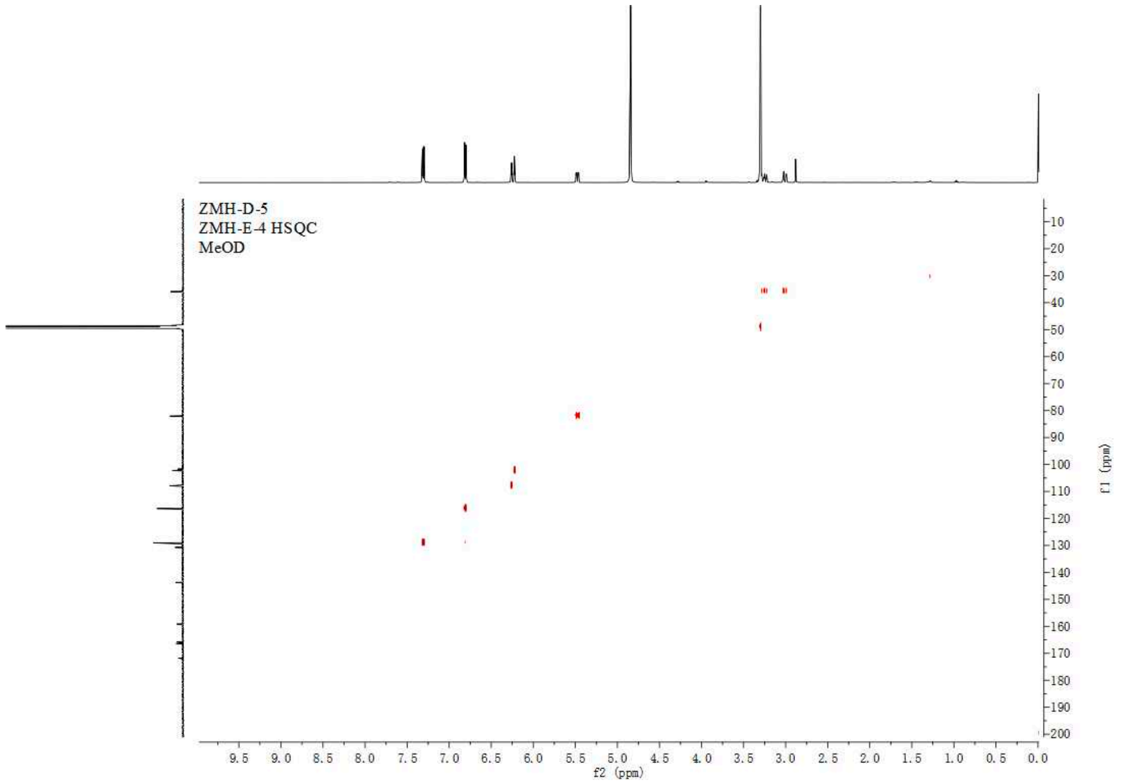

Figure S25. HSQC spectrum of **3**

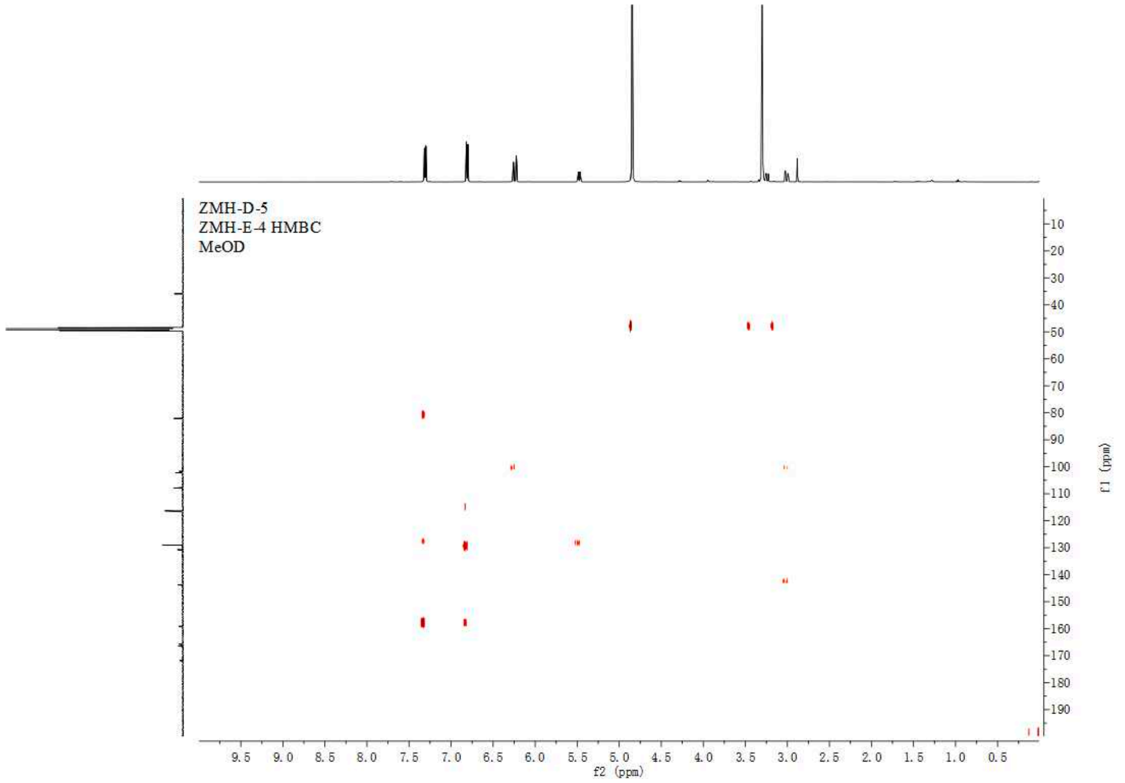

Figure S26. HMBC spectrum of **3**

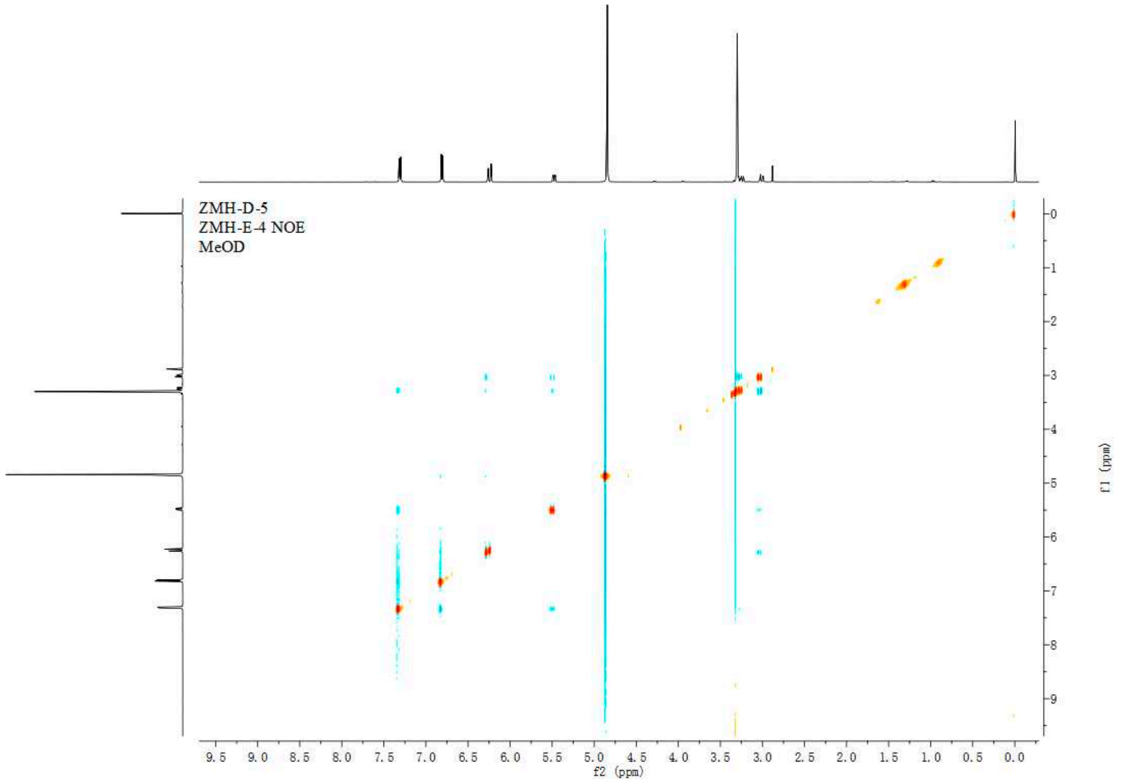

Figure S27. NOESY spectrum of **3**

Display Report

Analysis Info

Analysis Name	D:\Data\GJH\FXL\ZMH-E-5.d	Acquisition Date	2/10/2023 9:33:06 PM
Method	tune_pos_standard_20141031.m	Operator	Demo User
Sample Name	ZMH-E-5	Instrument	maXis HD 1820881.21303
Comment			

Acquisition Parameter

Source Type	ESI	Ion Polarity	Positive	Set Nebulizer	0.3 Bar
Focus	Active	Set Capillary	3500 V	Set Dry Heater	200 °C
Scan Begin	50 m/z	Set End Plate Offset	-500 V	Set Dry Gas	4.0 l/min
Scan End	3000 m/z	Set Charging Voltage	2000 V	Set Divert Valve	Waste
		Set Corona	0 nA	Set APCI Heater	0 °C

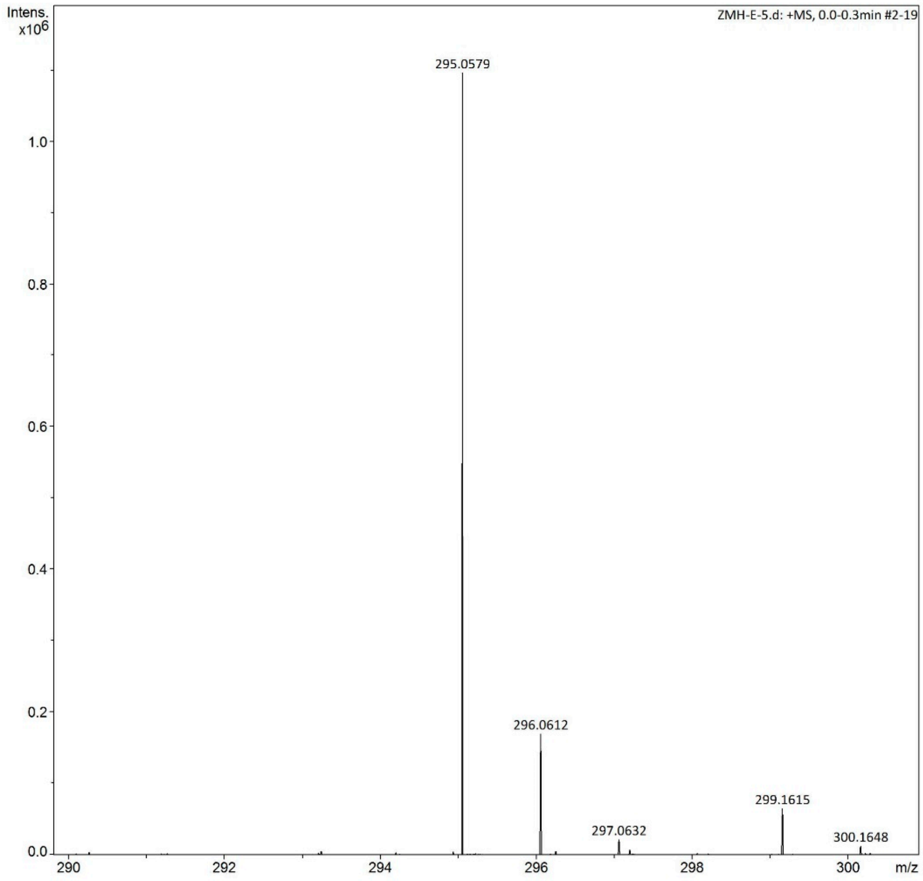

ZMH-E-5.d

Bruker Compass DataAnalysis 4.4 printed: 2/11/2023 3:48:51 PM by: demo Page 1 of 1

Figure S28. HR-ESI-MS spectrum of compound **3**

Thermo Scientific ~ VISIONpro SOFTWARE V4.41

Operator Name	(None Entered)	Date of Report	2023/2/25
Department	(None Entered)	Time of Report	18:18:10下午
Organization	(None Entered)		
Information	(None Entered)		

Scan Graph

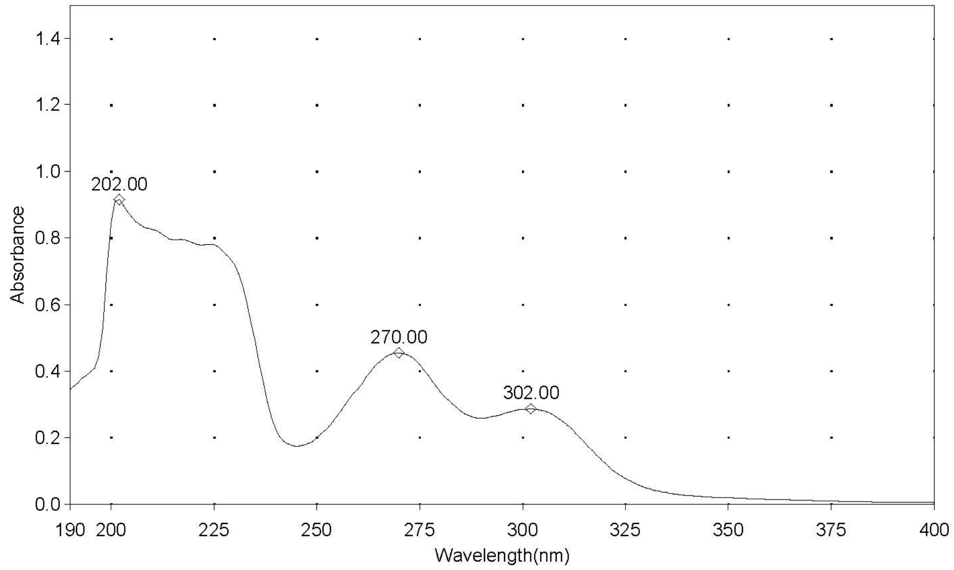

Results Table - scan008,ZMH-E-5,Cycle01

nm	A	**Peak Pick Method**
202.00	.915	Find 8 Peaks Above -3.0000 A
270.00	.455	Start Wavelength 190.00 nm
302.00	.286	Stop Wavelength 400.00 nm
		Sort By Wavelength
Sensitivity	Medium	

Page 1, Scan Graph

Figure S29. UV spectrum of **3**

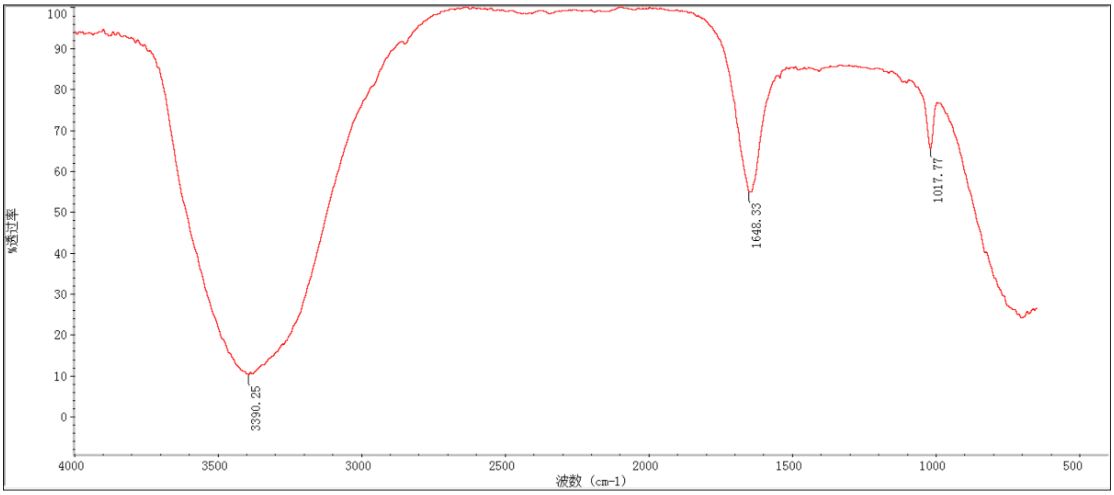

Figure S30. IR spectrum of **3**

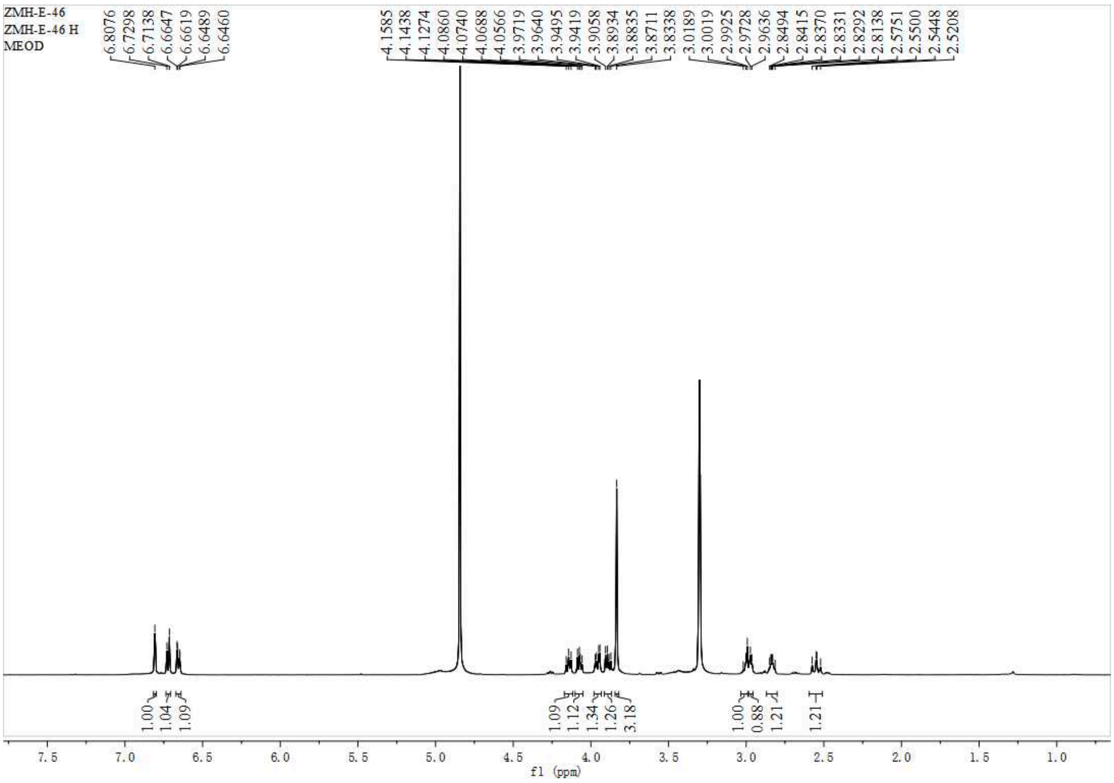

Figure S31. ^{1}H NMR spectrum (500MHz, CD_3OD) of **4**

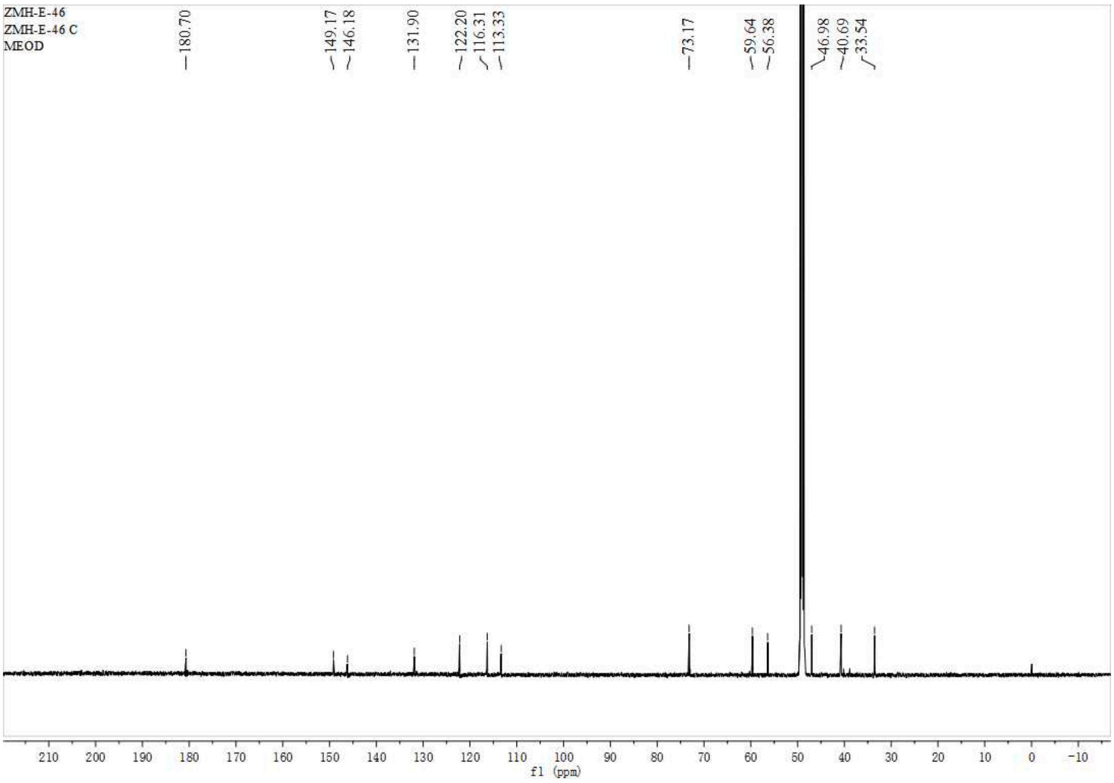

Figure S32. ^{13}C NMR spectrum (125MHz, CD_3OD) of **4**

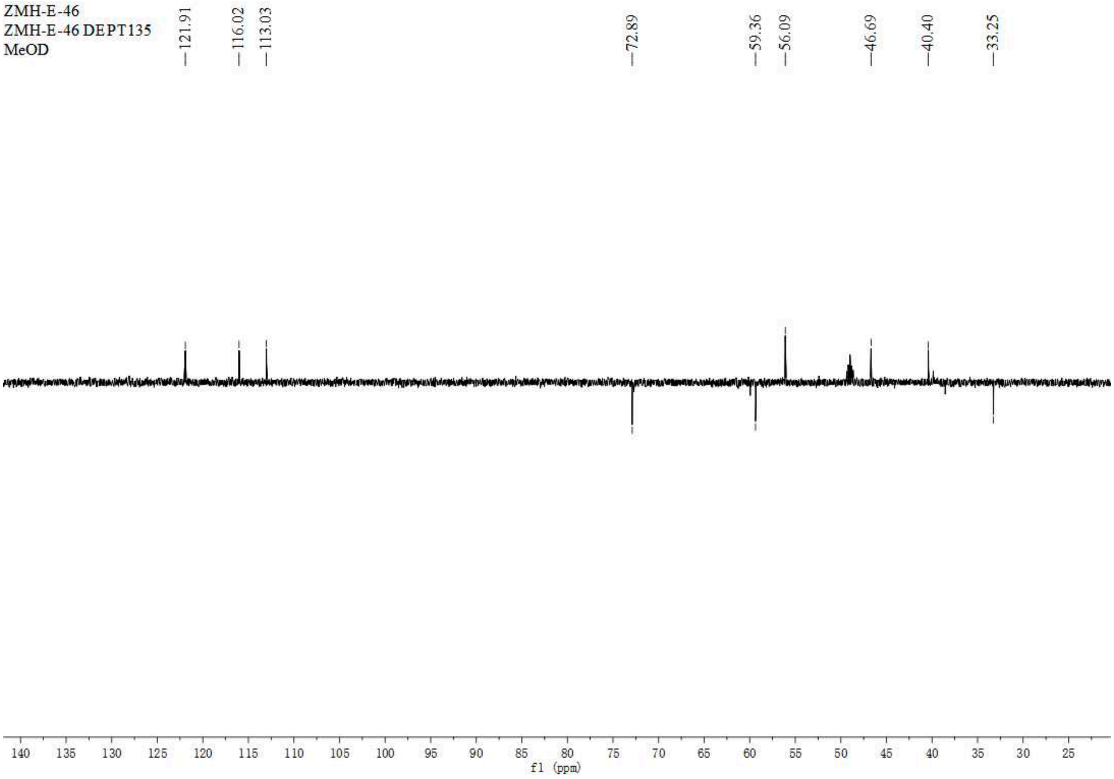

Figure S33. DEPT spectrum of **4**

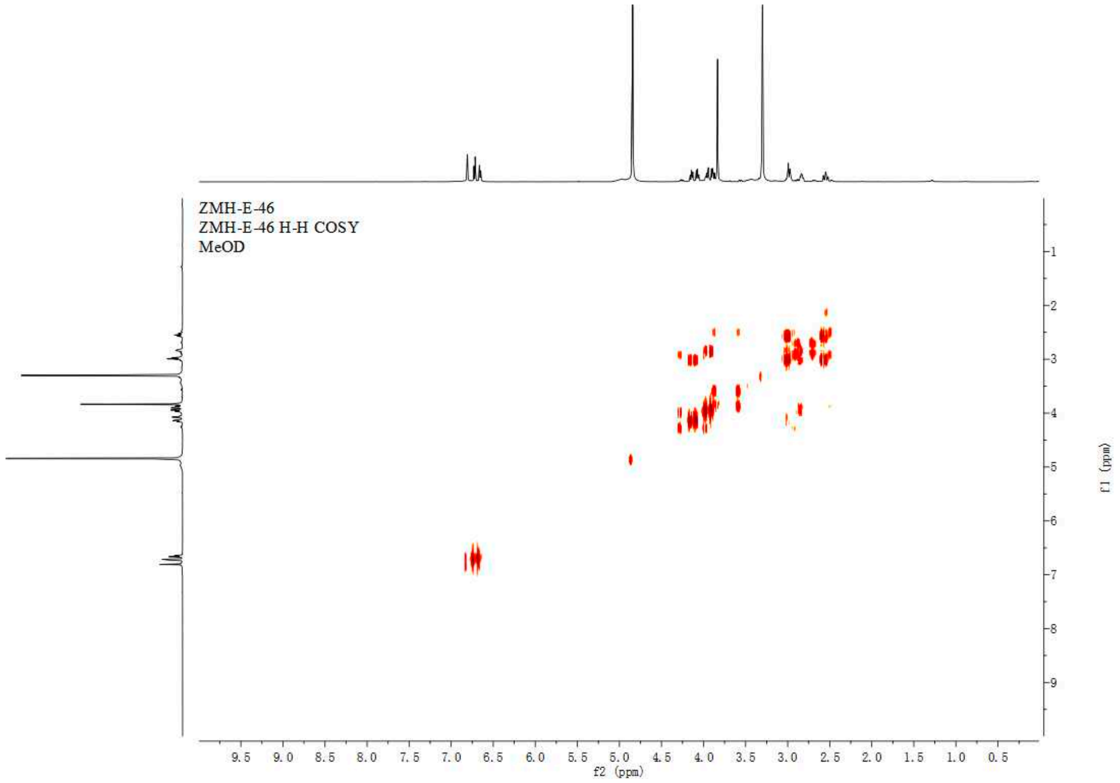

Figure S34. ^{1}H-^{1}H COSY spectrum of **4**

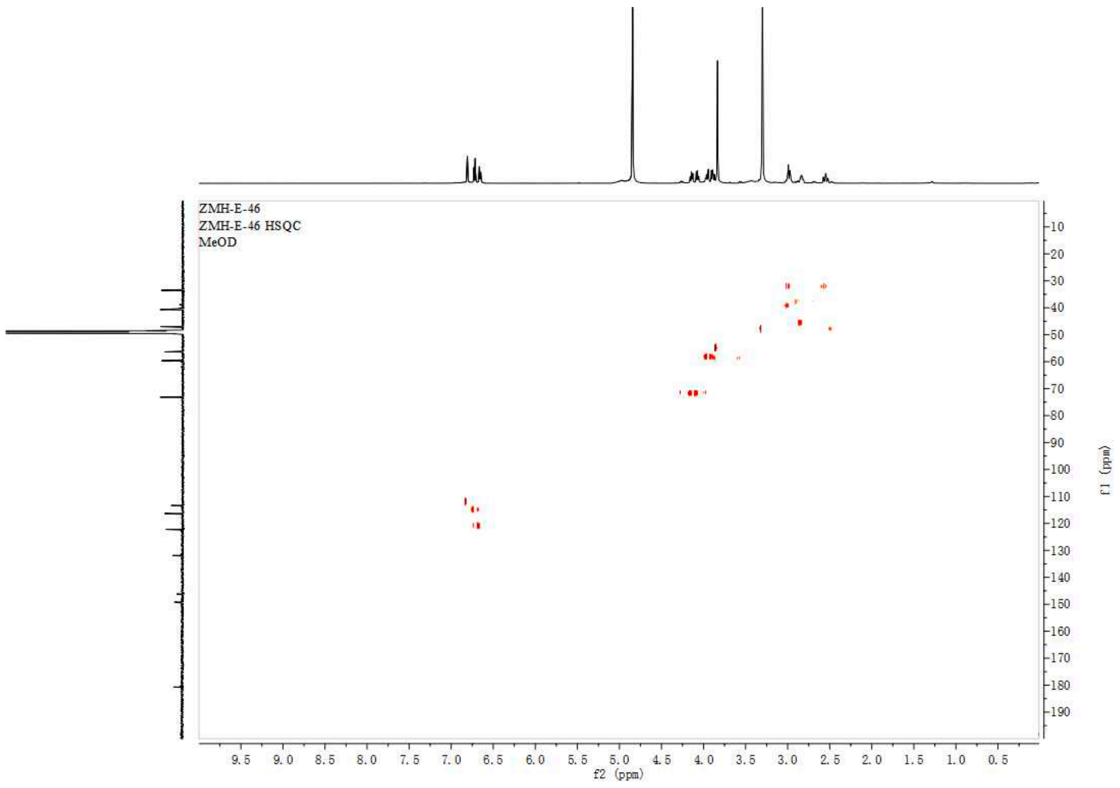

Figure S35. HSQC spectrum of **4**

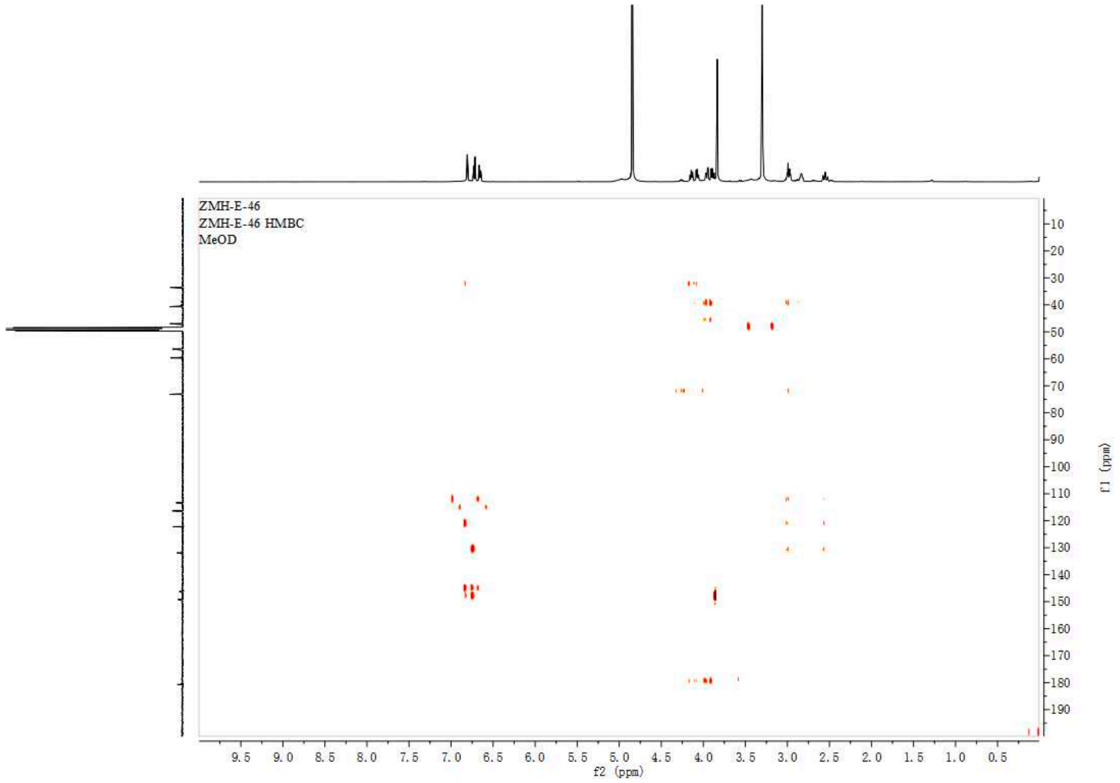

Figure S36. HMBC spectrum of **4**

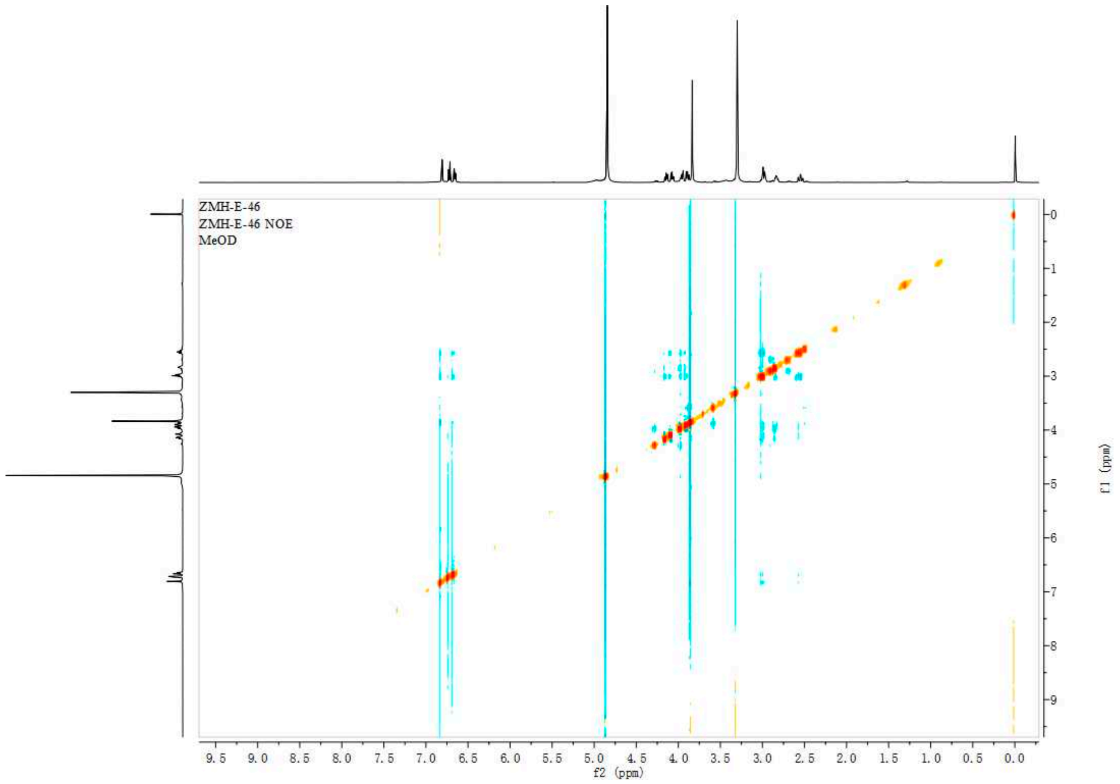

Figure S37. NOESY spectrum of **4**

Display Report

Analysis Info		Acquisition D	2023/2/10 17:09:38
Analysis Name	H:\20230221GJH\FXL\ZMH-E-46.d		
Method	tune_pos_standard_20141031.m	Operator	Demo User
Sample Name	ZMH-E-46	Instrumen	maXis HD 1820881.21303
Comment			

Acquisition Paramet

Source Type	ESI	Ion Polarity	Positive	Set Nebulizer	0.3 Bar
Focus	Active	Set Capillary	3500 V	Set Dry Heater	200 °C
Scan Begin	50 m/z	Set End Plate Offset	-500 V	Set Dry Gas	4.0 l/min
Scan End	3000 m/z	Set Charging Voltage	2000 V	Set Divert Valve	Waste
		Set Corona	0 nA	Set APCI Heater	0 °C

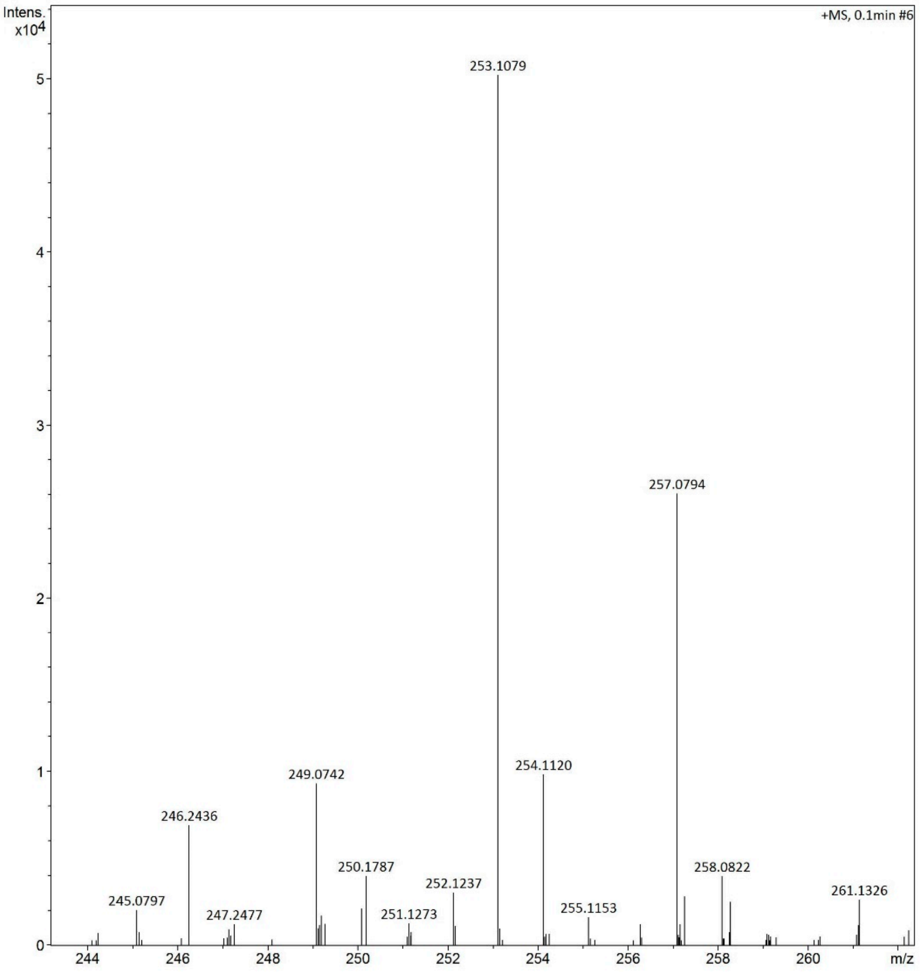

ZMH-E-46.d

4000031FC01153

Figure S38. HR-ESI-MS spectrum of compound **4**

Thermo Scientific ~ VISIONpro SOFTWARE V4.41

Operator Name	(None Entered)	Date of Report	2023/2/25
Department	(None Entered)	Time of Report	18:29:43下午
Organization	(None Entered)		
Information	(None Entered)		

Scan Graph

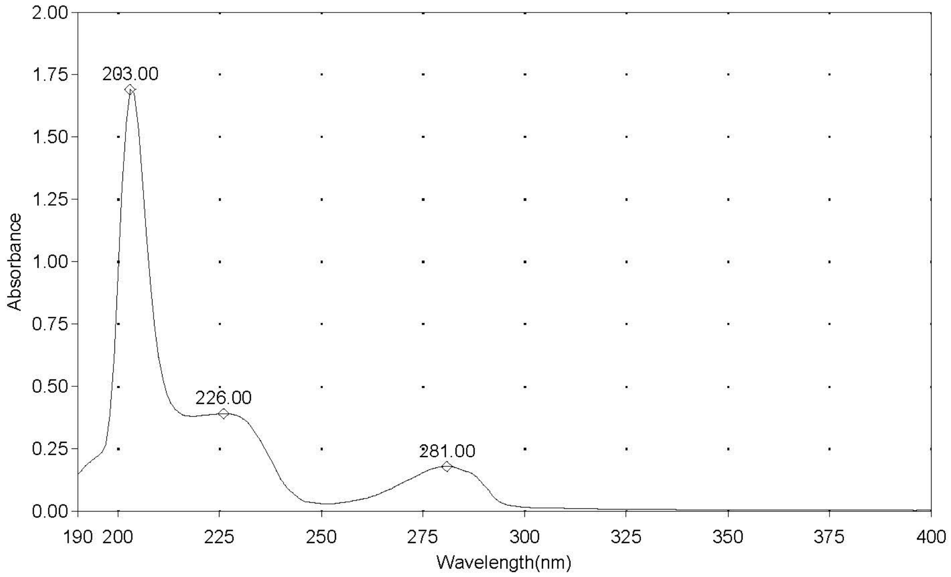

Results Table - scan011,ZMH-E-46,Cycle01

nm	A	**Peak Pick Method**
203.00	1.690	Find 8 Peaks Above -3.0000 A
226.00	.392	Start Wavelength 190.00 nm
281.00	.178	Stop Wavelength 400.00 nm
		Sort By Wavelength
Sensitivity	Medium	

Page 1, Scan Graph

Figure S39. UV spectrum of **4**

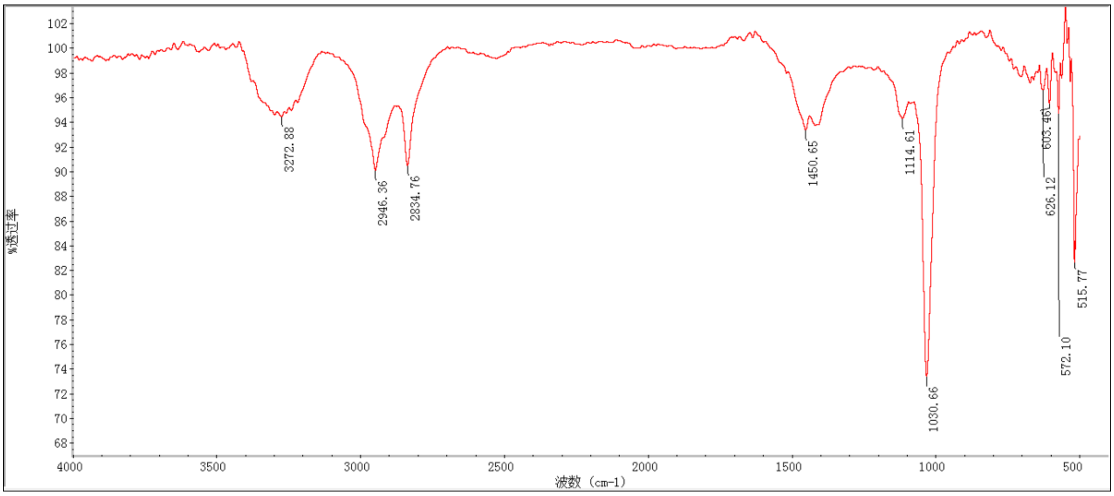

Figure S40. IR spectrum of **4**

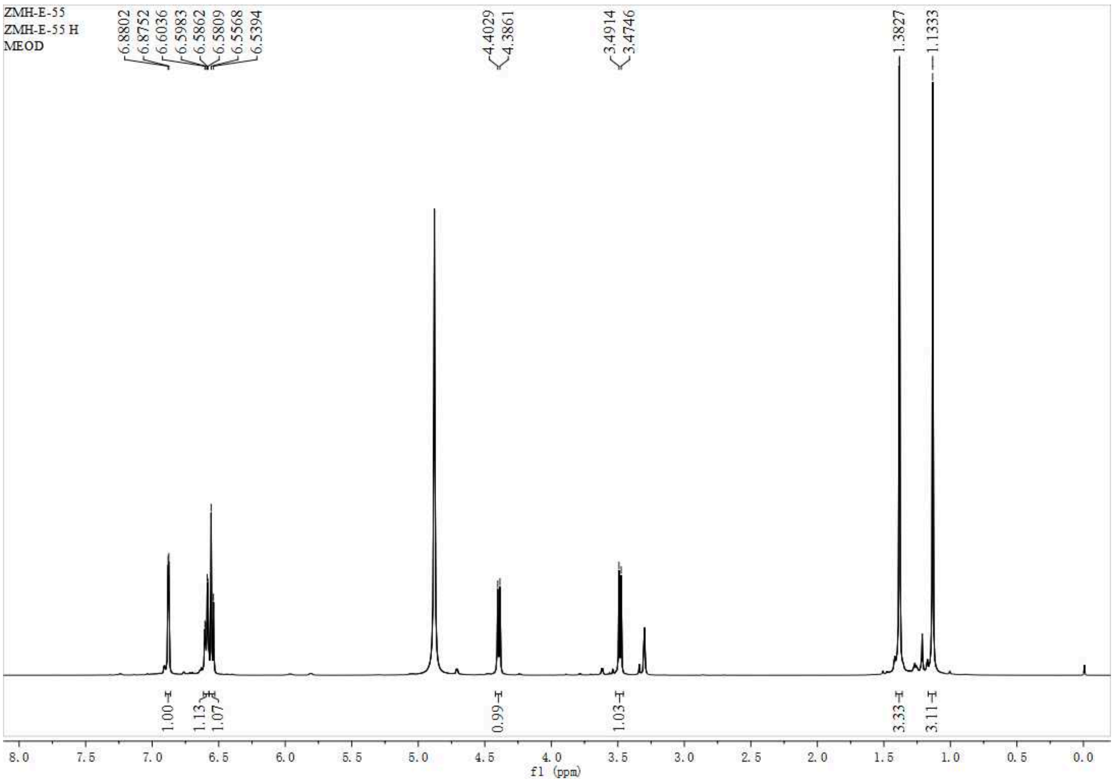

Figure S41. ^{1}H NMR spectrum (500MHz, CD_3OD) of **5**

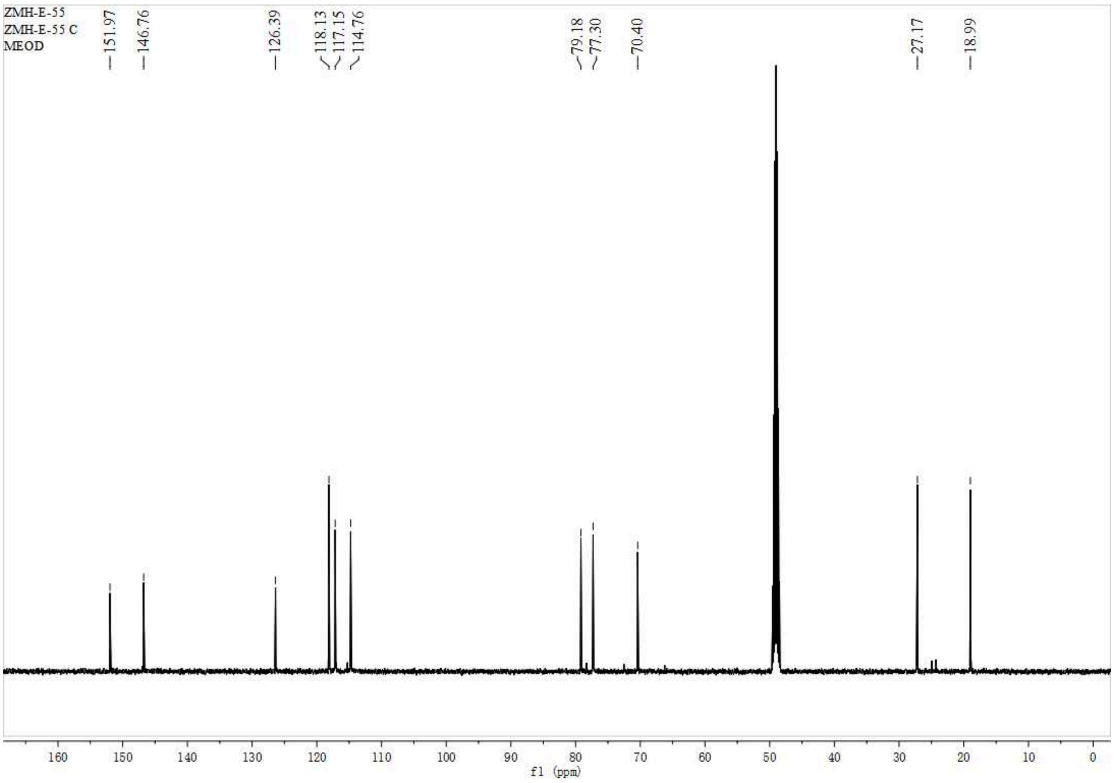

Figure S42. ^{13}C NMR spectrum (125MHz, CD_3OD) of **5**

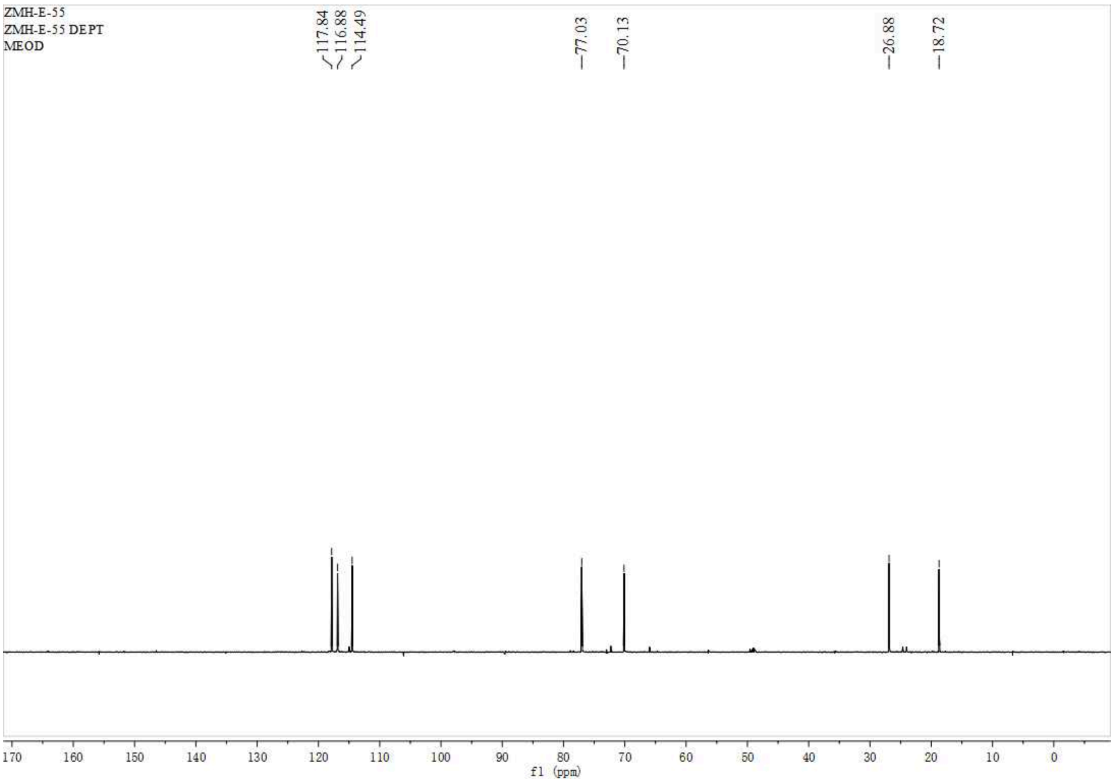

Figure S43. DEPT spectrum of **5**

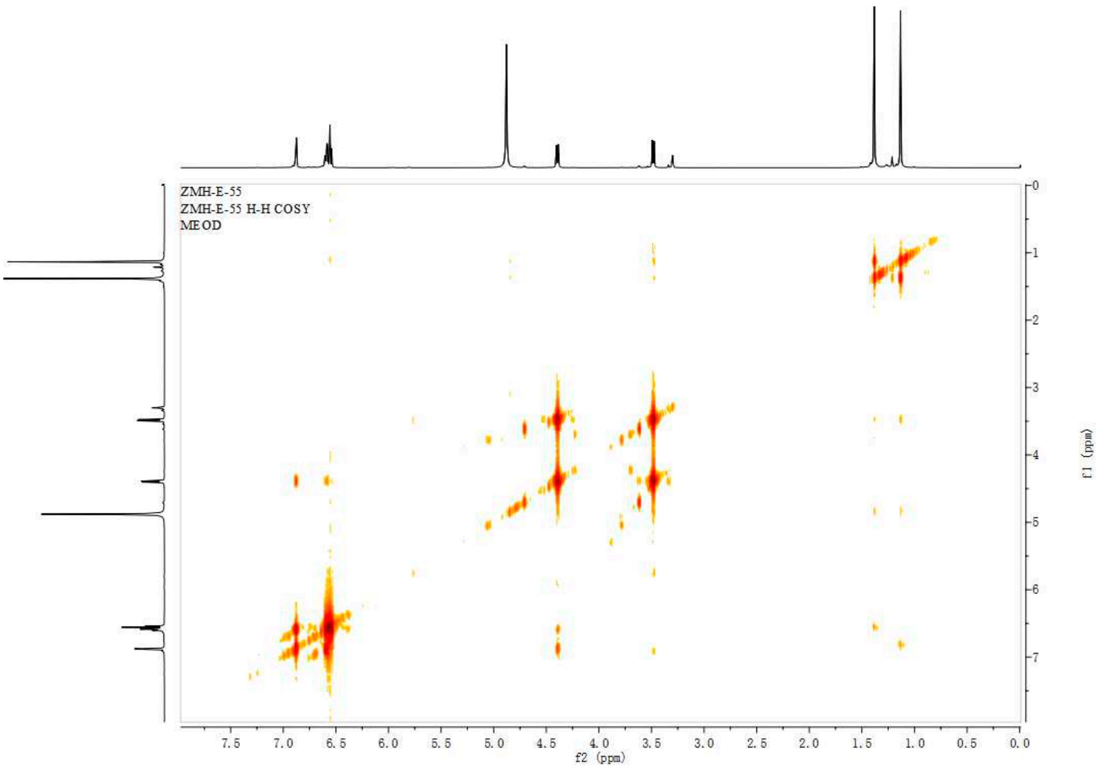

Figure S44. ^{1}H-^{1}H COSY spectrum of **5**

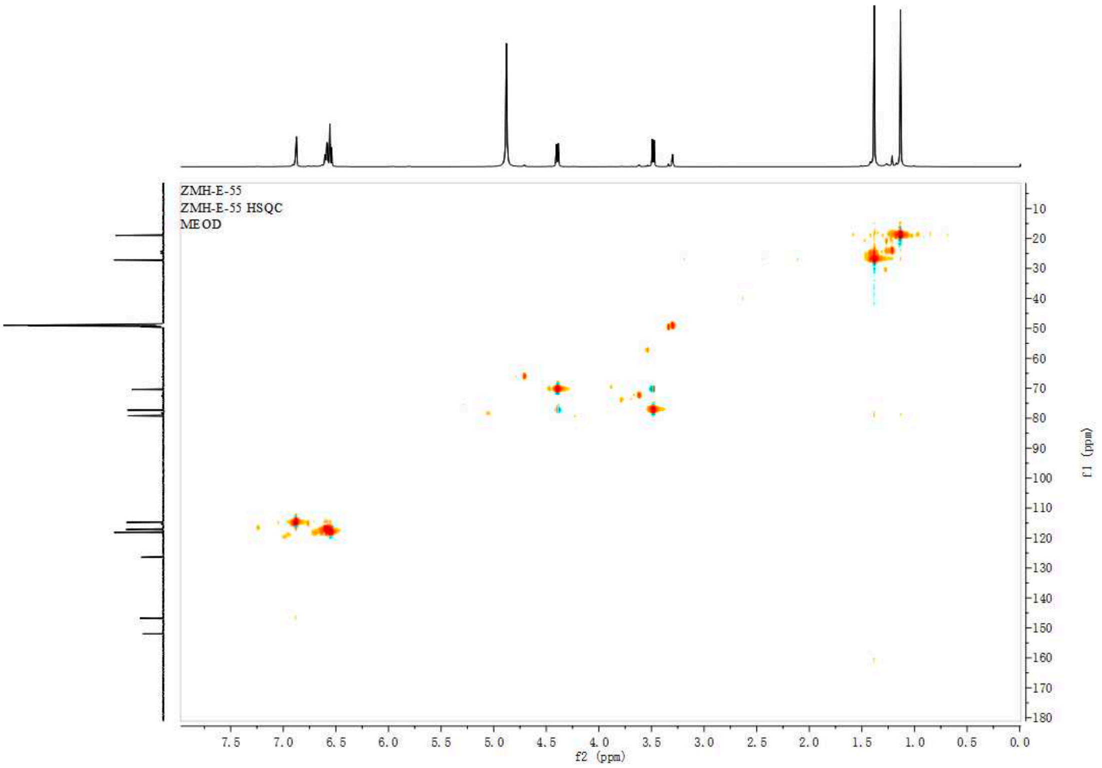

Figure S45. HSQC spectrum of **5**

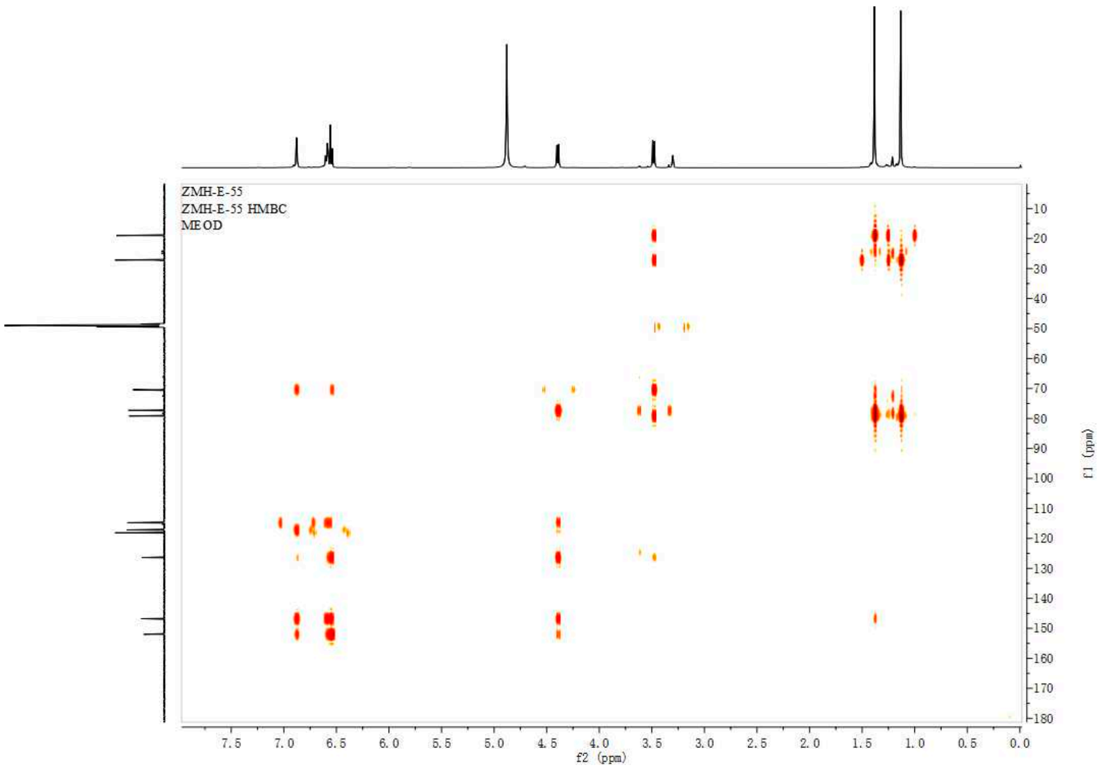

Figure S46. HMBC spectrum of **5**

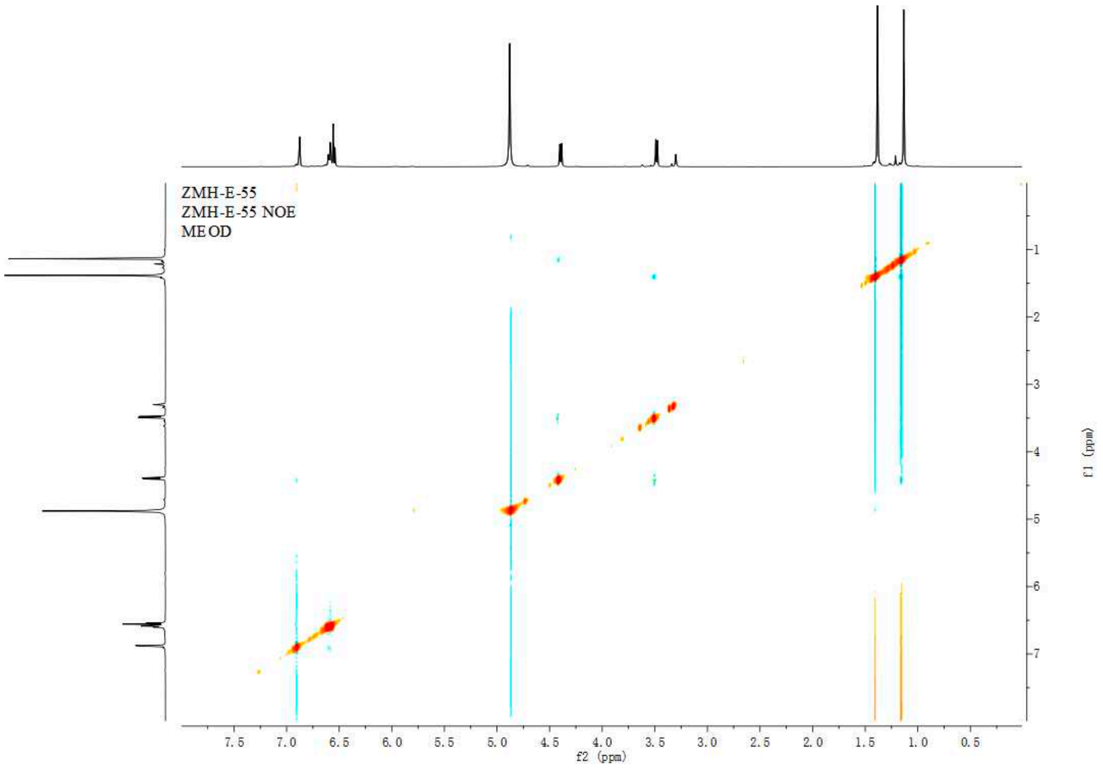

Figure S47. NOESY spectrum of **5**

Display Report

Analysis Info

Analysis Name	D:\Data\GJH\FXL\ZMH-E-55.d	Acquisition Date	2/10/2023 5:13:30 PM
Method	tune_pos_standard_20141031.m	Operator	Demo User
Sample Name	ZMH-E-55	Instrument	maXis HD 1820881.21303
Comment			

Acquisition Parameter

Source Type	ESI	Ion Polarity	Positive	Set Nebulizer	0.3 Bar
Focus	Active	Set Capillary	3500 V	Set Dry Heater	200 °C
Scan Begin	50 m/z	Set End Plate Offset	-500 V	Set Dry Gas	4.0 l/min
Scan End	3000 m/z	Set Charging Voltage	2000 V	Set Divert Valve	Waste
		Set Corona	0 nA	Set APCI Heater	0 °C

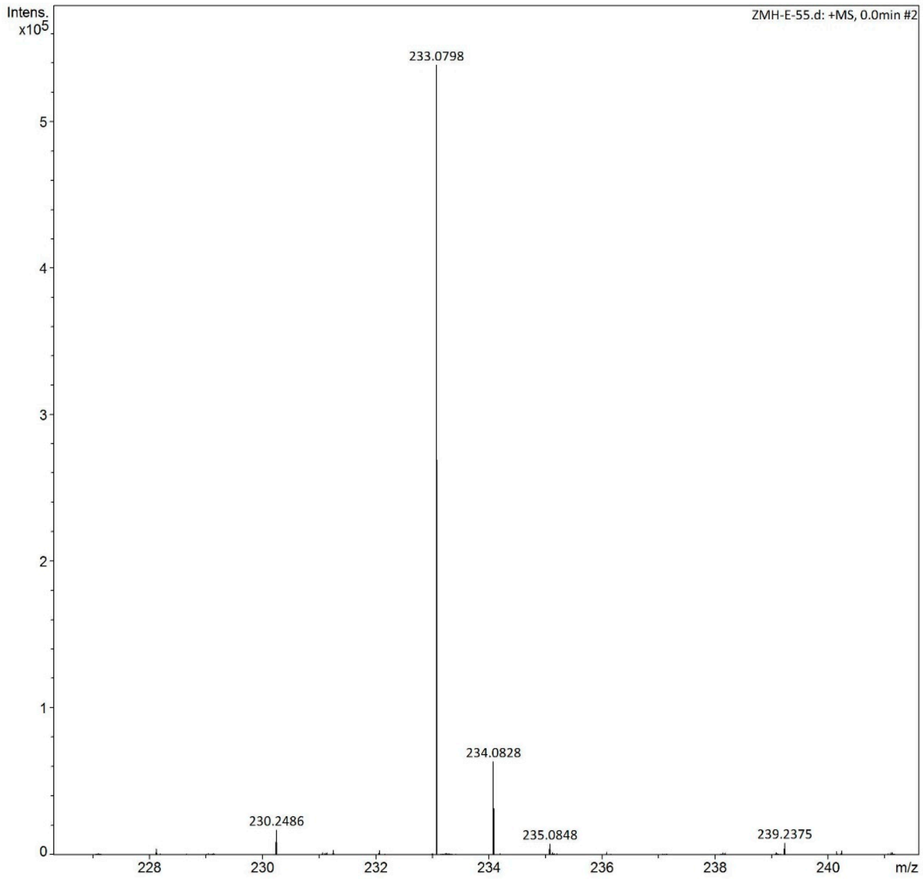

Figure S48. HR-ESI-MS spectrum of compound **5**

Thermo Scientific ~ VISIONpro SOFTWARE V4.41

Operator Name	(None Entered)	Date of Report	2023/2/25
Department	(None Entered)	Time of Report	17:45:45下午
Organization	(None Entered)		
Information	(None Entered)		

Scan Graph

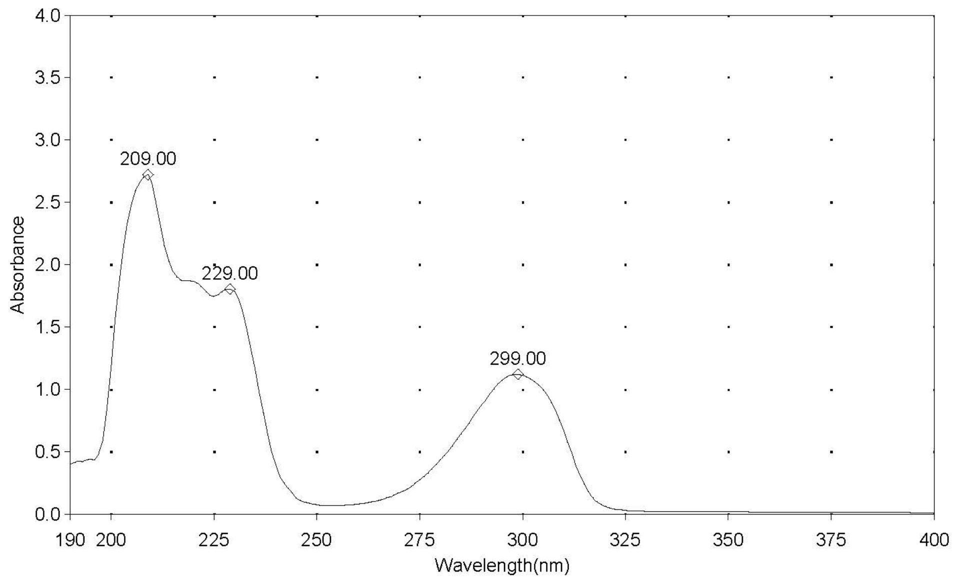

Results Table - scan003,ZMH-E-55,Cycle01

nm	A	**Peak Pick Method**
209.00	2.721	Find 8 Peaks Above -3.0000 A
229.00	1.802	Start Wavelength 190.00 nm
299.00	1.121	Stop Wavelength 400.00 nm
		Sort By Wavelength
Sensitivity	Medium	

Page 1, Scan Graph

Figure S49. UV spectrum of **5**

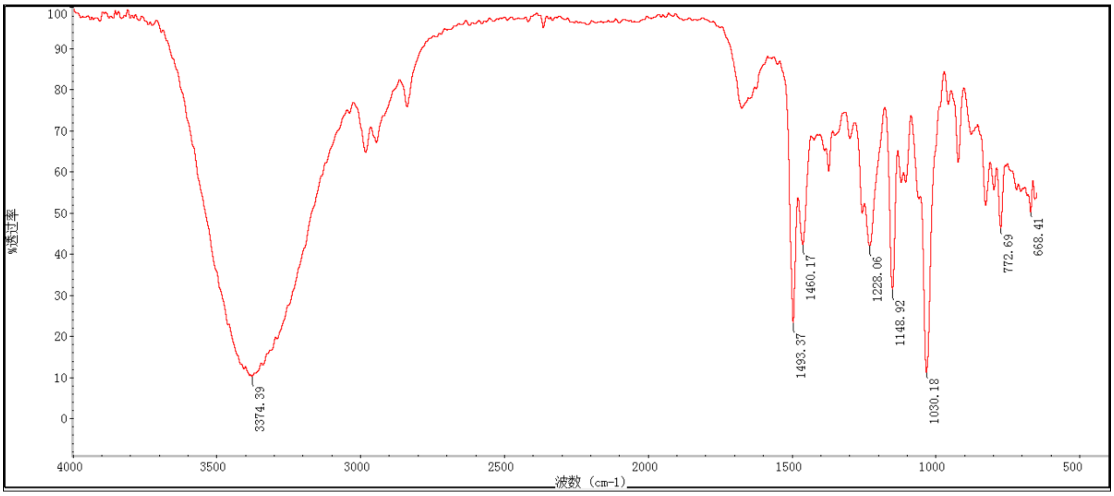

Figure S50. IR spectrum of **5**

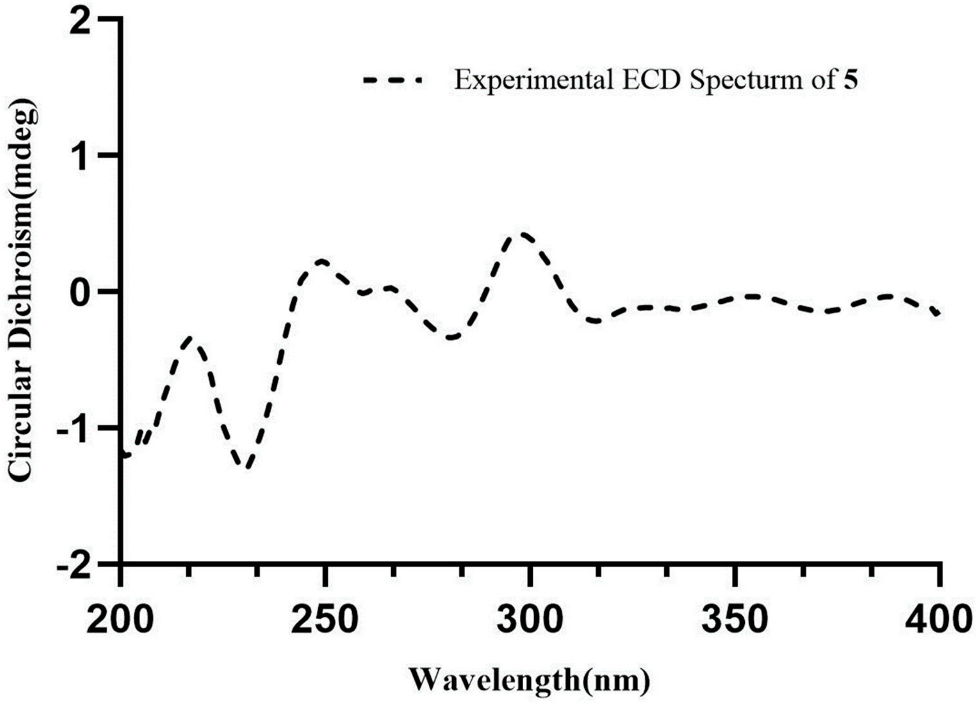

Figure S51. Experimental ECD spectrum of **5**

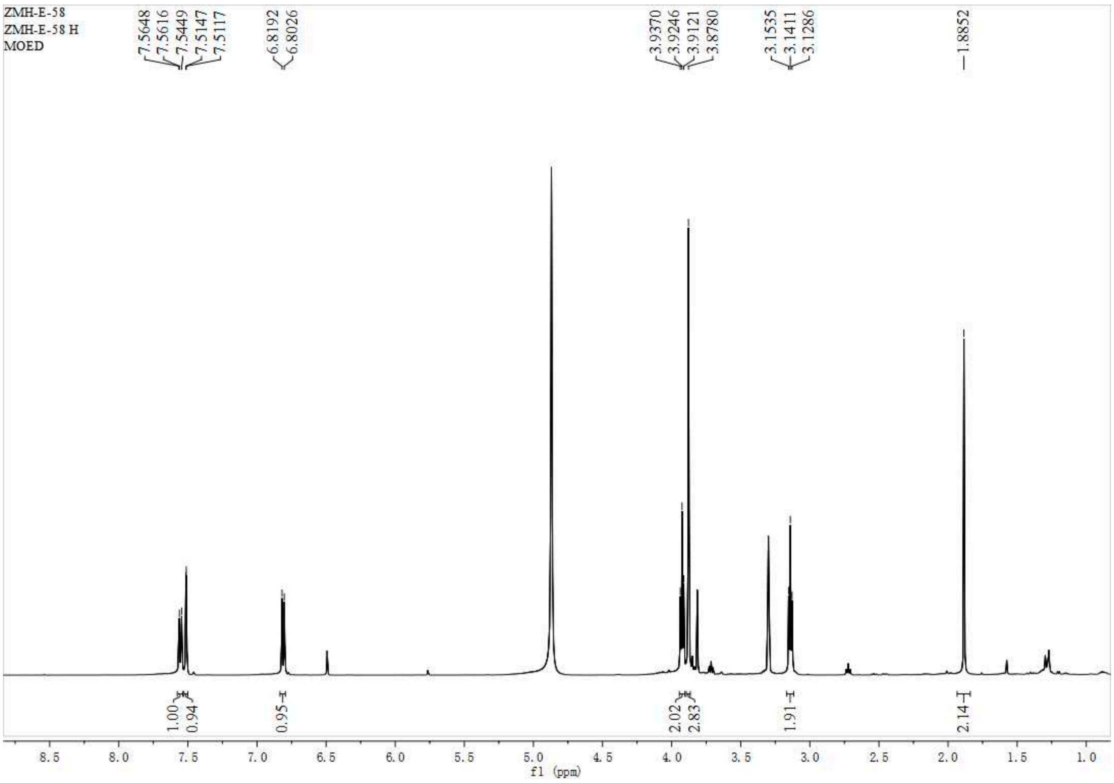

Figure S52. ^{1}H NMR spectrum (500MHz, CD_3OD) of **6**

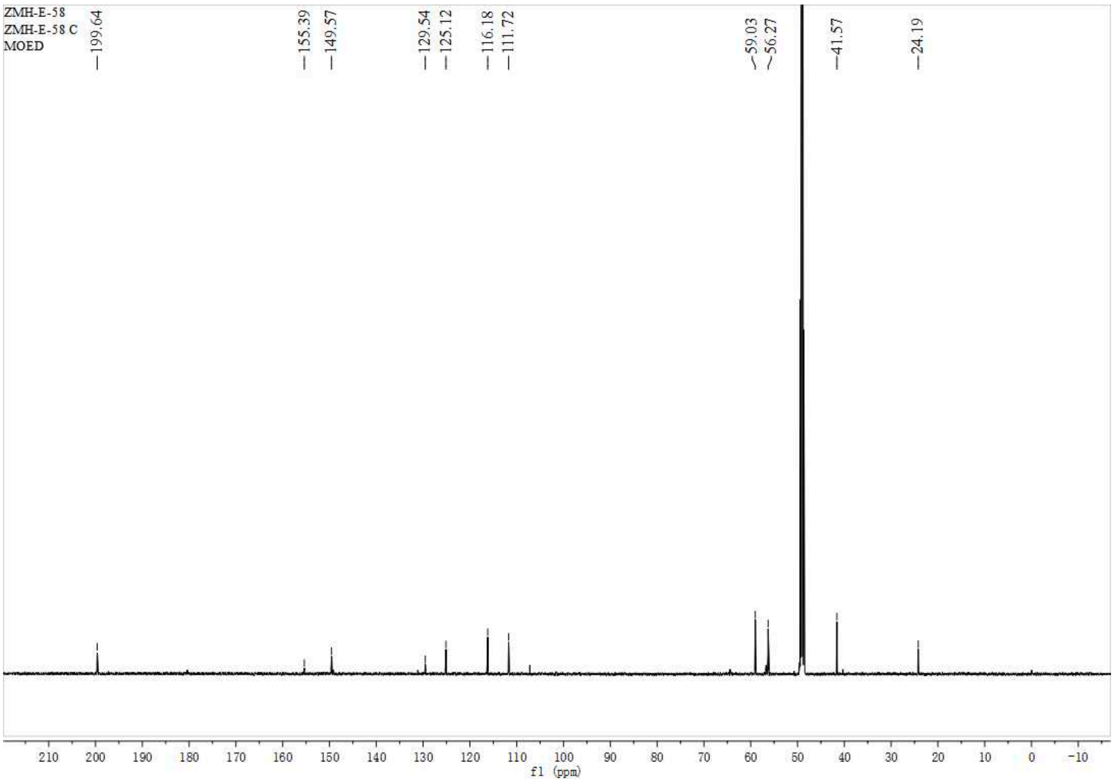

Figure S53. ^{13}C NMR spectrum (125MHz, CD_3OD) of **6**

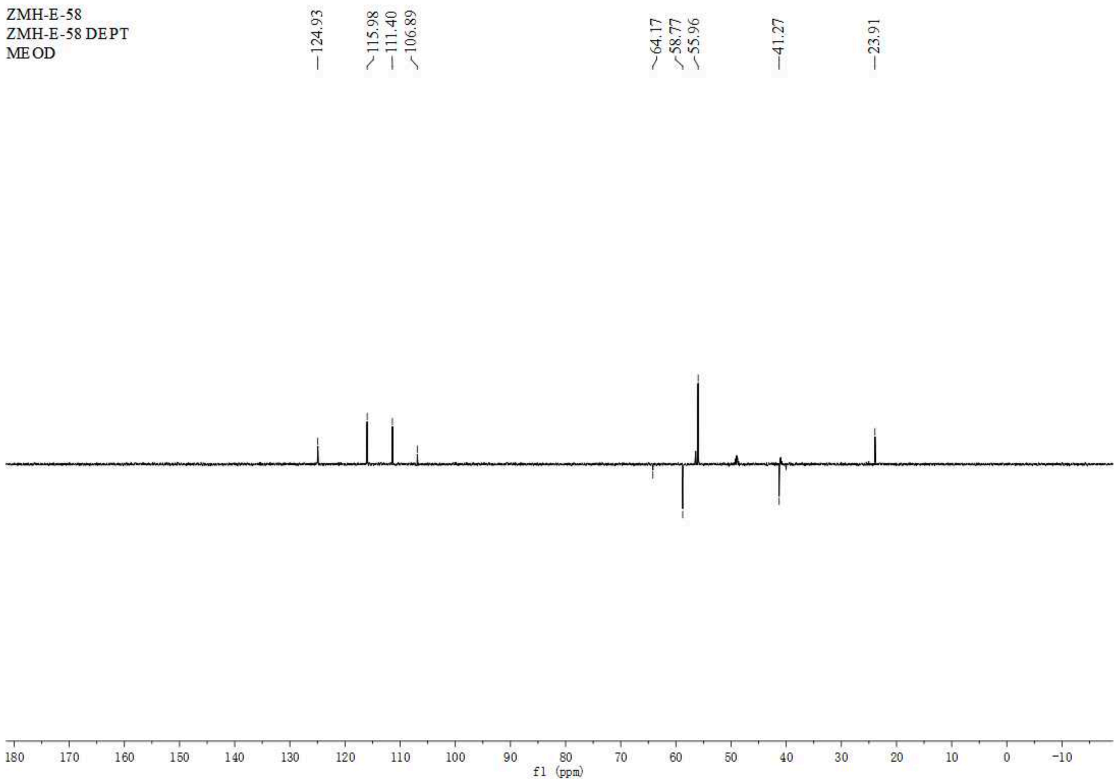

Figure S54. DEPT spectrum of **6**

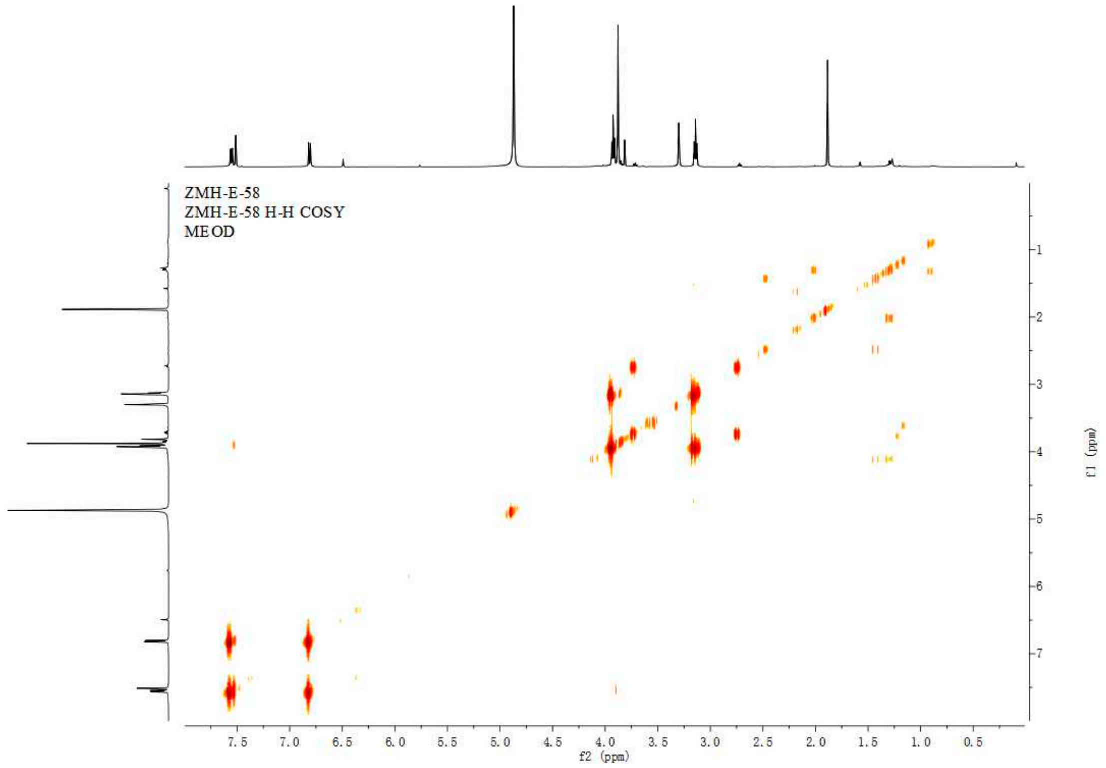

Figure S55. ^{1}H-^{1}H COSY spectrum of **6**

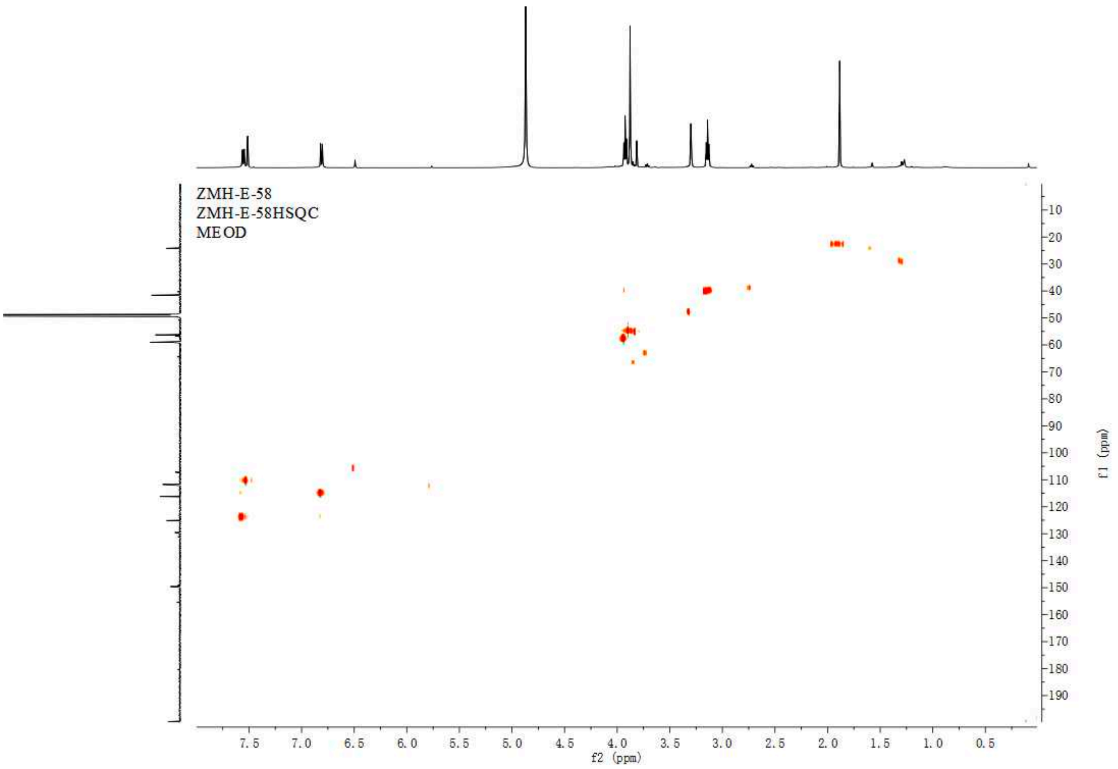

Figure S56. HSQC spectrum of **6**

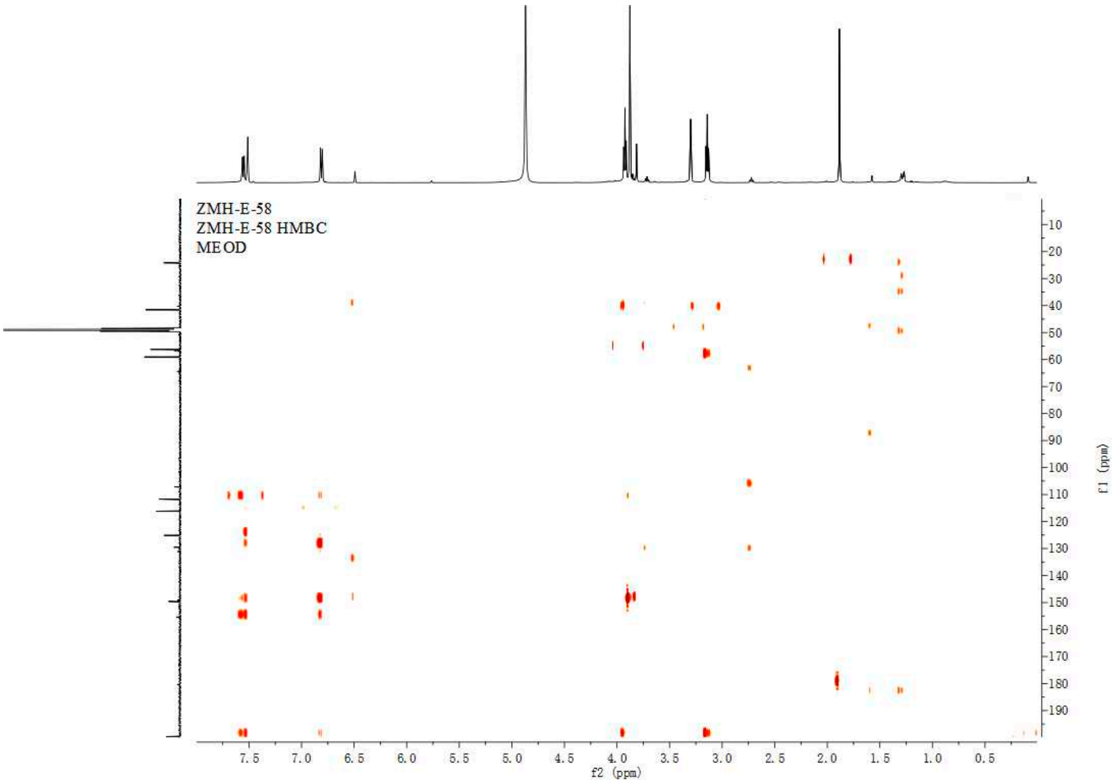

Figure S57. HMBC spectrum of **6**

Display Report

Analysis Info

Analysis Name	D:\Data\GJH\FXL\ZMH-E-58.d	Acquisition Date	2/10/2023 4:51:08 PM
Method	tune_pos_standard_20141031.m	Operator	Demo User
Sample Name	ZMH-E-58	Instrument	maXis HD 1820881.21303
Comment			

Acquisition Parameter

Source Type	ESI	Ion Polarity	Positive	Set Nebulizer	0.3 Bar
Focus	Active	Set Capillary	3500 V	Set Dry Heater	200 °C
Scan Begin	50 m/z	Set End Plate Offset	-500 V	Set Dry Gas	4.0 l/min
Scan End	3000 m/z	Set Charging Voltage	2000 V	Set Divert Valve	Waste
		Set Corona	0 nA	Set APCI Heater	0 °C

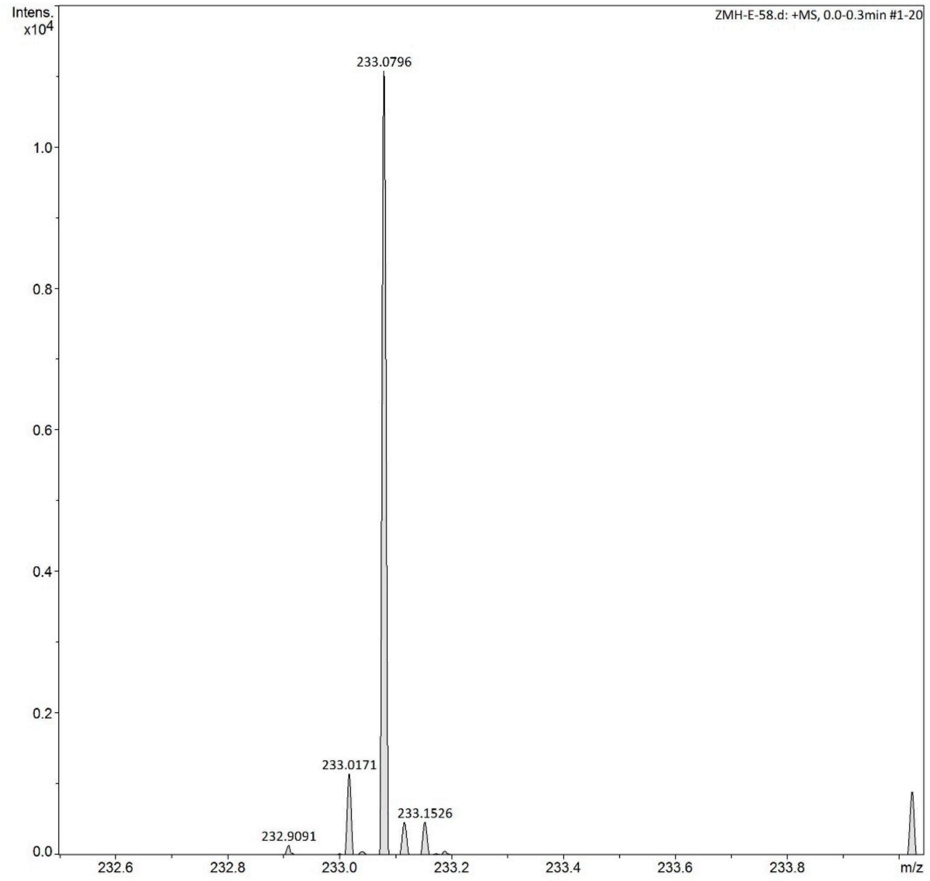

ZMH-E-58.d

Bruker Compass DataAnalysis 4.4 printed: 2/11/2023 4:44:54 PM by: demo Page 1 of 1

Figure S58. HR-ESI-MS spectrum of compound **6**

Thermo Scientific ~ VISIONpro SOFTWARE V4.41

Operator Name	(None Entered)	Date of Report	2023/2/25
Department	(None Entered)	Time of Report	18:33:23下午
Organization	(None Entered)		
Information	(None Entered)		

Scan Graph

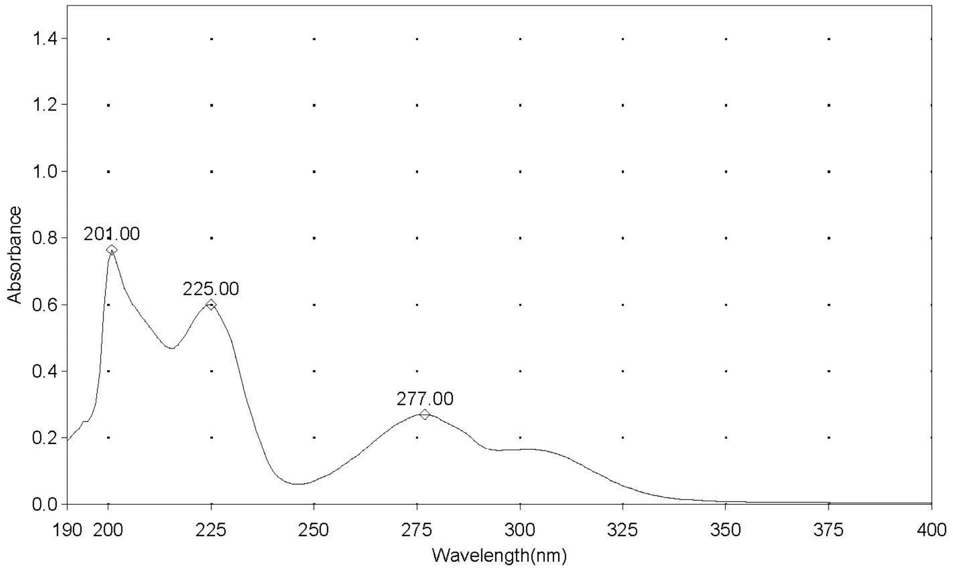

Results Table - scan012,ZMH-E-58,Cycle01

nm	A	**Peak Pick Method**
201.00	.765	Find 8 Peaks Above -3.0000 A
225.00	.599	Start Wavelength 190.00 nm
277.00	.270	Stop Wavelength 400.00 nm
		Sort By Wavelength
Sensitivity	Medium	

Page 1, Scan Graph

Figure S59. UV spectrum of **6**

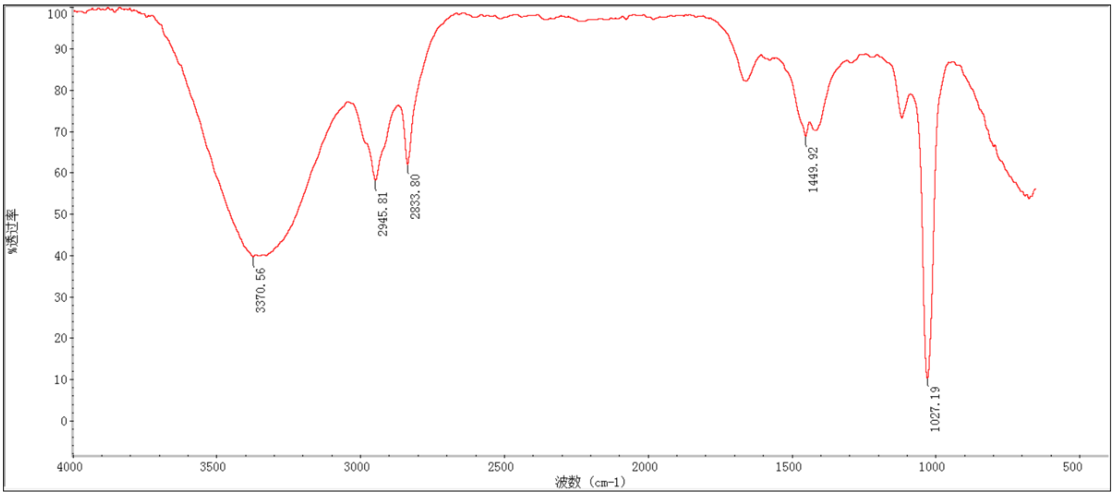

Figure S60. IR spectrum of **6**

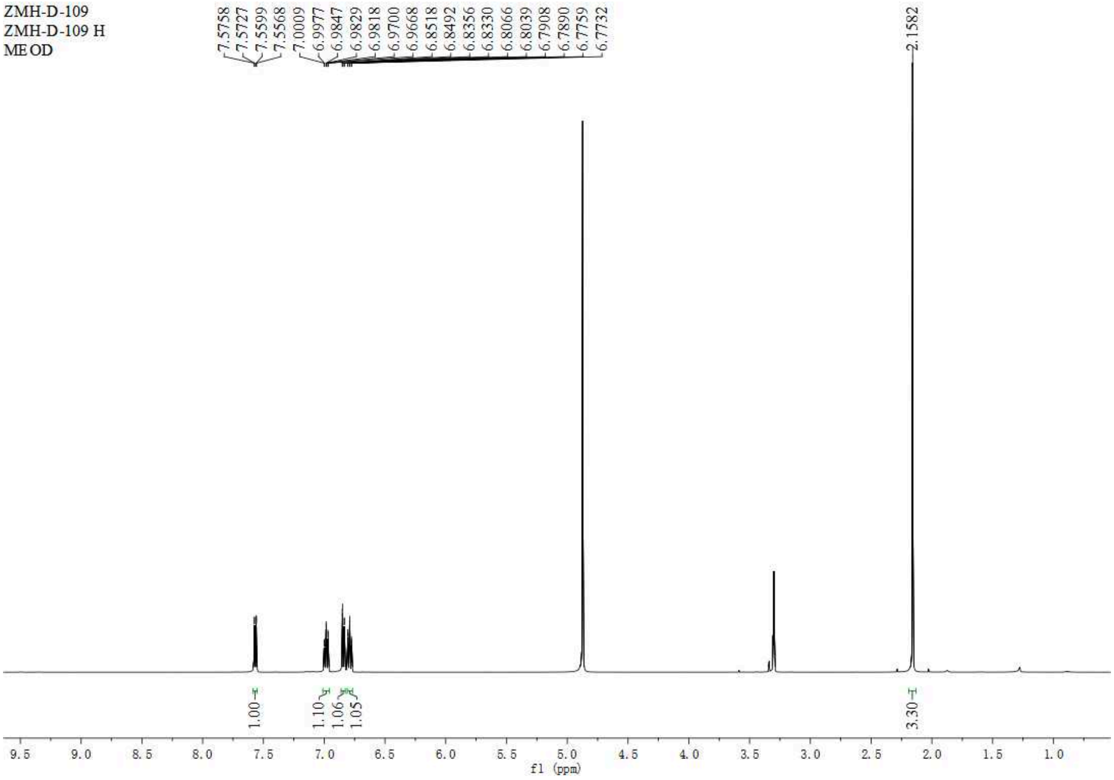

Figure S61. ^{1}H NMR spectrum (500MHz, CD_3OD) of **7**

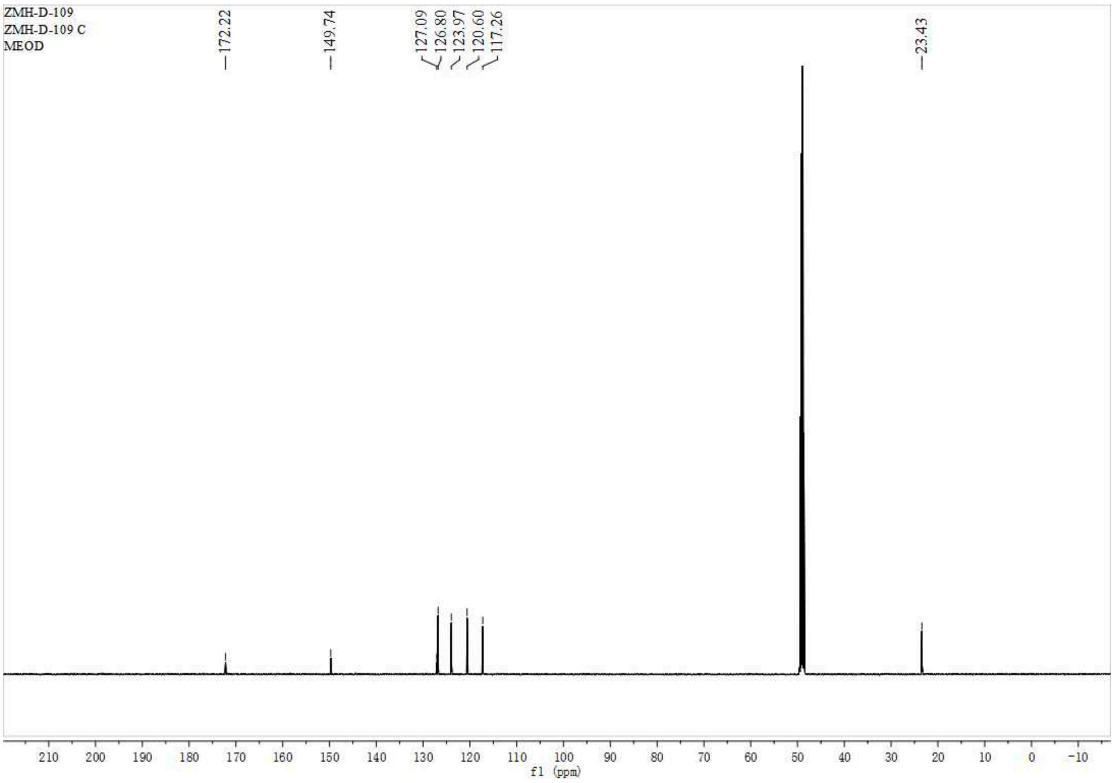

Figure S62. ^{13}C NMR spectrum (125MHz, CD_3OD) of **7**

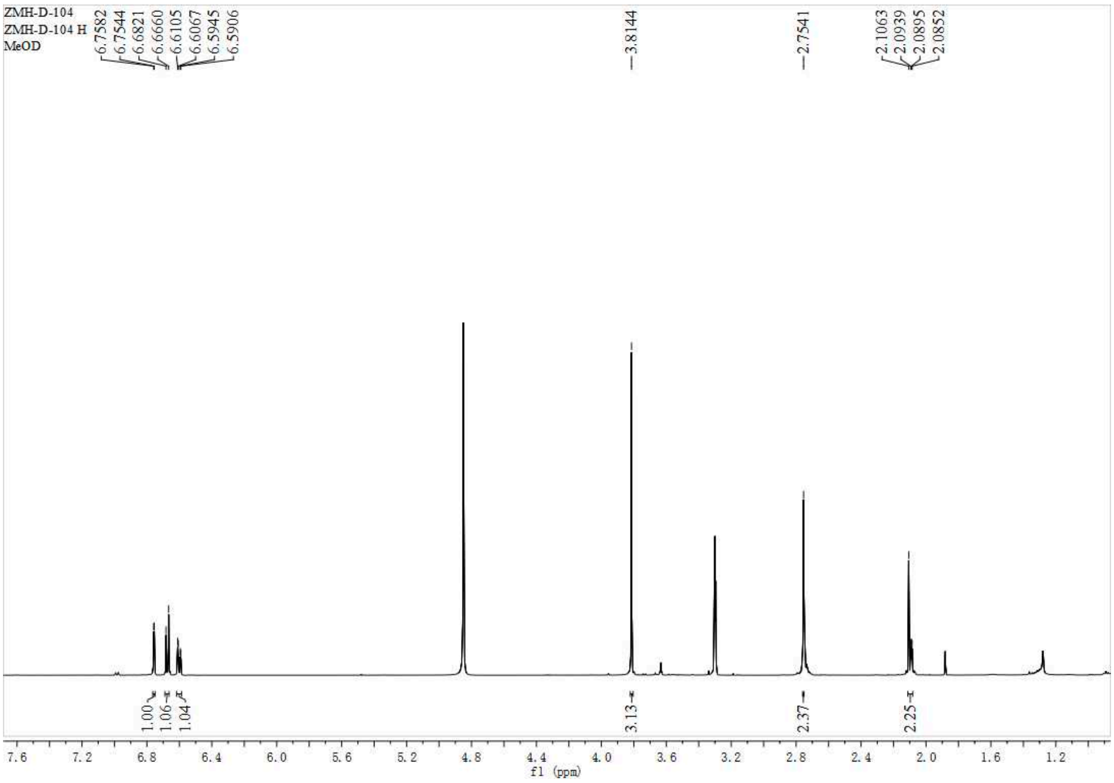

Figure S63. ^{1}H NMR spectrum (500MHz, CD_3OD) of **8**

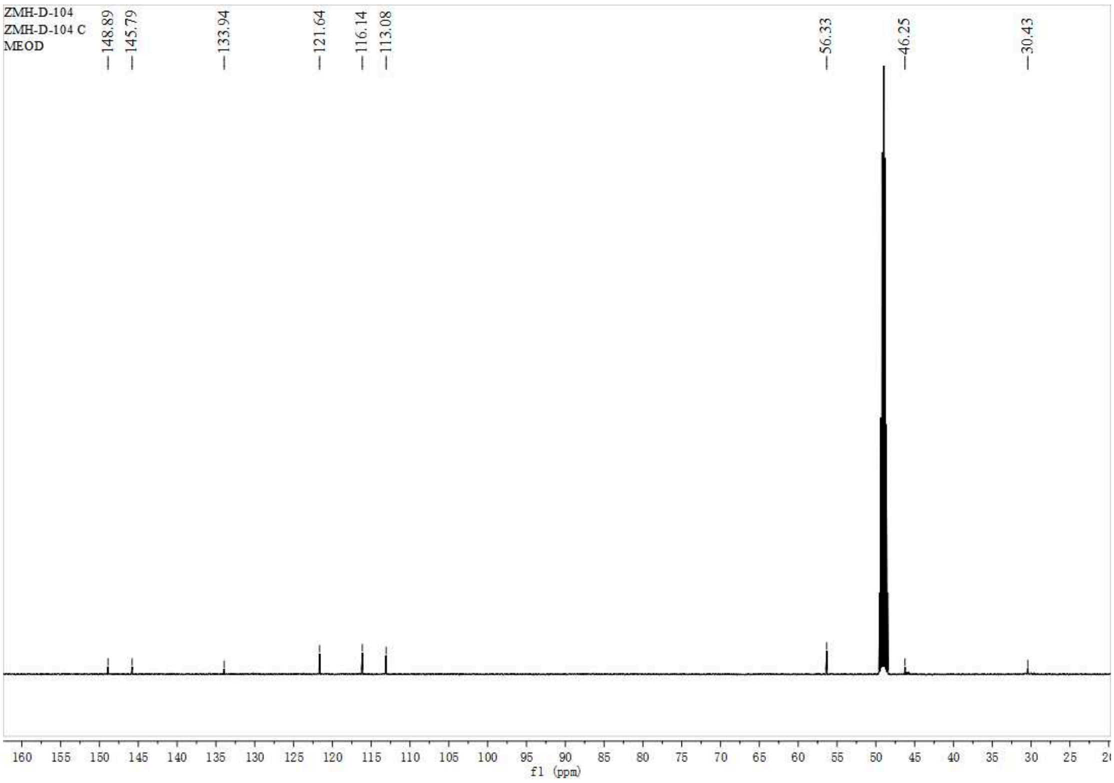

Figure S64. ^{13}C NMR spectrum (125MHz, CD_3OD) of **8**

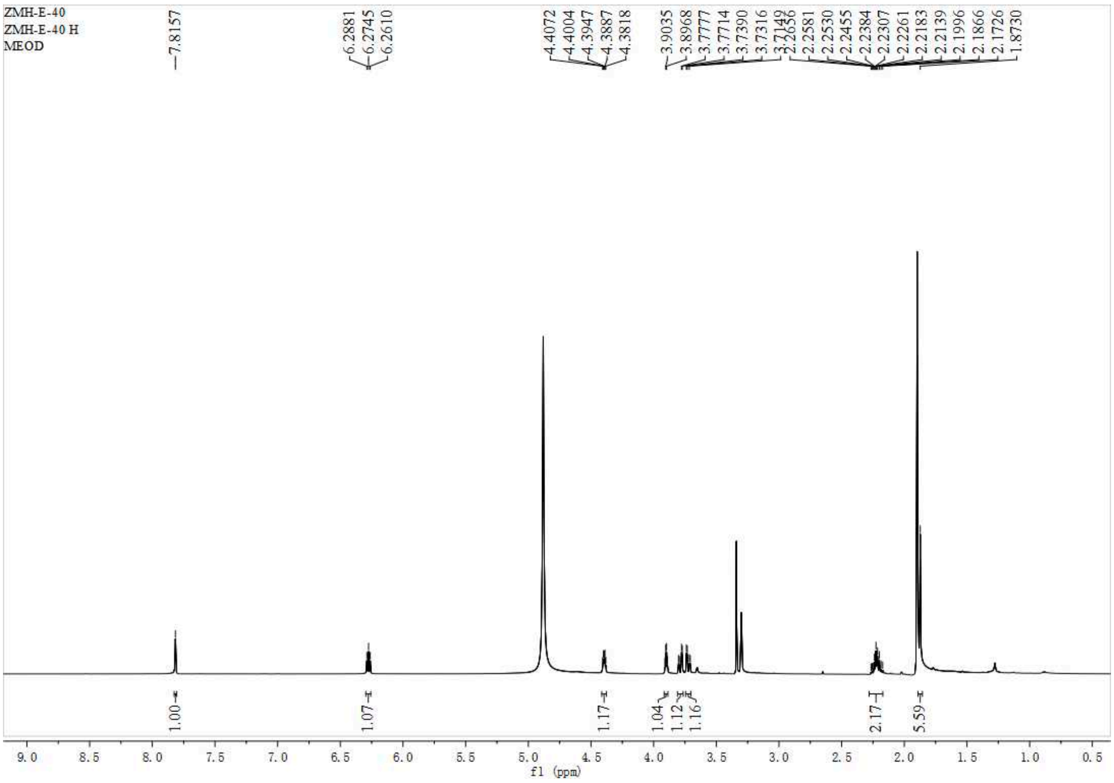

Figure S65. ^{1}H NMR spectrum (500MHz, CD_3OD) of **9**

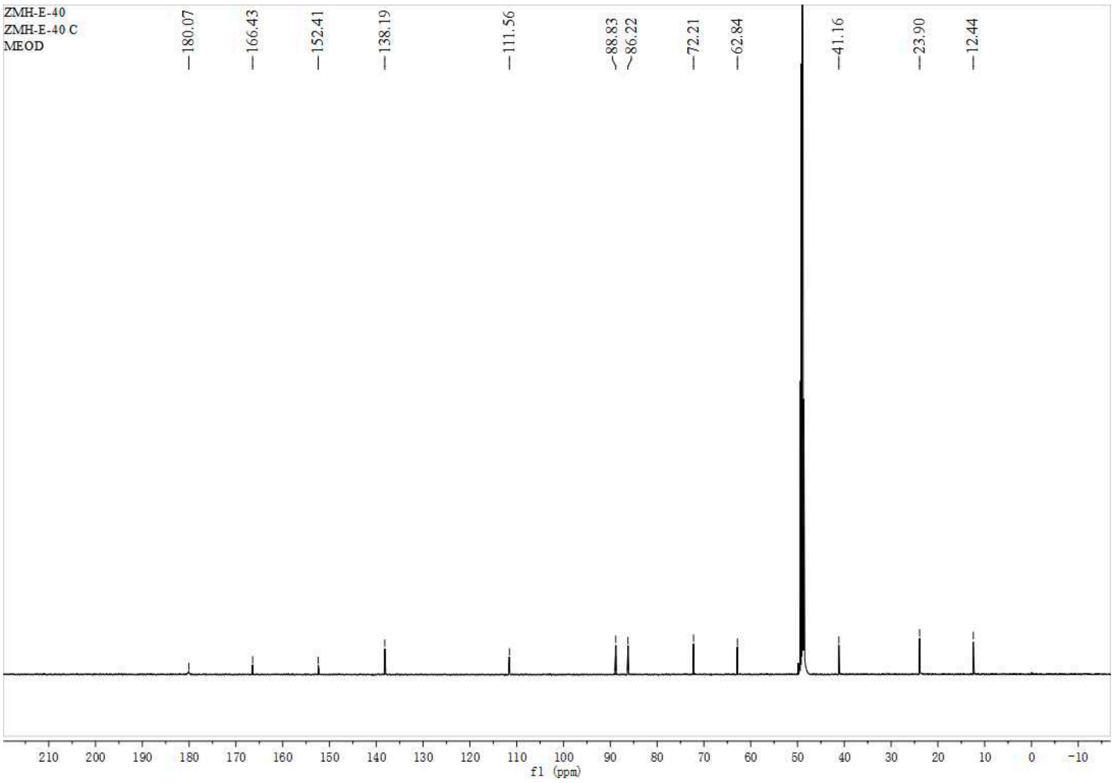

Figure S66. ^{13}C NMR spectrum (125MHz, CD_3OD) of **9**

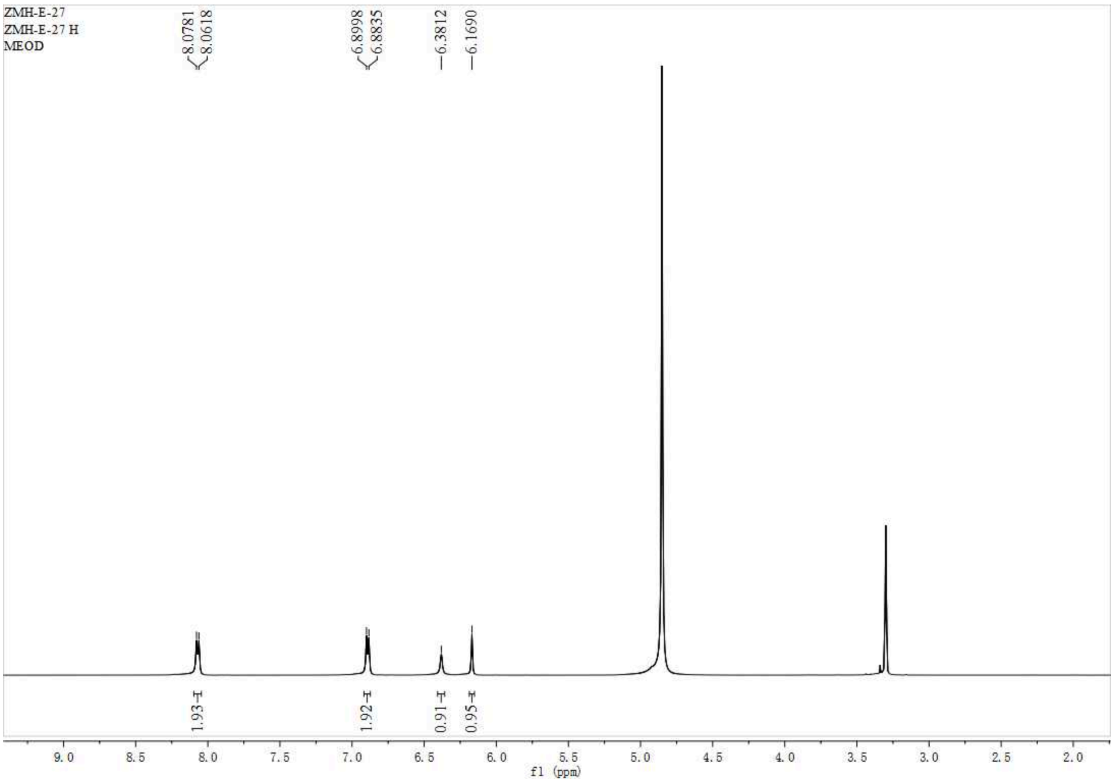

Figure S67. ^{1}H NMR spectrum (500MHz, CD_3OD) of **10**

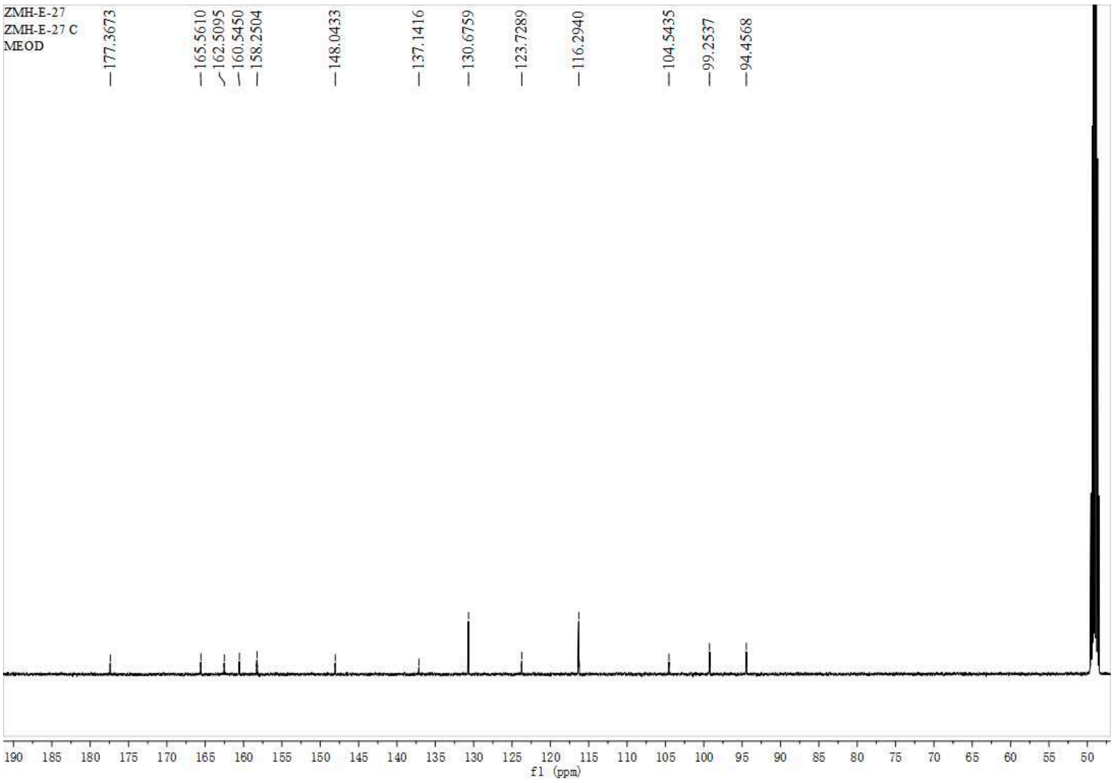

Figure S68. ^{13}C NMR spectrum (125MHz, CD_3OD) of **10**

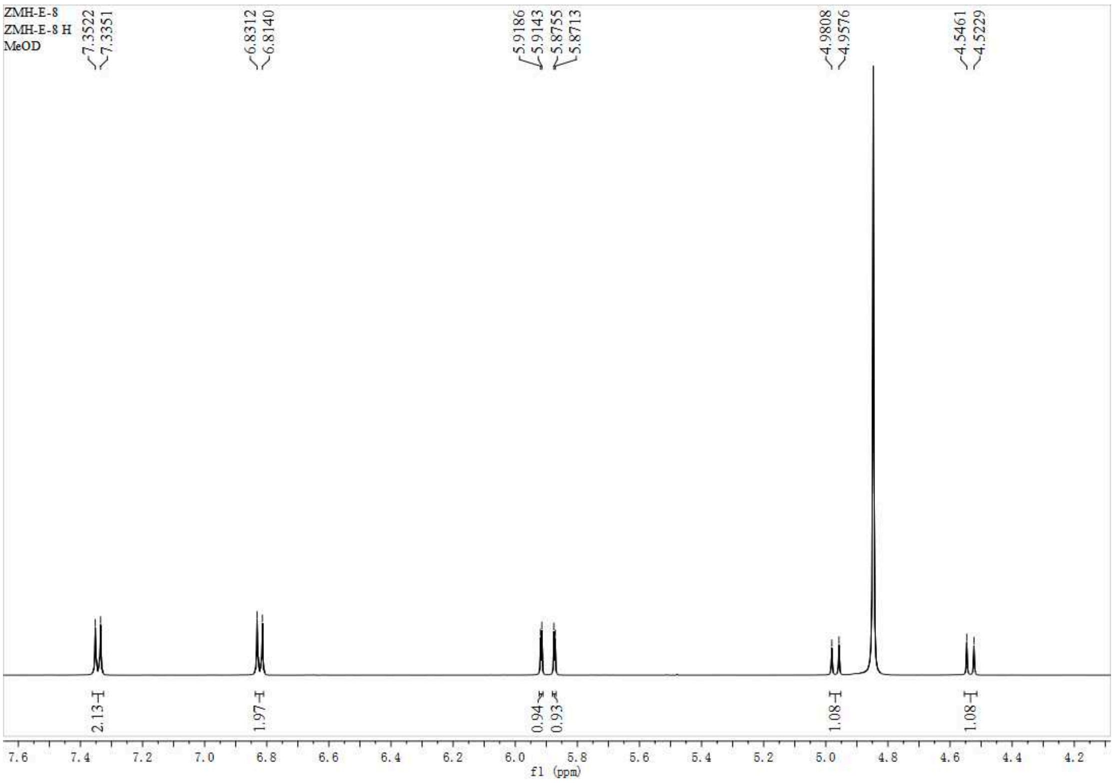

Figure S69. ^{1}H NMR spectrum (500MHz, CD_3OD) of **11**

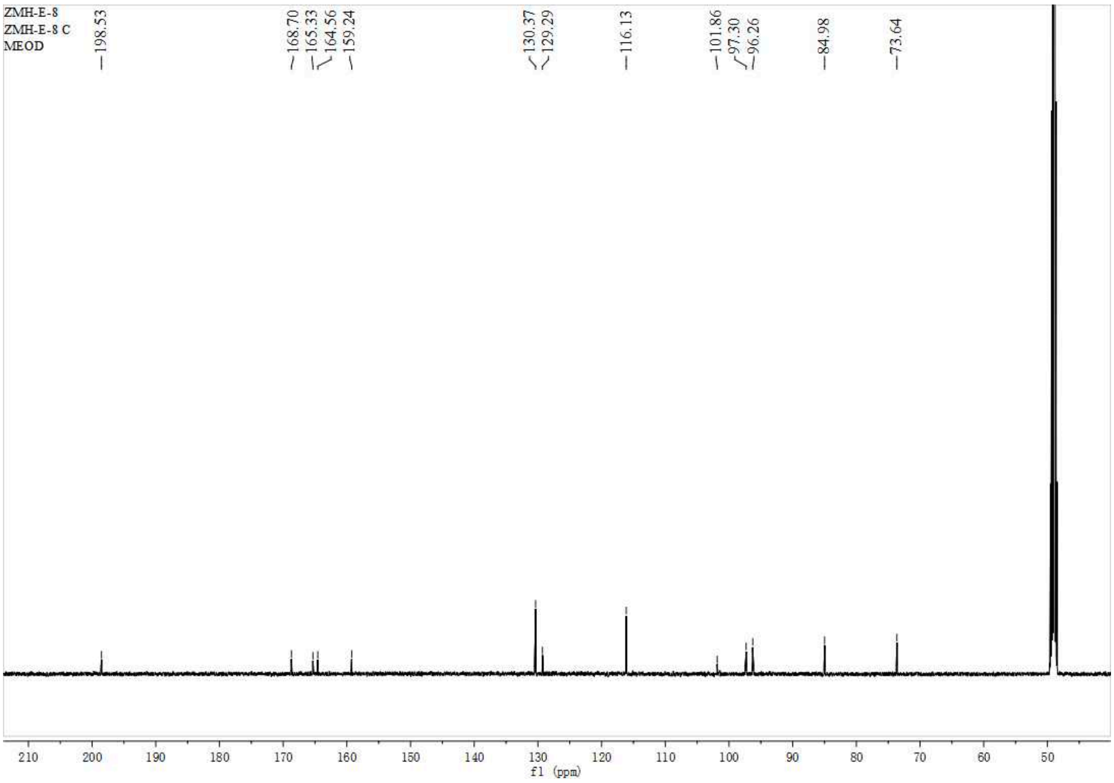

Figure S70. ^{13}C NMR spectrum (125MHz, CD_3OD) of **11**

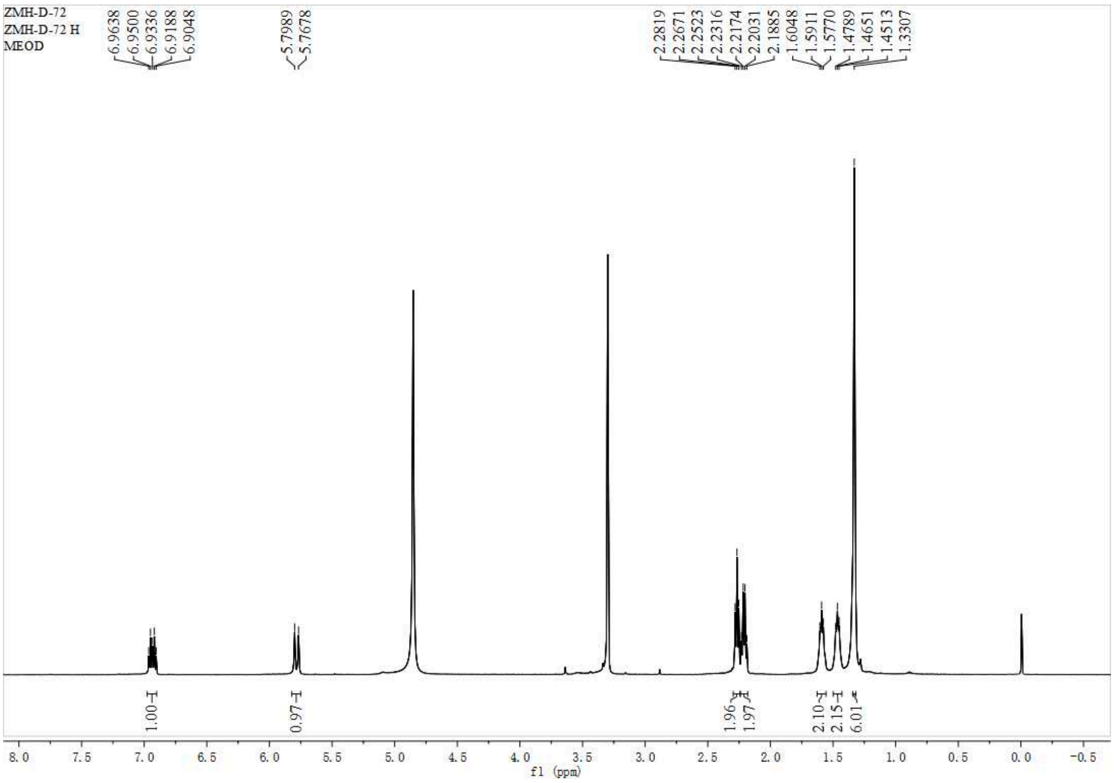

Figure S71. ^{1}H NMR spectrum (500MHz, CD_3OD) of **12**

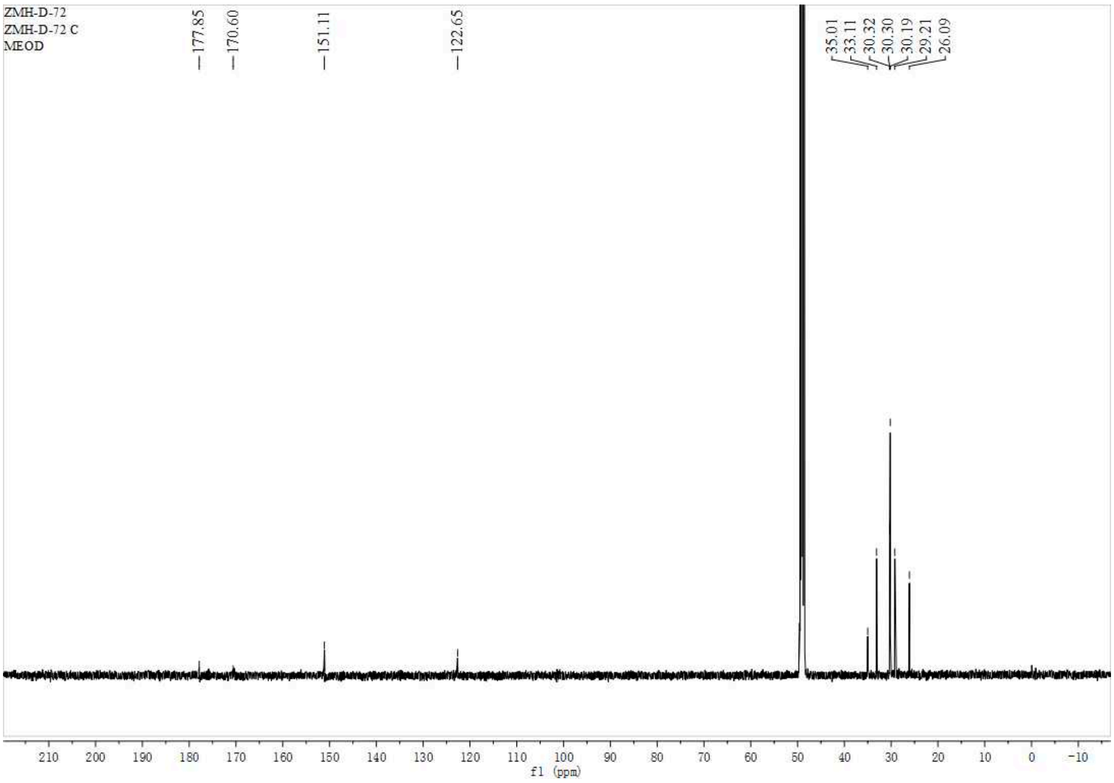

Figure S72. ^{13}C NMR spectrum (125MHz, CD_3OD) of **12**

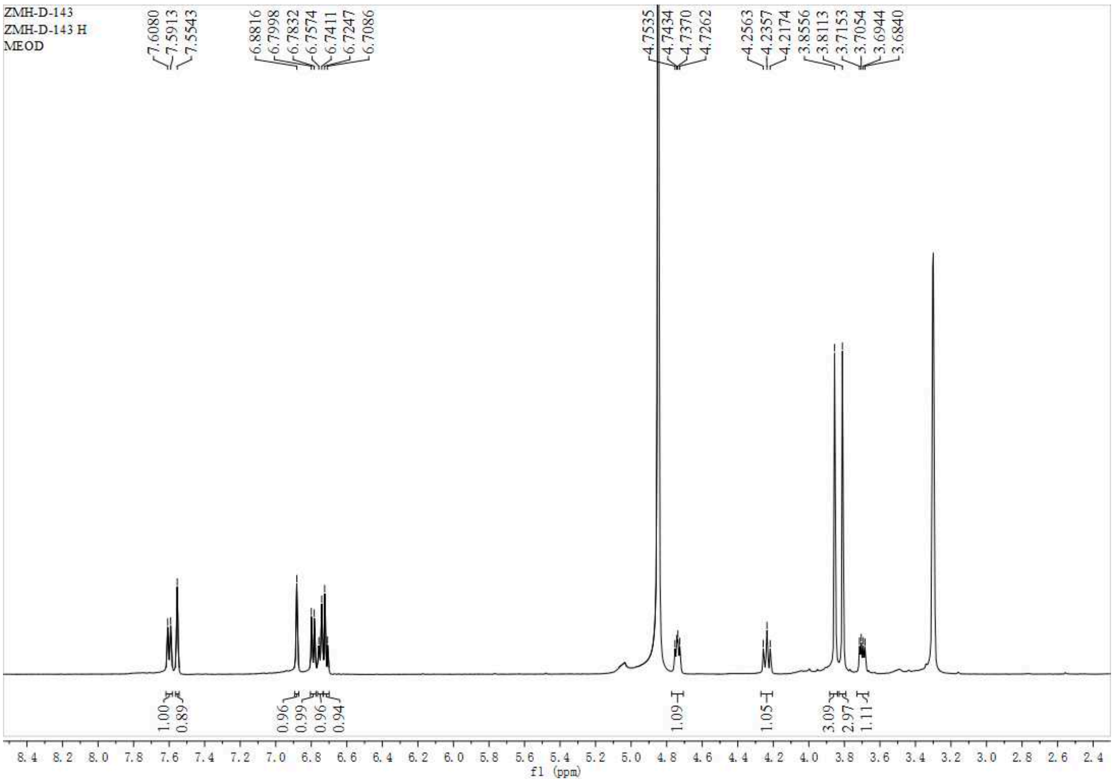

Figure S73. ^{1}H NMR spectrum (500MHz, CD_3OD) of **13**

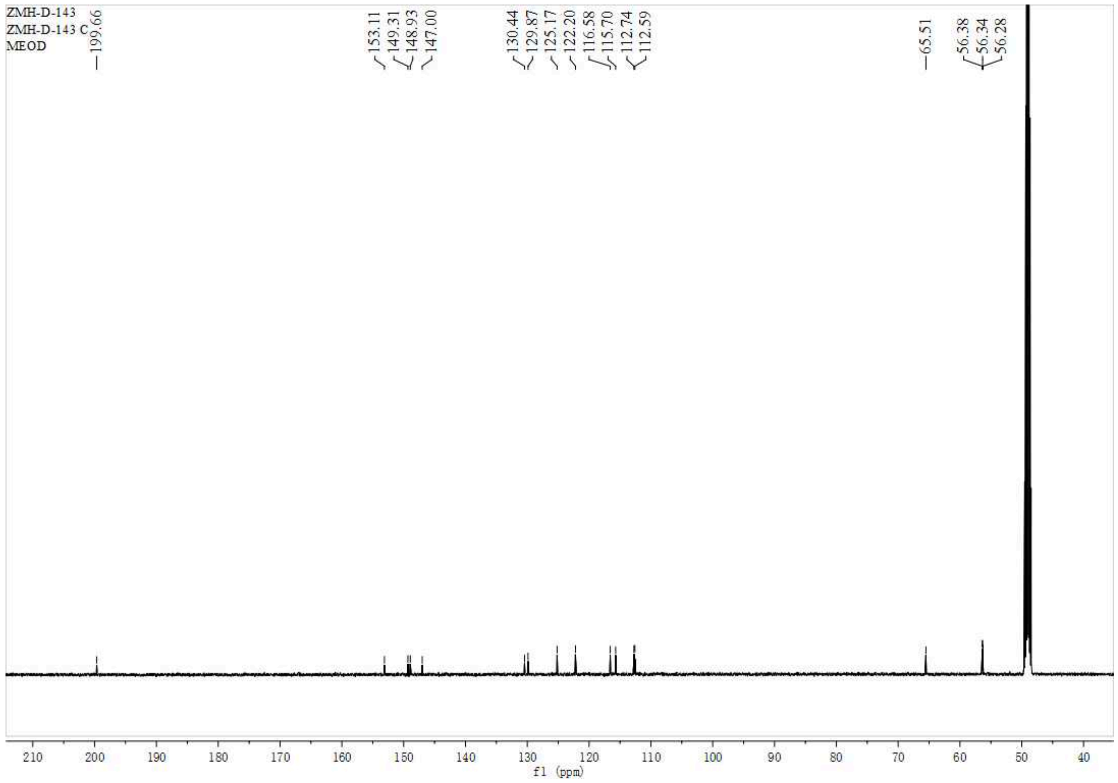

Figure S74. ^{13}C NMR spectrum (125MHz, CD_3OD) of **13**

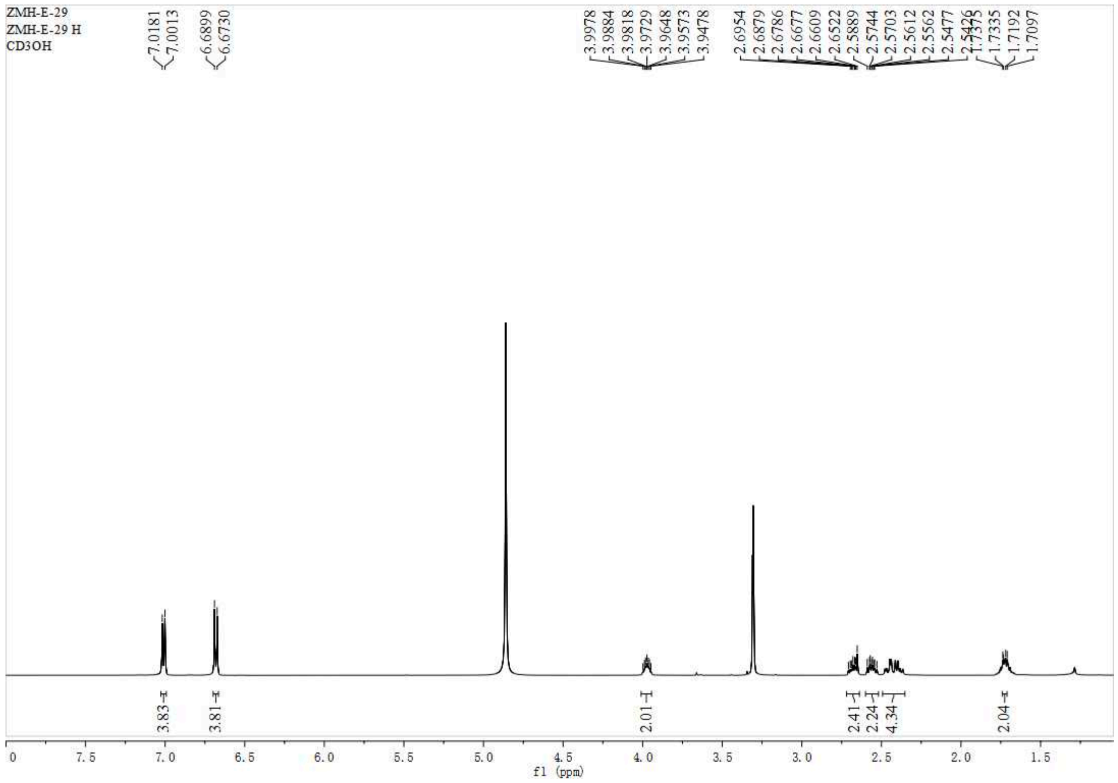

Figure S75. ^{1}H NMR spectrum (500MHz, CD_3OD) of **14**

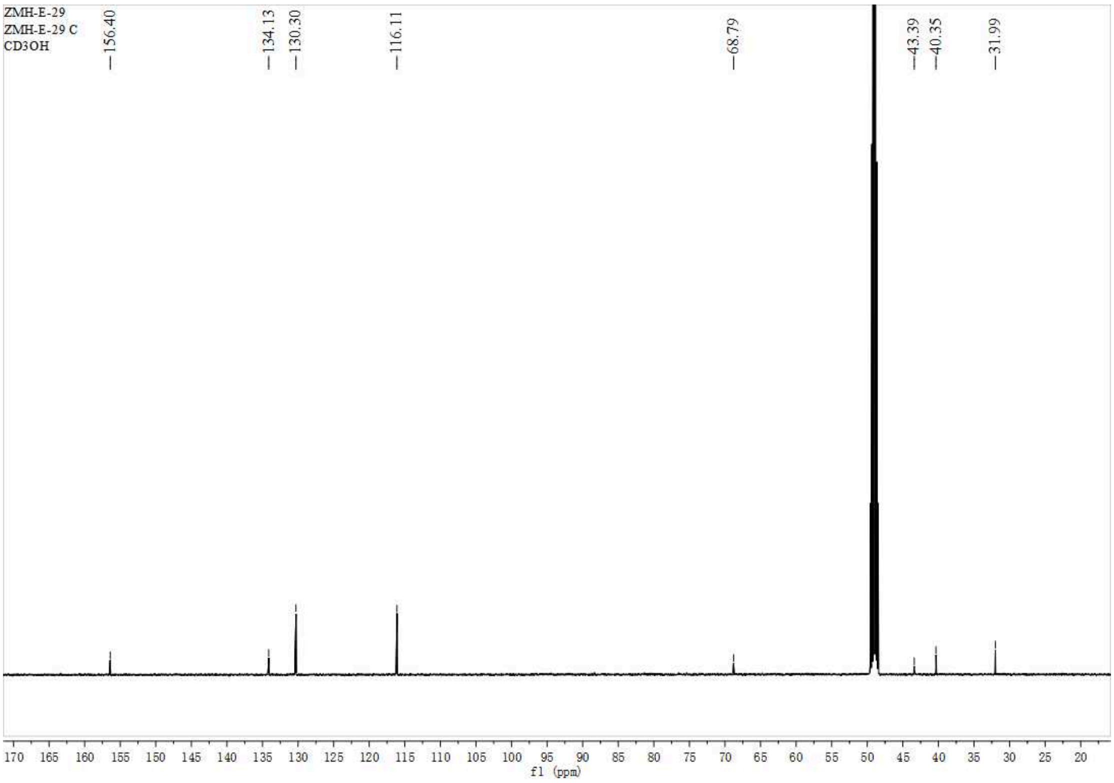

Figure S76. ^{13}C NMR spectrum (125MHz, CD_3OD) of **14**

Table S1. Energy for ECD calculation of compound **1–5**.

Index	Electronic Energy (EE)			
	**1**	**2**	**3**	**4**
1	-1454.733764	-1533.40806	-955.074298	-881.226129
2	-1454.734271	-1533.408567	-955.074298	-881.226323
3	-1454.734431	-1533.408807	-955.074337	-881.227024
4	-1454.734431	-1533.408892	-955.074481	-881.227188
5	-1454.73488	-1533.408987	-955.074481	-881.227333
6	-1454.735055	-1533.409567	-955.07452	-881.227364
7	-1454.735455	-1533.409617	-955.07452	-881.22884
8	-1454.735576	-1533.409751		-881.229084
9	-1454.735663	-1533.409918		
10	-1454.735719	-1533.410715		
11	-1454.736196	-1533.411167		
12	-1454.736196	-1533.411314		
13	-1454.736317	-1533.411314		
14	-1454.736317	-1533.411314		
15	-1454.736359			
16	-1454.736485			
17	-1454.736491			
